# RNA-Seq analysis reveals insight into enhanced rice *Xa7-*mediated bacterial blight resistance at high temperature

**DOI:** 10.1371/journal.pone.0187625

**Published:** 2017-11-06

**Authors:** Stephen P. Cohen, Hongxia Liu, Cristiana T. Argueso, Andy Pereira, Casiana Vera Cruz, Valerie Verdier, Jan E. Leach

**Affiliations:** 1 Department of Bioagricultural Sciences and Pest Management, Colorado State University, Fort Collins, Colorado, United States of America; 2 Cell and Molecular Biology Graduate Program, Colorado State University, Fort Collins, Colorado, United States of America; 3 Institute of Crop Sciences, Chinese Academy of Agricultural Sciences, Beijing, China; 4 Department of Crop, Soil and Environmental Sciences, University of Arkansas, Fayetteville, Arkansas, United States of America; 5 Plant Breeding, Genetics and Biotechnology Division, International Rice Research Institute, Los Baños, Philippines; 6 IRD, Cirad, Univ Montpellier, IPME, Montpellier, France; Kyung Hee Univeristy, REPUBLIC OF KOREA

## Abstract

Plant disease is a major challenge to agriculture worldwide, and it is exacerbated by abiotic environmental factors. During some plant-pathogen interactions, heat stress allows pathogens to overcome host resistance, a phenomenon which could severely impact crop productivity considering the global warming trends associated with climate change. Despite the importance of this phenomenon, little is known about the underlying molecular mechanisms. To better understand host plant responses during simultaneous heat and pathogen stress, we conducted a transcriptomics experiment for rice plants (cultivar IRBB61) containing *Xa7*, a bacterial blight disease resistance (*R*) gene, that were infected with *Xanthomonas oryzae*, the bacterial blight pathogen of rice, during high temperature stress. *Xa7-*mediated resistance is unusual relative to resistance mediated by other *R* genes in that it functions better at high temperatures. Using RNA-Seq technology, we identified 8,499 differentially expressed genes as temperature responsive in rice cultivar IRBB61 experiencing susceptible and resistant interactions across three time points. Notably, genes in the plant hormone abscisic acid biosynthesis and response pathways were up-regulated by high temperature in both mock-treated plants and plants experiencing a susceptible interaction and were suppressed by high temperature in plants exhibiting *Xa7-*mediated resistance. Genes responsive to salicylic acid, an important plant hormone for disease resistance, were down-regulated by high temperature during both the susceptible and resistant interactions, suggesting that enhanced *Xa7-*mediated resistance at high temperature is not dependent on salicylic acid signaling. A DNA sequence motif similar to known abscisic acid-responsive *cis-*regulatory elements was identified in the promoter region upstream of genes up-regulated in susceptible but down-regulated in resistant interactions. The results of our study suggest that the plant hormone abscisic acid is an important node for cross-talk between plant transcriptional response pathways to high temperature stress and pathogen attack. Genes in this pathway represent an important focus for future study to determine how plants evolved to deal with simultaneous abiotic and biotic stresses.

## Introduction

Plant diseases are a major detriment to global food production, accounting for an estimated 10% or more of crop yield loss each year [1]. The disease phenotype is mediated by pathogen and host genotypes as well as environmental conditions, and these factors ultimately determine whether a plant succumbs to disease [2, 3]. Environmental stresses can negatively impact a plant’s ability to respond to pathogen attack, increasing disease severity [4, 5]. This is due in part to cross-talk among the highly complex and intertwined plant stress signaling pathways [6, 7]. Heat stress can reduce the effectiveness of plant disease resistance, rendering agriculturally important plants susceptible to attack [8–12]. While this phenomenon could pose a serious risk to food security in light of climate variability and global warming trends, current insight into specific underlying mechanisms of increased disease and/or loss of disease resistance at high temperature is lacking. Elucidation of these mechanisms would inform novel crop breeding strategies and reduce global food losses due to temperature-induced disease.

Bacteria in the *Xanthomonas oryzae* (*Xo*) group are pathogenic to rice and cause considerable yield loss every year [13]. *Xo* is most effectively controlled through the development of resistant rice varieties, particularly through deployment of single gene resistance [14]. However, many rice resistance (*R*) genes lose function at higher temperatures, leading to increased bacterial blight disease caused by the *Xo* pathovar *oryzae* [15]. Resistance genes in other plants similarly lose function, such as the Arabidopsis *R*-like gene *SNC1* and the tobacco *N* gene, an *R-*gene to tobacco mosaic virus [10]. One rice bacterial blight *R-*gene (*Xa7*) retains function at high temperature [15]. Unusually, *Xa7* not only retains function, but also functions better at high temperature, both in long-lasting field trials, and at least up to 14 days post-inoculation in laboratory experiments. When triggered by the cognate pathogen effector protein AvrXa7, *Xa7* induces the hypersensitive response, a rapid, localized host cell death that reduces pathogen spread in the host plant [16]. In addition to functioning better at high temperature, *Xa7* also retains function during drought stress, a condition in which other rice *R-*genes fail to function [17, 18], suggesting that the underlying mechanism of *Xa7* can overcome general abiotic stresses. Because *Xa7* is a durable, long-lasting resistance gene that is effective in growth chamber, greenhouse, and field studies [15, 19], understanding the mechanism underlying enhanced resistance at high temperature will be an asset to agricultural researchers and crop breeders.

Plants are sessile, so they must be versatile in their ability to adapt to a wide range of abiotic and biotic stresses [20]. Phytohormones are important regulators of plants’ abilities to detect and respond to stresses [20–22]. One critical phytohormone involved in plant adaptation to abiotic stresses is abscisic acid (ABA), which acts as a generic regulator for abiotic stress response [23]. During abiotic stress, ABA primarily regulates plant osmotic stress tolerance, through mechanisms such as closure of stomatal or expression of dehydration tolerance genes. During the rice-*Xo* interaction, exogenous treatment of ABA promotes rice susceptibility to *Xo* and acts as a cross-kingdom signal to promote bacterial swimming [24, 25]. The hormone salicylic acid (SA) plays an important role in rice defense against *Xo*, and exogenous application of SA promotes both basal defense and the hypersensitive response during the rice-*Xo* interaction [26]. Intriguingly, ABA and SA play antagonistic roles in rice [27, 28], suggesting a possible regulatory conflict during simultaneous abiotic and biotic stresses. Here we report the results of a transcriptomics study designed to determine early host changes during *Xa7-*mediated resistance in an effort to elucidate the mechanisms underlying enhanced resistance at moderately high temperatures.

## Results

### Acclimation to high temperature stress increases the effectiveness of *Xa7-*mediated resistance

**Fig 1:**
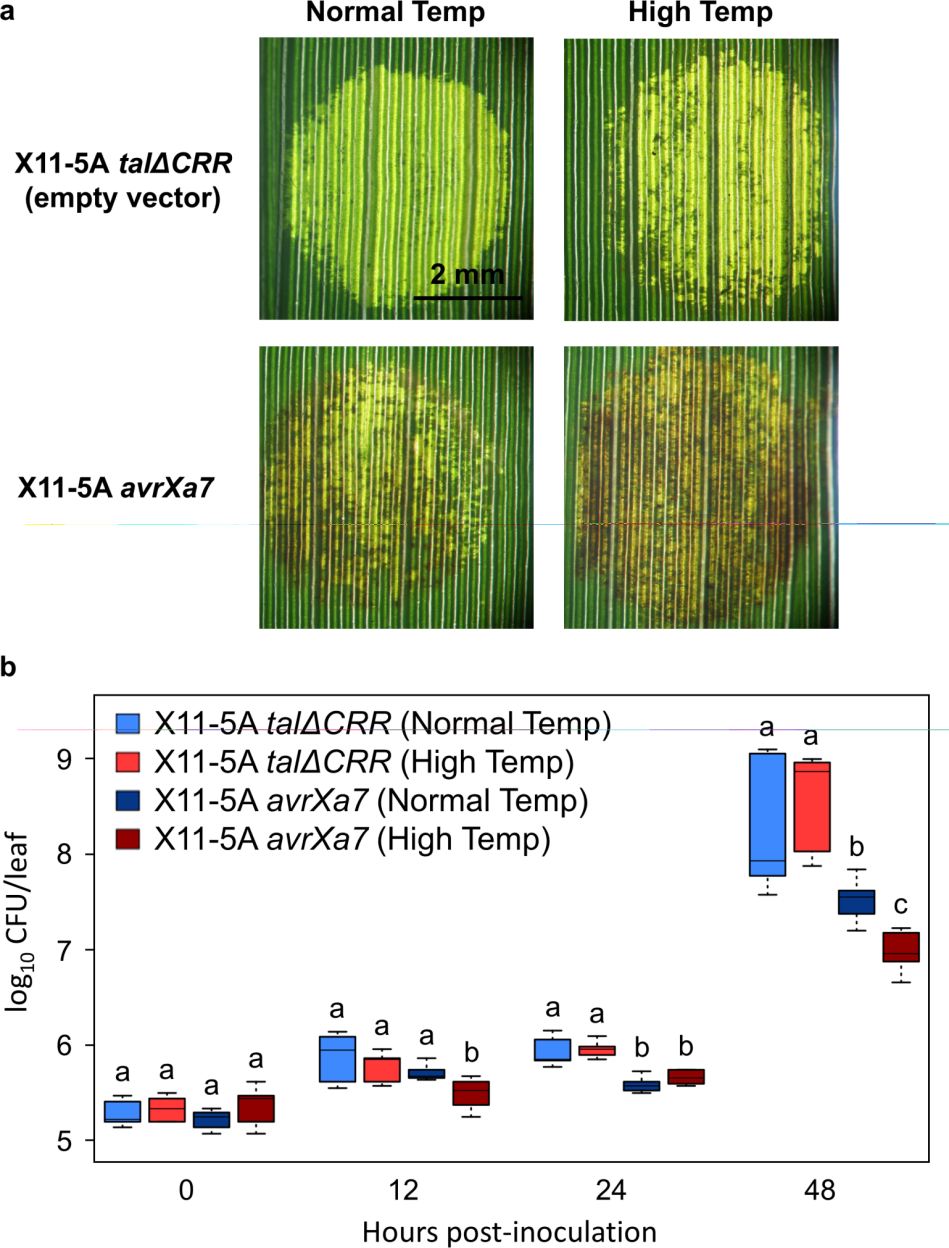
Rice displaying *Xa7-*mediated resistance is more resistant at high temperature. (a) Rice leaves displaying response to *Xo* strain X11-5A carrying either an empty vector (*talΔCRR*) or a vector with the gene encoding the *Xa7-*inducing effector (*avrXa7*) at normal and high-temperature at 72 hpi. Scale is indicated by the black bar. (b) Box plots of log_10_ transformed bacterial quantity of rice leaves inoculated with *Xo* X11-5A *talΔCRR* and *Xo* X11-5A *avrXa7* at normal and high temperature. One-way ANOVA revealed differences among treatments within all time points except 0 hpi (p < 0.0005). Within time points, letters indicate differences as determine by two-tailed pairwise t-test (FDR-adjusted p-value < 0.05).

Plants of rice line IRBB61, which carries the bacterial blight resistance gene *Xa7*, were grown to maturity under normal conditions (29/23°C day/night) in a growth chamber. Half of these plants were moved to a growth chamber set to emulate moderate heat stress (35/29°C day/night; hereafter referred to as high temperature). These plants were inoculated with *Xanthomonas oryzae* (*Xo*) strain X11-5A, a generally low virulence strain of *Xo* [29], carrying either an empty plasmid vector or a vector encoding AvrXa7, the *Xa7*-mediated resistance inducing protein (Table 1). Plants in the susceptible interaction showed chlorosis, with a stronger yellowing in the high temperature plants (Fig 1A). During the resistant interactions, plants showed a browning indicative of the hypersensitive response associated with resistance to *Xo* harboring *avrXa7*, with a stronger response at high temperature. Plants at high temperature in the resistant interaction also showed reduced bacterial numbers due to *Xa7*-mediated defense as early as 12 h post-inoculation (hpi), while plants at normal temperature showed reduced bacterial numbers by 24 hpi (Fig 1B). By 48 hpi, during the resistant interaction, the plants at high temperature showed greatly reduced bacterial numbers when compared to the plants at normal temperature. During the susceptible interaction, bacterial numbers showed no differences due to high temperature. These observations confirmed that the *Xa7-*mediated resistance was stronger and faster at high temperature than at low temperature.

**Table 1:** Experimental design for transcriptomics experiment involving rice undergoing heat/*Xo* stresses.

Host Plant	Temperature Regime	Pathogen	Plant Response
IRBB61 rice	Normal	*Xo* X11-5A empty vector	Susceptible
IRBB61 rice	High	*Xo* X11-5A empty vector	Susceptible
IRBB61 rice	Normal	*Xo* X11-5A *avrXa7*	Resistant
IRBB61 rice	High	*Xo* X11-5A *avrXa7*	More resistant

### Acclimation to high temperature alters the rice transcriptome

**Fig 2:**
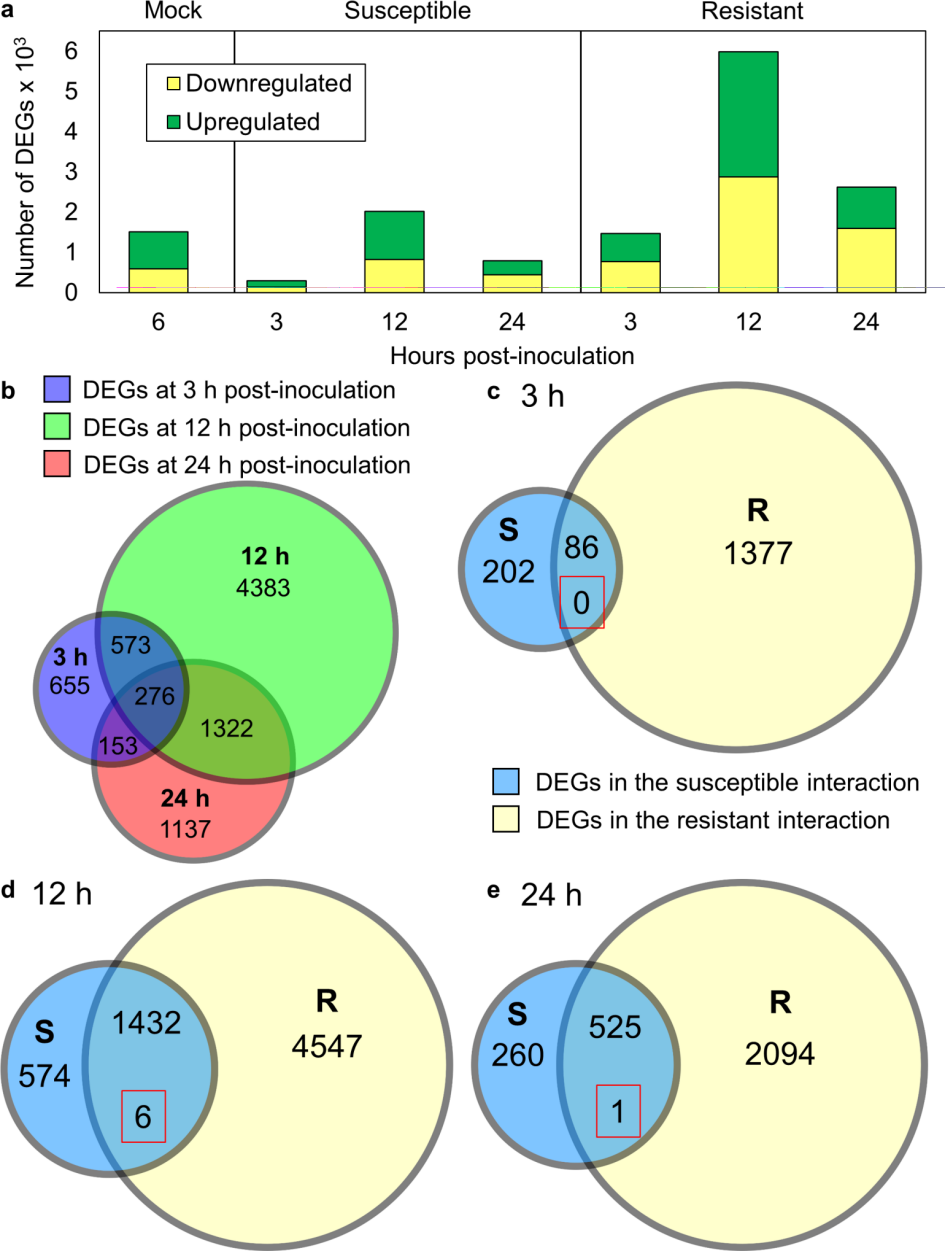
Differential gene expression analysis. (a) Genes differentially up and down-regulated at high relative to normal temperature in mock-inoculated plants, and during susceptible and resistant interactions. (b) Number of DEGs per time point, with DEGs from the susceptible and resistant interactions combined per each time point. (c–e) Number of DEGs up or down-regulated by high temperature in plants in the susceptible (S) plants or resistant (R) interaction at (c) 3 h, (d) 12 h, and (e) 24 hpi. The red-squared number represents DEGs which were oppositely regulated by susceptibility/resistance.

**Fig 3:**
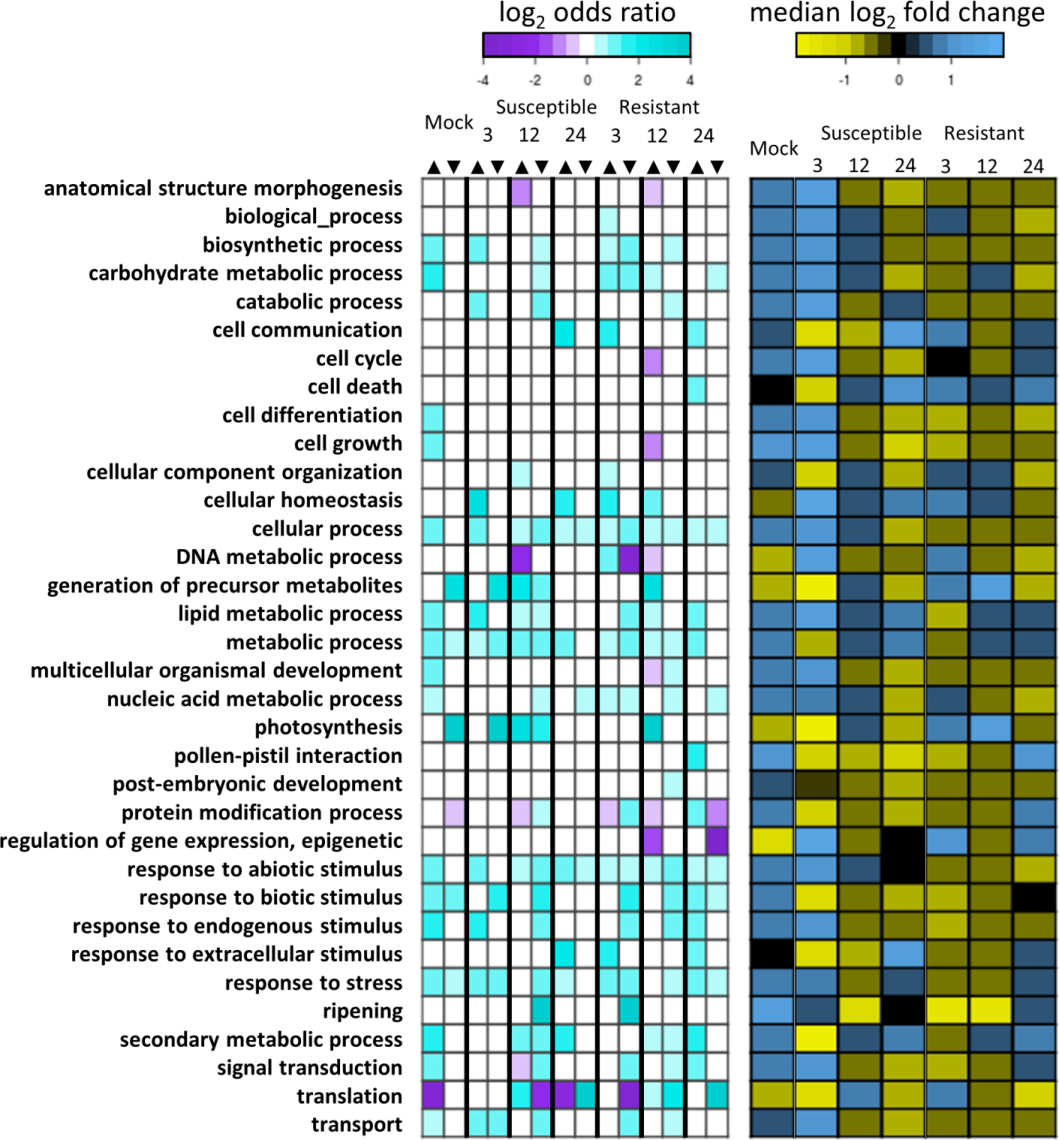
GO term analysis. Left panel shows the log_2_ odds ratio of genes regulated by high temperature with the GO annotation to genes not regulated by high temperature with the GO annotation. Positive value indicates the term is over-represented in regulated genes, while negative value indicates the term is under-represented in regulated genes. Enrichment analysis was conducted for each term, in each treatment condition (Fisher’s exact test, FDR-corrected p-value < 0.05). The arrows indicate the genes were either up-regulated or down-regulated by high temperature treatment. Terms not statistically enriched are shown as white/zero log_2_ odds ratio. Right panel shows the median log_2_ fold change per term. Positive value indicates more genes annotated with the term are up-regulated, while negative value indicates more genes annotated with the term are down-regulated.

To address the impact of high temperature acclimation on the rice transcriptome, we conducted an RNA-seq experiment using leaves from mock-inoculated plants grown in normal and high temperature conditions as described above (see S1 Table for next generation summary statistics). Differential gene expression was conducted, with genes having FDR-corrected p-values of < = 0.01 considered differentially expressed; this analysis revealed 1,511 differentially expressed genes (DEGs), with the majority of DEGs being up-regulated by high temperature (Fig 2A). Exposure of mock-treated plants to high temperature led to the upregulation of genes involved in many annotated biological processes (Fig 3; Tables A and B in S2 Table). Stress-responsive gene ontology (GO) terms were enriched and over-represented in genes up-regulated by high temperature, including the following terms: ‘response to stress’, ‘response to abiotic stimulus’, ‘response to biotic stimulus’, and ‘response to endogenous stimulus’. The GO terms ‘response to stress’ and ‘response to biotic stimulus’ were also over-represented in genes down-regulated by high temperature, but the median log_2_ fold change for genes annotated with these terms was positive, indicating that there were more genes with these annotations being up-regulated than down-regulated. GO terms associated with metabolic processes were enriched in genes up-regulated by high temperatures, including biosynthetic, carbohydrate metabolic, catabolic, lipid metabolic, and secondary metabolic processes. The DEGs associated with these metabolic terms had positive median log_2_ fold change, indicating that metabolic processes were generally up-regulated in mock-treated plants at high temperature. DEGs associated with energy metabolism terms, including generation of precursor metabolites and energy and photosynthesis, were enriched in genes down-regulated by high temperature and had negative log_2_ fold changes, indicating that energy metabolism was generally down-regulated at high temperature. Enriched GO terms for cellular processes included ‘signal transduction’, ‘transport’, ‘cell differentiation’, ‘cell death’, and ‘cell growth’. DEGs for all of these processes were generally more up-regulated in mock-treated plants at high temperature. The GO term ‘translation’ was under-represented in genes up-regulated by high temperature with a negative median log_2_ fold change, indicating that gene translation was down-regulated by high temperature. To confirm that this transcriptomic response was due to temperature and not caused by a temperature-dependent wound response from the inoculation method, expression of VSP2, a gene responsive to a mediator of wound responses (jasmonic acid), was measured via qRT-PCR. Expression of VSP2 was not significantly changed by high temperature at 3, 6, and 12 h post-mock inoculation (S1 Fig). In addition, from a panel of 100 rice wound response genes in our RNA-Seq data, 96 were not differentially expressed at high temperature (S3 Table). Thus, plants acclimated to high temperature stress have dramatically altered transcriptomic profiles when compared to plants at normal temperature, and wound response from inoculation is not exacerbated at high temperature.

### Plants respond uniquely to temperature in the resistant and susceptible interactions

To assess the plant transcriptomic response to high temperature during the susceptible and resistant interactions, gene expression profiles were determined from plants inoculated with the same strains as previously described at 3, 12, and 24 hpi (Table 1, S1 Table). The transcriptome of all pathogen-treated plants was altered at high temperature relative to normal temperature, but plant transcriptomes in the resistant interaction showed more differentially expressed genes (Fig 2A). A total of 8,499 DEGs were differentially regulated by high temperature in all biotic treatment conditions. While there was some overlap in DEGs per time point, the majority of these DEGs were unique to a single time point (Fig 2B), indicating that time was the strongest factor influencing transcriptome response. Within each time point, there were shared and unique transcriptomic responses in both the resistant and susceptible interactions (Fig 2C–2E). Most shared DEGs were similarly regulated between both interactions, with only a few genes oppositely regulated based on pathogen treatment. At all time points, the number of DEGs unique to the resistant interaction was roughly one order of magnitude greater than the number of DEGs unique to the susceptible interaction. This indicated that while exposure to high temperature caused similar transcriptome responses in both the susceptible and resistant interactions, more changes were observed in the resistant interaction, and most of these were unique to that response.

In general, biological processes were up-regulated in the susceptible interaction and down-regulated in the resistant interaction (Fig 3; Tables C-N in S2 Table). Genes annotated with the GO term ‘response to abiotic stimulus’ showed opposite trends in the susceptible interaction; while genes annotated with this term were generally up-regulated in the susceptible interaction, they were generally down-regulated in the resistant interaction. Surprisingly, genes annotated with ‘response to biotic stimulus’ were generally down-regulated by high temperature in both the susceptible and resistant interactions. Genes annotated with ‘response to stress’ were up-regulated in the susceptible interaction at 3 and 24 hpi, and down-regulated in the resistant interaction at 3 and 12 hpi. Genes annotated with the GO terms ‘biosynthetic process’, ‘carbohydrate metabolic process’, and ‘cellular process’ showed similar trends, being generally up-regulated by high temperature in the susceptible interaction and down-regulated by high temperature in the resistant interaction, while genes annotated with the GO terms ‘metabolic process’ and ‘lipid metabolic process’ showed the opposite trend. Regulation of genes associated with GO terms in plants in the susceptible interaction responded to high temperature in a way similar to the mock-treated plants, while these processes were generally oppositely regulated in plants in the resistant interaction. This suggested that the plants undergoing *Xa7-*mediated resistance at high temperature were responding to high temperature by regulating many biological processes in a way opposite to both uninoculated plants and the plants in the susceptible interaction.

### Rice plants experiencing heat stress alter hormone synthesis and downstream signaling

**Fig 4:**
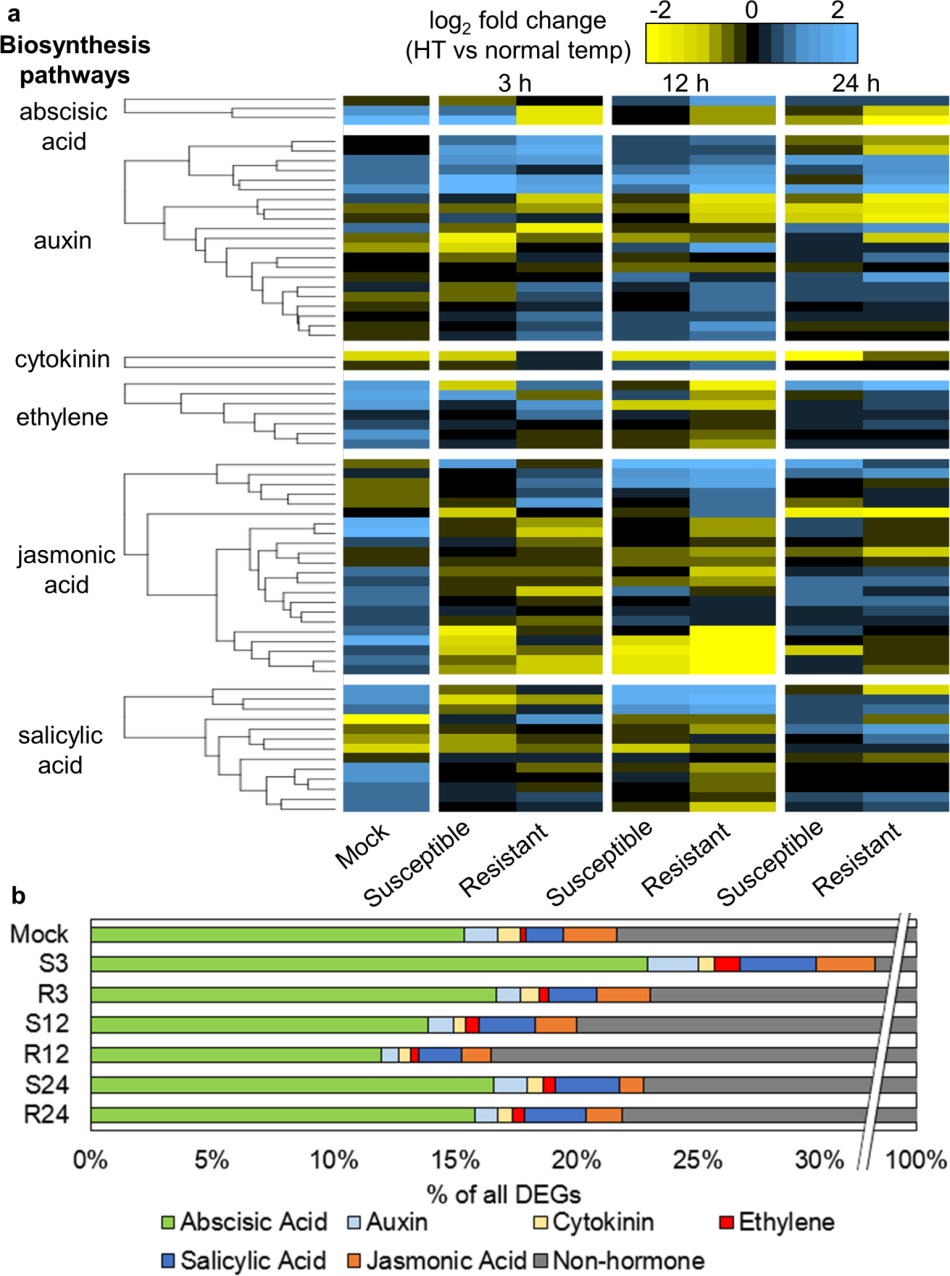
Differential expression of upstream and downstream hormone genes at high temperature. (a) Fold change for hormone biosynthesis genes at high temperature relative to normal temperature is represented for mock-inoculated plants, and plants during susceptible and resistant interactions. Hormone biosynthesis genes were selected for display only if they were differentially expressed in at least one column. (b) Downstream hormone-responsive genes represented as proportions of total DEGs for mock-inoculated plants, and plants during susceptible and resistant interactions.

Hormones are key regulators of plant responses to both biotic and abiotic stresses [21, 22]. Many transcripts encoding genes directing phytohormone biosynthesis were in the set of all DEGs. All hormone biosynthesis gene families were differentially expressed in response to high temperature, in all mock and pathogen treatments, suggesting that plants experiencing high temperature stress fundamentally alter endogenous hormone balance. There was also considerable overlap in hormone biosynthesis DEGs in the susceptible and resistant interaction, counter to the noted earlier trend of mostly unique transcriptomic responses (Fig 4A, S4 Table). The expression patterns of hormone biosynthesis genes in the mock-treated plants most closely resembled that of plants in the susceptible interaction at all time points, especially in the ABA, auxin, and cytokinin biosynthesis pathways (Fig 4A). Genes involved in ABA biosynthesis were strongly up-regulated at high temperature in both mock-treated plants and plants during the susceptible interaction at 3 hpi, and strongly down-regulated in plants undergoing resistance responses at all time points. Genes predicted to contribute to biosynthesis of salicylic acid (SA), a pathogen-responsive hormone important in defense responses, were regulated independent of biotic treatment–being up-regulated by high temperature in the mock treatment, down-regulated by high temperature in both biotic interactions at 3 and 12 hpi, and up-regulated by high temperature in both biotic interactions at 24 hpi. This trend suggests that during resistant interactions at high temperature, plants enact transcriptional control of hormone metabolism that is unique from uninoculated plants in response to high temperature, while plants progressing towards a diseased state closely resemble uninoculated plants.

To further address the role of plant hormones in this response, analysis was conducted to examine how known hormone-responsive genes behave during simultaneous pathogen and temperature stresses. Many hormone-responsive DEGs were perturbed at high temperature in all mock- and pathogen-treated plants (Fig 4B, S5 Table). ABA-responsive genes made up the largest proportion of hormone-responsive DEGs in all treatments. At each time point, a larger number of DEGs, but a smaller proportion of total DEGs, in the resistant interaction were hormone-responsive relative to the susceptible interaction at the same time point. Regardless of pathogen treatment, rice plants greatly altered hormone-regulated genes and downstream signaling in response to high temperature.

### Rice plants expressing *Xa7-*mediated resistance suppress expression of ABA-responsive genes at high temperature

**Fig 5:**
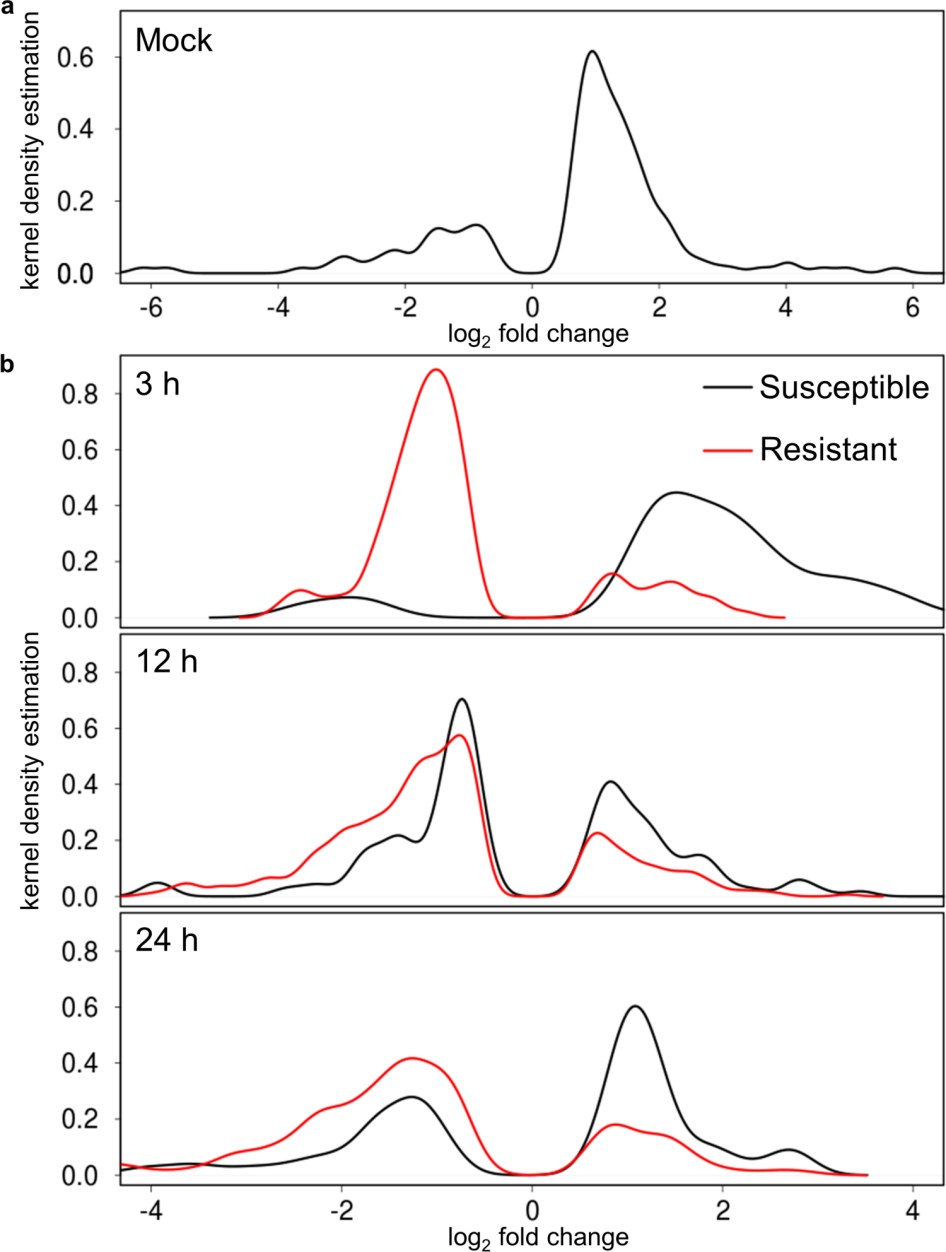
Differential expression of ABA up-regulated genes at high temperature. (a) Kernel density estimate of log_2_ fold change for ABA up-regulated genes differentially regulated in mock-inoculated plants. (b) Kernel density estimates of log_2_ fold change for ABA up-regulated genes differentially regulated in plants during susceptible and resistant interactions at 3, 12, and 24 hpi.

**Fig 6:**
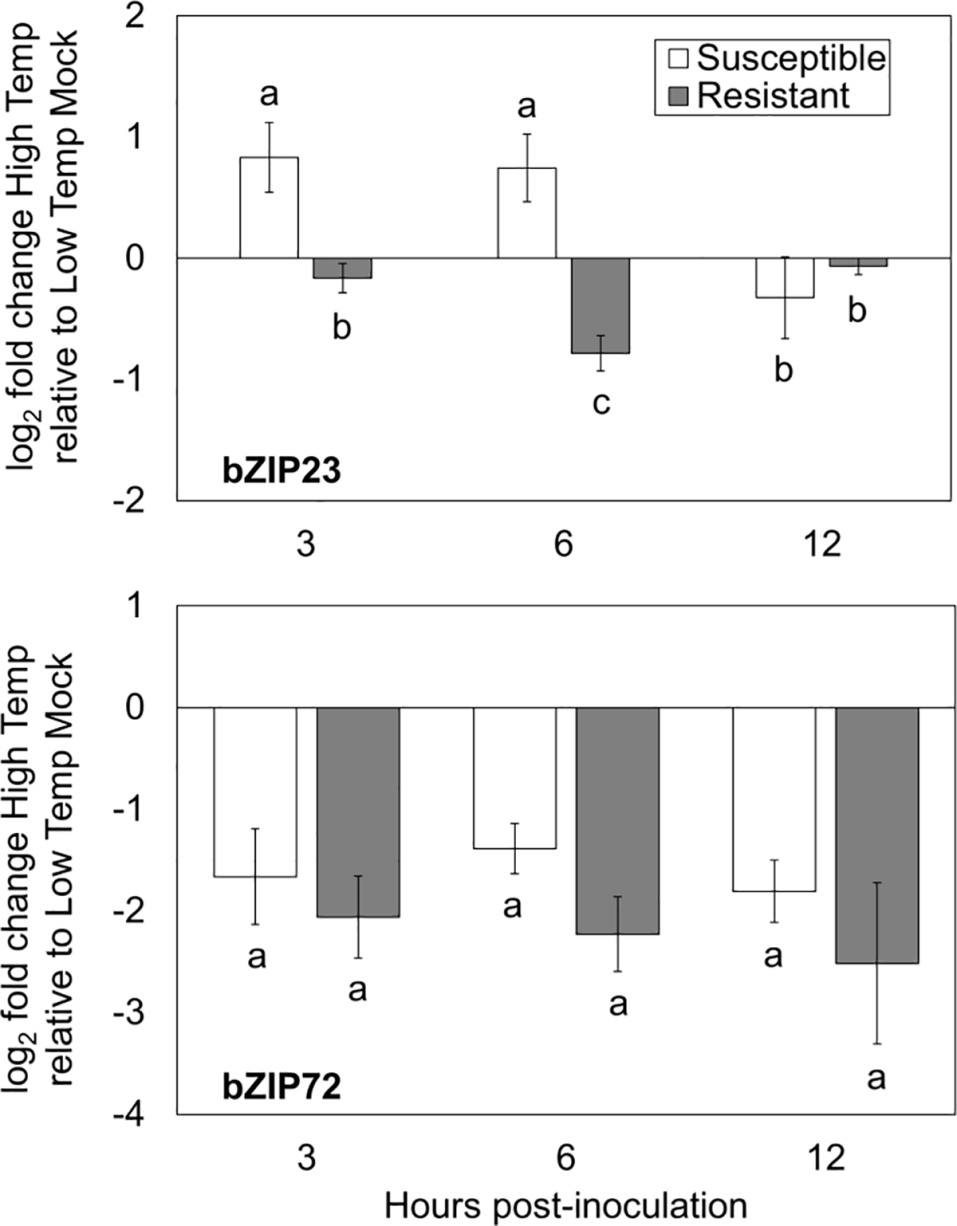
Analysis of ABA marker gene expression. Bars represent mean log_2_ fold changes of ABA marker genes (a) bZIP23 and (b) bZIP72 in plants during susceptible and resistant interactions at high temperature relative to the normal temperature mock-inoculated control at each time point as measured by qRT-PCR. Error bars represent standard error of the mean, with n = 8 and 4 for (a) and (b) respectively, and letters indicate pairwise groupings (pairwise t test, p < 0.05).

The fold changes of known ABA-up-regulated genes, identified as being induced 2-fold or greater following ABA treatment by a previous microarray study [30], were inspected to give insight into the associated regulatory trends. ABA-up-regulated genes were mostly up-regulated at high temperatures in the mock-treated plants (Fig 5A, S6 Table). The transcriptome of plants in the susceptible interaction showed the same trend at 3 and 24 hpi, with the opposite trend at 12 hpi (Fig 5B, S6 Table). In the resistant interaction, ABA-up-regulated genes were down-regulated at all time points, suggesting that during resistance, plants suppressed ABA downstream responses. Expression of the ABA-responsive master regulators bZIP23 and bZIP72 was tested using quantitative reverse transcriptase PCR. In susceptible plants at high temperature, expression of bZIP23 was increased approximately two-fold compared to the low temperature, mock-inoculated control at 3 and 6 hpi, while expression was reduced two-fold in resistant plants at 6 hpi (Fig 6A). Interestingly, while bZIP72 was suppressed by high temperature in the resistant interaction, it was also suppressed during the susceptible interaction (Fig 6B). In agreement with the findings for SA biosynthetic genes, genes responsive to SA were up-regulated by high temperature in the mock-inoculated plants (Fig A in S2 Fig, S7 Table). Conversely, SA-responsive genes were down-regulated by high temperature at 3 and 12 hpi in the susceptible interaction, and at all time points in the resistant interaction (Fig B in S2 Fig, S7 Table). These trends indicate that during high temperature stress, rice plants undergo significant changes in not just ABA-responsive gene expression but in the regulatory networks that drive ABA-responsive gene expression, and that enhanced *Xa7-*mediated resistance at high temperature is likely independent of SA.

### An ABA responsive element-like motif was enriched in the promoters of DEGs

**Fig 7:**
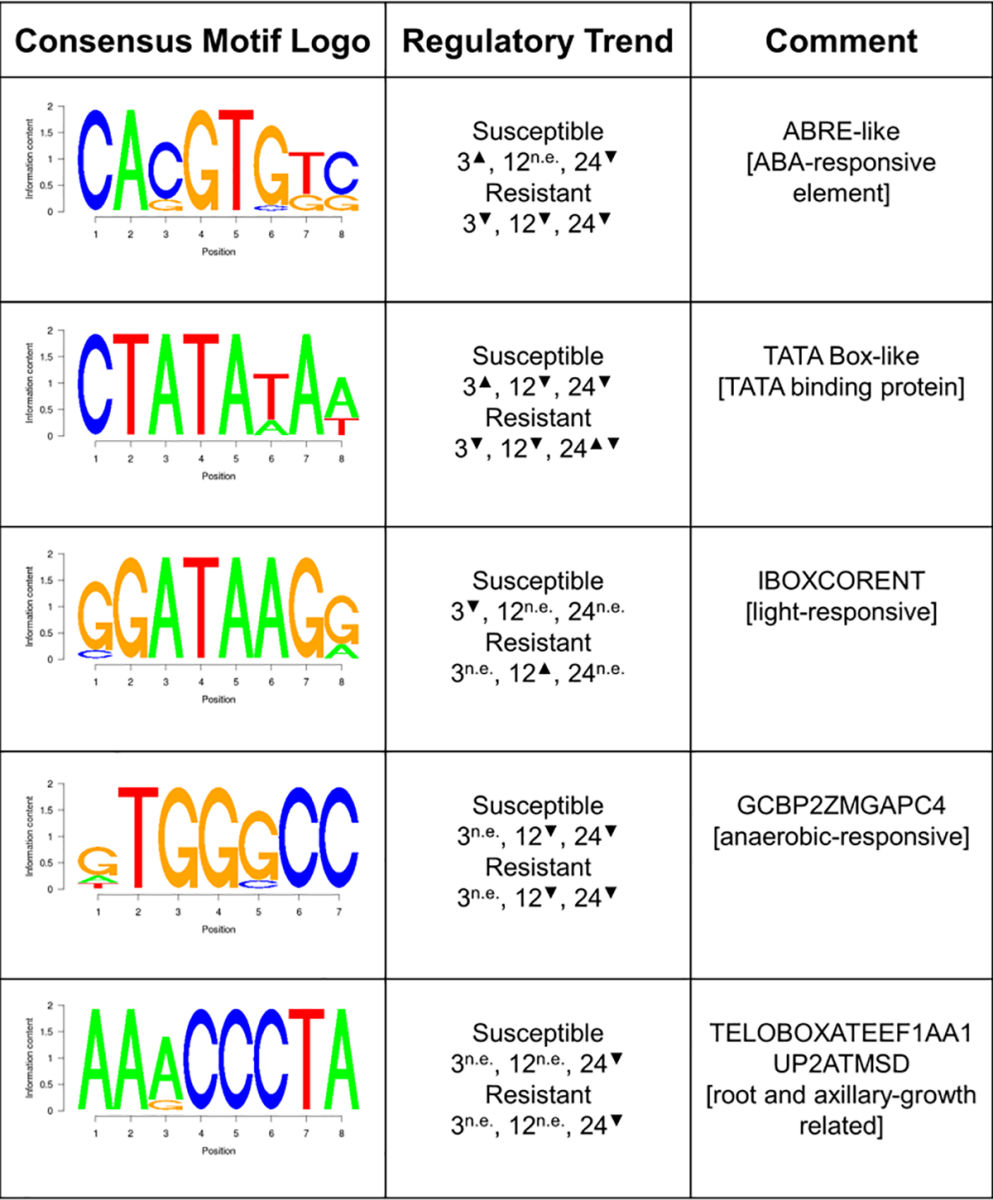
Analysis of *cis-*regulatory element enrichment in DEGs. Up arrows indicate the motifs are enriched in up-regulated DEGs in the given time point, down arrows indicate enrichment in down-regulated DEG, and *n*.*e*. indicates no significant enrichment as determined by Fisher’s exact test (p < 0.05). Similarity to motifs in PLACE database [32] is indicated.

Motif analysis was conducted on the upstream promoter sequences of DEGs for discovery of *cis-*regulatory elements that might give insight into the observed gene expression patterns. A motif was identified that resembled the ABA responsive element (ABRE), a G-box family motif recognized by bZIP family transcription factors that is found in the promoters of many ABA responsive genes [31]. This ABRE-like element was enriched in the promoters of genes up-regulated in the susceptible interaction at high temperature at 3 hpi, genes down-regulated in the susceptible interaction at 24 hpi, and genes down-regulated in the resistant interaction at all time points (Fig 7; S8 Table). Several other motifs identified from the Plant *cis-*acting Regulatory DNA Elements database [32] were also enriched in the DEGs, including motifs resembling the TATA box, the light-responsive IBOXCORENT, the anaerobic-responsive GCBP2ZMGAPC4, the root growth-related TELOBOXATEEF1AA1, and the axillary growth-related UP2ATMSD (Fig 7; S8 Table). The enrichment trends of these motifs may give insight into the rice processes perturbed by high temperature stress over the course of a 24 h day. Most importantly, the trends observed in the enrichment of ABRE-like motifs are evidence that plants activated the ABA response at high temperature early during the susceptible interaction, and suppressed the ABA response at high temperature during the resistant interaction.

## Discussion

During periods of high temperature stress, *Xa7-*mediated rice resistance to *Xo* is enhanced, while resistance regulated by other *R* genes is generally repressed [15], but the underlying cause of this phenomenon is heretofore not understood. To provide insights into how *Xa7-*mediated resistance is enhanced at high temperature, we conducted a transcriptomics experiment with RNA-Seq technology to identify the transcriptomic changes in rice during *Xa7-*mediated resistance at high temperature. A set of 8,499 DEGs was identified as temperature responsive in one rice cultivar, IRBB61, experiencing both a susceptible interaction with *Xo* strain X11-5A and a resistant interaction with *Xo* strain X11-5A carrying a plasmid encoding the *Xa7*-inducing effector protein AvrXa7 across three time points.

Under all treatments, expression of genes involved in metabolic processes was altered by high temperature. Genes annotated with GO terms related to metabolism were generally up-regulated by high temperature in plants treated with mock inoculation and in the susceptible interaction, while these genes were generally down-regulated in the resistant interaction. However, genes annotated with the GO term ‘photosynthesis’ showed the opposite trend. Photosynthesis is generally inhibited during high temperature stress, and reduced primary metabolism is associated with thermotolerance in plants [33, 34]. Reduced photosynthesis is also associated with pathogen attack in both susceptible and resistant interactions [35]. It is therefore surprising that enhanced *Xa7-*mediated resistance at high temperature is associated with the upregulation of photosynthesis-related genes. Additional experimentation is needed to explore the dynamics of primary metabolism at high temperature during *Xa7*-mediated resistance.

Genes in the ABA pathway were notably perturbed by high temperature during biotic stresses. ABA biosynthesis and ABA-responsive genes were induced by high temperature in both mock-treated plants and plants in the susceptible interaction at 3 and 24 hpi, and suppressed by high temperature in plants in the resistant interaction at all time points. ABA-responsive genes were down-regulated in the susceptible interaction at 12 hpi, possibly due to diurnal effects. SA-responsive genes were also induced by high temperature in mock-treated plants, but generally repressed by high temperature in all biotic interactions. This trend suggests that regulatory differences in ABA responsiveness are important to the rice resistance phenotype during a plant’s response to simultaneous heat stress and *Xo* attack, and raises the interesting hypothesis that enhanced *Xa7-*mediated resistance at high temperature is independent of SA.

ABA is a developmental plant hormone that is active in triggering plant physiological changes for acclimation to abiotic stresses such as drought, cold, heat, and salt stresses [36–39]. ABA also plays a complex role during plant response to biotic stresses. For example, ABA signaling can lead to closure of stomata, a common entry point for plant pathogens [40]. However, ABA generally plays a negative role in plant defense responses to biotic stresses through antagonistic interactions with defense response pathways. In Arabidopsis, ABA treatment suppresses the induction of both systemic acquired resistance, a plant immune response effective against a broad range of pathogens, and the hypersensitive response [7, 41]. In light of these previous studies, our results suggest that suppression of ABA response is vital for the hypersensitive response associated with *Xa7-*mediated resistance.

In rice, ABA interacts antagonistically with the defense response hormone SA, leading to reduced resistance to blast disease and increased bacterial blight disease severity [27, 28]. However, our results suggest that ABA and SA were regulated independently instead of antagonistically. While another study also showed that ABA enhanced rice susceptibility to *Xo* by antagonizing SA, when plants were treated with the ABA biosynthesis inhibitor fluridone, the resulting resistance to the pathogen was independent of SA [24]. This suggests that there is some SA-independent mechanism of resistance to *Xo* upon depletion of ABA, which might explain why genes annotated with the GO term ‘response to biotic stress’ were down-regulated in the resistant interaction in this study. In agreement with our findings, ABRE motifs have previously been identified in the promoters of *Xo-*responsive genes in rice [42]. Interestingly, exogenous ABA has been linked to enhanced swimming ability in *Xo* [25], suggesting that the *Xo-*rice interaction has been evolutionarily shaped by ABA. The results presented in our study further indicate that ABA response and plant defense are inversely regulated.

In addition to functioning better at high temperature, *Xa7* is also more effective during drought stress [17]. The plant transcriptional responses to drought and heat stress are drastically different, with many distinct genes triggered by each stress [43, 44], but a regulatory mechanism shared by both stresses is the accumulation of ABA. It is therefore tempting to speculate that *Xa7* activity directly represses ABA biosynthesis, signaling, or both. In fact, the ABRE-like motif identified in this study might serve as a binding element for a transcriptional repressor during defense. If this turns out to be the case, this could inform rice breeders on selection strategies for enhancing disease resistance at high temperature; for example, promoter regions for susceptibility and resistance genes could be screened for this motif across multiple varieties. However, further experimentation is needed to conclusively show whether the repression of ABA response is actively triggered during *Xa7*-mediated resistance or if it is a side effect of resistance to *Xo*. Additional work is needed to explore whether downregulation of abiotic response by *Xa7-*mediated resistance impacts heat tolerance. In a natural interaction between rice and *Xo* pathovar *oryzae*, the bacterial blight pathogen, the pathogen proliferates within the rice xylem, limiting water availability to rice leaf cells. The reduction of ABA signaling in resistant plants at high temperature may therefore be due in part to the reduced water stress associated with the limitation on bacterial proliferation induced by *Xa7*. Further studies are necessary to tease apart the role of the ABA signaling pathway to *Xa7-*mediated resistance.

## Conclusions

This study presents novel results of a transcriptomic analysis of rice during simultaneous heat stress and *Xo* infection, with plant responses during both susceptible and resistant interactions. The results revealed that the ABA pathway was activated during both high temperature stress and the susceptible interaction at high temperature, and was repressed during *Xa7*-mediated defense at high temperature. The SA pathway was also down-regulated at high temperature in both the susceptible and resistant interactions, suggesting that enhanced *Xa7-*mediated resistance is likely independent of SA signaling. A novel sequence motif that was similar to the ABRE was identified in the promoters of genes up-regulated by high temperature during the susceptible interaction and down-regulated by high temperature during the resistant interaction. These results suggest that ABA is an important node for cross-talk between plant transcriptional response pathways to high temperature stress and pathogen attack. This pathway represents an important area of study for future research in understanding how plants deal with combined abiotic and biotic stresses.

## Methods

### Plant materials and growth conditions

Seeds of rice NIL IRBB61 (Vera Cruz CM *et al*. unpublished) were germinated on wet filter paper under constant light at 28°C. After emergence, the seedlings were transplanted in soil in a greenhouse (approximately 23°/18°C day/night, 75–85% relative humidity) and grown for three weeks. The plants were evenly distributed to two growth chambers set to normal (29°/23°C day/night) and high (35°/29°C day/night) temperature regimes and 85% relative humidity. Plants were acclimated to growth chambers for one week before inoculations.

### Bacterial strains, inoculations, and bacterial quantification

Cultures of *Xo* strain X11-5A carrying pKEB31 plasmids containing ORFs for *avrXa7* and *talΔCRR* (a non-functional TAL effector lacking the DNA binding region), described in [45] and [46] respectively, were grown at 28°C on peptone sucrose agar (PSA) [47] with 2 ug/mL tetracycline overnight and diluted in sterile distilled water to 10^8^ cfu/mL. Plant leaves were inoculated with dilutions of both strains and water (for mock) using a needleless syringe [48]. Leaves designated for RNA extractions and bacterial quantification were inoculated along a 4 cm section with six infiltration sites, while leaves designated for symptom observation were inoculated along an approximately 10 cm section with four infiltration sites. Inoculations were conducted approximately 3 h after growth chamber lights reached full intensity in the morning. Tissue was collected at full light (3 h, 24 h) and full dark (12 h) light stages. For bacterial quantification, inoculated leaf tissue was surface sterilized with 10% bleach and rinsed three times with sterile water, then ground in 1mL of sterile water in a tissue macerator (Qiagen TissueLyser II). The extract was plated in a dilution series on PSA with 2 ug/mL tetracycline and incubated overnight at 28°C. Pairwise analysis of bacterial numbers was performed in R [49].

### RNA extraction, sequencing, and qRT-PCR

Total RNA was extracted from plant tissue at the site of inoculation using a Sigma Aldrich Spectrum Plant Total RNA Kit as per kit instructions. RNA was collected for two biological replicates for each condition. RNA from mock-treated leaves was submitted to the University of North Carolina High-Throughput Sequencing Facility for cDNA generation via TruSeq RNA library construction kits with multiplex adapter primers and single-end 50 bp sequencing via Illumina HiSeq 2500. RNA from pathogen-treated leaves was submitted to Michigan State University Genomics Core for TruSeq mRNA library preparation with multiplex barcodes and sequencing via Illumina HiSeq 50 bp single read sequencing. For qRT-PCR, cDNA was generated from the previously collected RNA using Quantabio qScript cDNA SuperMix kit. Primers and thermal cycler conditions for qRT-PCR follow [50] for bZIP23, bZIP72 and [51] for VSP2. Data was analyzed using the ΔΔCT method [52].

### Gene expression analyses

Sequence reads were processed with FASTX Toolkit 0.0.13 [53] to remove low quality reads. The high-quality reads were aligned to the MSU RGAP 7.0 rice reference genome [54] using TopHat 2.1.1 [55] and counted using HTSeq 0.6.1 [56]. Sequence reads and gene counts are available in the Gene Expression Omnibus repository under accession number GSE95668. Differential gene expression analyses were conducted using the Bioconductor package edgeR [57, 58]. Genes were considered differentially expressed in a condition if FDR-corrected p-value was less than or equal to 0.01. Rice wound response genes were identified from publicly available microarray data (NCBI Gene Expression Omnibus Accession GSE77097). The top 100 genes were chosen from this data by fold change. Fisher’s exact test was used for GO term enrichment analysis. A GO term was considered statistically significant if FDR-corrected p-value was < = 0.05. Heatmaps were prepared using the heatmap.2 function from the R package gplots [59]. Hierarchical clustering of hormone biosynthesis genes was performed with the hclust R function using the WPGMA method [49]. Hormone-responsive genes used in analysis were identified from a microarray study [30]. Kernel density estimates were prepared with the density function from the R core package [49]. DREME [60] was used for motif discovery with the 1000 bp sequences upstream of putative transcription start sites from the reference genome. STAMP [61] was used for DNA motif matching.

## Supporting information

S1 Fig Mean log_2_ fold change of VSP2 in mock treated plants at high temperature as measured by qRT-PCR. There were no significant differences in expression of the wound-responsive jasmonic acid marker VSP2 at 3, 6, and 12 h post-mock inoculation at high temperature relative to low temperature (Student’s t-test, p > 0.05). Error bars represent SEM (n = 6).
(PNG)

S2 Fig Differential expression of SA up-regulated genes at high temperature. (a) Kernel density estimate of log_2_ fold change for SA up-regulated genes differentially regulated by high temperature in mock inoculated plants. (b) Kernel density estimates of log_2_ fold change for SA up-regulated genes differentially regulated by high temperature in plants during susceptible and resistant interactions at 3, 12, and 24 hpi.
(PNG)

S1 Table Sequencing reads and mapping summary statistics. (DOCX)

S2 Table GO term enrichment analysis for genes differentially expressed by high temperature. (DOCX)

S3 Table Differential expression of rice wound response genes from NCBI GEO Accession GSE77097. (DOCX)

S4 Table Hormone biosynthesis genes that were differentially expressed due to high temperature in at least one treatment/time point. (DOCX)

S5 Table Hormone response genes differentially expressed at high temperature. (DOCX)

S6 Table Differential expression analysis of ABA-responsive genes. (DOCX)

S7 Table Differential expression analysis of SA-responsive genes. (DOCX)

S8 Table Odds ratios of promoter motifs in the promoters of different gene sets. (DOCX)

## References

[1] Oerke E-C. Crop losses to pests. J Agric Sci. 2006;144:31–43.

[2] Scholthof K-BG. The disease triangle: pathogens, the environment and society. Nat Rev Microbiol. 2007;5(2):152–6. doi: 10.1038/nrmicro1596 17191075 10.1038/nrmicro1596

[3] Madgwick JW, West JS, White RP, Semenov MA, Townsend JA, Turner JA et al Impacts of climate change on wheat anthesis and fusarium ear blight in the UK. Eur J Plant Pathol. 2011;130:117–31.

[4] Mohr PG, Cahill DM. Abscisic acid influences the susceptibility of *Arabidopsis thaliana* to *Pseudomonas syringae* pv. *tomato* and *Peronospora parasitica*. Funct Plant Biol. 2003;30:461–9. 10.1071/FP02231 32689031

[5] O'Hara NB, Rest JS, Franks SJ. Increased susceptibility to fungal disease accompanies adaptation to drought in *Brassica rapa*. Evolution. 2016;70(1):241–8. doi: 10.1111/evo.12833 26648585 10.1111/evo.12833 PMC4715916

[6] Prasch CM, Sonnewald U. Simultaneous application of heat, drought, and virus to Arabidopsis plants reveals significant shifts in signaling networks. Plant Physiol. 2013;162(4):1849–66. doi: 10.1104/pp.113.221044 23753177 10.1104/pp.113.221044 PMC3729766

[7] Yasuda M, Ishikawa A, Jikumaru Y, Seki M, Umezawa T, Asami T et al Antagonistic interaction between systemic acquired resistance and the abscisic acid–mediated abiotic stress response in *Arabidopsis*. Plant Cell. 2008;20(6):1678–92. doi: 10.1105/tpc.107.054296 18586869 10.1105/tpc.107.054296 PMC2483369

[8] Whitham S, McCormick S, Baker B. The *N* gene of tobacco confers resistance to tobacco mosaic virus in transgenic tomato. Proc Natl Acad Sci USA. 1996;93(16):8776–81. 8710948 10.1073/pnas.93.16.8776 PMC38750

[9] de Jong CF, Takken FL, Cai X, de Wit PJ, Joosten MH. Attenuation of Cf-mediated defense responses at elevated temperatures correlates with a decrease in elicitor-binding sites. Mol Plant-Microbe Interact. 2002;15(10):1040–9. doi: 10.1094/MPMI.2002.15.10.1040 12437302 10.1094/MPMI.2002.15.10.1040

[10] Zhu Y, Qian W, Hua J. Temperature modulates plant defense responses through NB-LRR proteins. PLoS Pathog. 2010;6(4):e1000844 doi: 10.1371/journal.ppat.1000844 20368979 10.1371/journal.ppat.1000844 PMC2848567

[11] Zhao F, Li Y, Chen L, Zhu L, Ren H, Lin H et al Temperature dependent defence of *Nicotiana tabacum* against *Cucumber mosaic* virus and recovery occurs with the formation of dark green islands. J Plant Biol. 2016;59(3):293–301.

[12] Li W, Xu Y-P, Cai X-Z. Transcriptional and posttranscriptional regulation of the tomato leaf mould disease resistance gene *Cf-9*. Biochem Biophys Res Commun. 2016;470(1):163–7. doi: 10.1016/j.bbrc.2016.01.015 26768363 10.1016/j.bbrc.2016.01.015

[13] Reddy A, Mackenzie D, Rouse D, Rao A. Relationship of bacterial leaf blight severity to grain yield of rice. Phytopathology. 1979;69(9):967–9.

[14] Suh J-P, Jeung J-U, Noh T-H, Cho Y-C, Park S-H, Park H-S et al Development of breeding lines with three pyramided resistance genes that confer broad-spectrum bacterial blight resistance and their molecular analysis in rice. Rice. 2013;6(1):5 doi: 10.1186/1939-8433-6-5 24280417 10.1186/1939-8433-6-5 PMC4883717

[15] Webb KM, Ona I, Bai J, Garrett K, Mew T, Cruz V et al A benefit of high temperature: increased effectiveness of a rice bacterial blight disease resistance gene. New Phytol. 2010;185(2):568–76. doi: 10.1111/j.1469-8137.2009.03076.x 19878463 10.1111/j.1469-8137.2009.03076.x

[16] Hopkins CM, White FF, Choi SH, Guo A, Leach JE. Identification of a family of avirulence genes from *Xanthomonas oryzae* pv. *oryzae*. Mol Plant-Microbe Interact. 1992;5:451–9. 1335800 10.1094/mpmi-5-451

[17] Dossa GS, Torres R, Henry A, Oliva R, Maiss E, Cruz CV et al Rice response to simultaneous bacterial blight and drought stress during compatible and incompatible interactions. Eur J Plant Pathol. 2017;147:115–27.

[18] Dossa GS, Henry A, Oliva R, Maiss E, Kumar A, Cruz CV et al Combining drought QTLs and bacterial blight Xa-genes to control bacterial blight disease under drought stress. Agric Ecosyst Environ. 2016;233:282–90.

[19] Cruz CMV, Bai J, Oña I, Leung H, Nelson RJ, Mew T-W et al Predicting durability of a disease resistance gene based on an assessment of the fitness loss and epidemiological consequences of avirulence gene mutation. Proc Natl Acad Sci USA. 2000;97(25):13500–5. doi: 10.1073/pnas.250271997 11095723 10.1073/pnas.250271997 PMC17604

[20] Verma V, Ravindran P, Kumar PP. Plant hormone-mediated regulation of stress responses. BMC Plant Biol. 2016;16(1):86. 27079791 10.1186/s12870-016-0771-y PMC4831116

[21] Shigenaga AM, Argueso CT. No hormone to rule them all: Interactions of plant hormones during the responses of plants to pathogens. Sem Cell Dev Biol. 2016;56:174–89. 10.1016/j.semcdb.2016.06.005 27312082

[22] Nguyen D, Rieu I, Mariani C, Dam NM. How plants handle multiple stresses: hormonal interactions underlying responses to abiotic stress and insect herbivory. Plant Mol Biol. 2016;91(6):727–40. doi: 10.1007/s11103-016-0481-8 27095445 10.1007/s11103-016-0481-8 PMC4932144

[23] Tuteja N. Abscisic acid and abiotic stress signaling. Plant Signal Behav. 2007;2(3):135–8. 19516981 10.4161/psb.2.3.4156 PMC2634038

[24] Xu J, Audenaert K, Hofte M, De Vleesschauwer D. Abscisic acid promotes susceptibility to the rice leaf blight pathogen *Xanthomonas oryzae* pv *oryzae* by suppressing salicylic acid-mediated defenses. PLoS ONE. 2013;8(6):e67413 doi: 10.1371/journal.pone.0067413 23826294 10.1371/journal.pone.0067413 PMC3694875

[25] Xu J, Zhou L, Venturi V, He Y-W, Kojima M, Sakakibari H et al Phytohormone-mediated interkingdom signaling shapes the outcome of rice-*Xanthomonas oryzae* pv. *oryzae* interactions. BMC Plant Biol. 2015;15:10 doi: 10.1186/s12870-014-0411-3 25605284 10.1186/s12870-014-0411-3 PMC4307914

[26] Le Thanh T, Thumanu K, Wongkaew S, Boonkerd N, Teaumroong N, Phansak P et al Salicylic acid-induced accumulation of biochemical components associated with resistance against *Xanthomonas oryzae* pv. *oryzae* in rice. J Plant Interact. 2017;12(1):108–20.

[27] Jiang C-J, Shimono M, Sugano S, Kojima M, Yazawa K, Yoshida R et al Abscisic acid interacts antagonistically with salicylic acid signaling pathway in rice-*Magnaporthe grisea* interaction. Mol Plant-Microbe Interact. 2010;23(6):791–8. doi: 10.1094/MPMI-23-6-0791 20459318 10.1094/MPMI-23-6-0791

[28] Xiong L, Yang Y. Disease resistance and abiotic stress tolerance in rice are inversely modulated by an abscisic acid–inducible mitogen-activated protein kinase. Plant Cell. 2003;15(3):745–59. doi: 10.1105/tpc.008714 12615946 10.1105/tpc.008714 PMC150027

[29] Triplett LR, Hamilton J, Buell C, Tisserat NA, Verdier V, Zink F et al Genomic analysis of *Xanthomonas oryzae* isolates from rice grown in the United States reveals substantial divergence from known *X*. *oryzae* pathovars. Appl Environ Microbiol. 2011;77(12):3930–7. doi: 10.1128/AEM.00028-11 21515727 10.1128/AEM.00028-11 PMC3131649

[30] Garg R, Tyagi AK, Jain M. Microarray analysis reveals overlapping and specific transcriptional responses to different plant hormones in rice. Plant Signal Behav. 2012;7(8):951–6. doi: 10.4161/psb.20910 22827941 10.4161/psb.20910 PMC3474693

[31] Gómez-Porras JL, Riaño-Pachón DM, Dreyer I, Mayer JE, Mueller-Roeber B. Genome-wide analysis of ABA-responsive elements ABRE and CE3 reveals divergent patterns in Arabidopsis and rice. BMC Genomics. 2007;8:260 doi: 10.1186/1471-2164-8-260 17672917 10.1186/1471-2164-8-260 PMC2000901

[32] Higo K, Ugawa Y, Iwamoto M, Korenaga T. Plant cis-acting regulatory DNA elements (PLACE) database: 1999. Nucleic Acids Res. 1999;27(1):297–300. 9847208 10.1093/nar/27.1.297 PMC148163

[33] Barnabás B, Jäger K, Fehér A. The effect of drought and heat stress on reproductive processes in cereals. Plant Cell Environ. 2008;31(1):11–38. doi: 10.1111/j.1365-3040.2007.01727.x 17971069 10.1111/j.1365-3040.2007.01727.x

[34] Zhang Y, Mian M, Chekhovskiy K, So S, Kupfer D, Lai H et al Differential gene expression in Festuca under heat stress conditions. J Exp Bot. 2005;56(413):897–907. doi: 10.1093/jxb/eri082 15710639 10.1093/jxb/eri082

[35] Berger S, Sinha AK, Roitsch T. Plant physiology meets phytopathology: plant primary metabolism and plant–pathogen interactions. J Exp Bot. 2007;58(15–16):4019–26. doi: 10.1093/jxb/erm298 18182420 10.1093/jxb/erm298

[36] Suzuki N, Bassil E, Hamilton JS, Inupakutika MA, Zandalinas SI, Tripathy D et al ABA Is required for plant acclimation to a combination of salt and heat stress. PLoS ONE. 2016;11(1):e0147625 doi: 10.1371/journal.pone.0147625 26824246 10.1371/journal.pone.0147625 PMC4733103

[37] Zandalinas SI, Balfagón D, Arbona V, Gómez-Cadenas A, Inupakutika MA, Mittler R. ABA is required for the accumulation of APX1 and MBF1c during a combination of water deficit and heat stress. J Exp Bot. 2016;67(18):5381–90. doi: 10.1093/jxb/erw299 27497287 10.1093/jxb/erw299 PMC5049388

[38] Hu X, Wang W, Li C, Zhang J, Lin F, Zhang A et al Cross-talks between Ca^2+^/CaM and H_2_O_2_ in abscisic acid-induced antioxidant defense in leaves of maize plants exposed to water stress. Plant Growth Regul. 2008;55:183–98.

[39] Baron KN, Schroeder DF, Stasolla C. Transcriptional response of abscisic acid (ABA) metabolism and transport to cold and heat stress applied at the reproductive stage of development in *Arabidopsis thaliana*. Plant Sci. 2012;188–189:48–59. doi: 10.1016/j.plantsci.2012.03.001 22525244 10.1016/j.plantsci.2012.03.001

[40] Lim CW, Baek W, Jung J, Kim J-H, Lee SC. Function of ABA in stomatal defense against biotic and drought stresses. Int J Mol Sci. 2015;16(7):15251–70. doi: 10.3390/ijms160715251 26154766 10.3390/ijms160715251 PMC4519898

[41] Mang H-G, Qian W, Zhu Y, Qian J, Kang H-G, Klessig DF et al Abscisic acid deficiency antagonizes high-temperature inhibition of disease resistance through enhancing nuclear accumulation of resistance proteins SNC1 and RPS4 in *Arabidopsis*. Plant Cell. 2012;24(3):1271–84. doi: 10.1105/tpc.112.096198 22454454 10.1105/tpc.112.096198 PMC3336126

[42] Narsai R, Wang C, Chen J, Wu J, Shou H, Whelan J. Antagonistic, overlapping and distinct responses to biotic stress in rice (*Oryza sativa*) and interactions with abiotic stress. BMC Genomics. 2013;14:93 doi: 10.1186/1471-2164-14-93 23398910 10.1186/1471-2164-14-93 PMC3616870

[43] Rizhsky L, Liang H, Shuman J, Shulaev V, Davletova S, Mittler R. When defense pathways collide. The response of Arabidopsis to a combination of drought and heat stress. Plant Physiol. 2004;134(4):1683–96. doi: 10.1104/pp.103.033431 15047901 10.1104/pp.103.033431 PMC419842

[44] Mittler R. Abiotic stress, the field environment and stress combination. Trends Plant Sci. 2006;11(1):15–9. doi: 10.1016/j.tplants.2005.11.002 16359910 10.1016/j.tplants.2005.11.002

[45] Verdier V, Triplett LR, Hummel AW, Corral R, Cernadas RA, Schmidt CL et al Transcription activator‐like (TAL) effectors targeting *OsSWEET* genes enhance virulence on diverse rice (*Oryza sativa*) varieties when expressed individually in a TAL effector‐deficient strain of *Xanthomonas oryzae*. New Phytol. 2012;196(4):1197–207. doi: 10.1111/j.1469-8137.2012.04367.x 23078195 10.1111/j.1469-8137.2012.04367.x

[46] Triplett LR, Cohen SP, Heffelfinger C, Schmidt CL, Huerta AI, Tekete C et al A resistance locus in the American heirloom rice variety Carolina Gold Select is triggered by TAL effectors with diverse predicted targets and is effective against African strains of *Xanthomonas oryzae* pv. *oryzicola*. Plant J. 2016;87(5):472–83. doi: 10.1111/tpj.13212 27197779 10.1111/tpj.13212 PMC5030141

[47] Karganilla A, Natural M, Ou S. A comparative study of culture media for *Xanthomonas oryzae*. Philippine Agr. 1973;57:141–52.

[48] Reimers PJ, Leach JE. Race-specific resistance to *Xanthomonas oryzae* pv. *oryzae* conferred by bacterial blight resistance gene *Xa-10* in rice (*Oryza sativa*) involves accumulation of a lignin-like substance in host tissues. Physiol Mol Plant Pathol. 1991;38(1):39–55.

[49] R Core Team. R: A Language and Environment for Statistical Computing. In: R Foundation for Statistical Computing Vienna, Austria 2016 Available from: https://www.R-project.org/.

[50] Lu G, Gao C, Zheng X, Han B. Identification of OsbZIP72 as a positive regulator of ABA response and drought tolerance in rice. Planta. 2009;229(3):605–15. doi: 10.1007/s00425-008-0857-3 19048288 10.1007/s00425-008-0857-3

[51] Lee SH, Sakuraba Y, Lee T, Kim KW, An G, Lee HY et al Mutation of Oryza sativa CORONATINE INSENSITIVE 1b (OsCOI1b) delays leaf senescence. J Integr Plant Biol. 2015;57(6):562–76. doi: 10.1111/jipb.12276 25146897 10.1111/jipb.12276

[52] Livak KJ, Schmittgen TD. Analysis of relative gene expression data using real-time quantitative PCR and the 2− ΔΔCT method. Methods. 2001;25(4):402–8. doi: 10.1006/meth.2001.1262 11846609 10.1006/meth.2001.1262

[53] Gordon A, Hannon G. FASTX-Toolkit: FASTQ/A short-reads preprocessing tools. 2010. Available from: http://hannonlab.cshl.edu/fastx_toolkit.

[54] Kawahara Y, de la Bastide M, Hamilton JP, Kanamori H, McCombie WR, Ouyang S et al Improvement of the *Oryza sativa* Nipponbare reference genome using next generation sequence and optical map data. Rice. 2013;6:4 doi: 10.1186/1939-8433-6-4 24280374 10.1186/1939-8433-6-4 PMC5395016

[55] Kim D, Pertea G, Trapnell C, Pimentel H, Kelley R, Salzberg SL. TopHat2: accurate alignment of transcriptomes in the presence of insertions, deletions and gene fusions. Genome Biol. 2013;14:R36 doi: 10.1186/gb-2013-14-4-r36 23618408 10.1186/gb-2013-14-4-r36 PMC4053844

[56] Anders S, Pyl PT, Huber W. HTSeq–a Python framework to work with high-throughput sequencing data. Bioinformatics. 2015;31(2):166–9. doi: 10.1093/bioinformatics/btu638 25260700 10.1093/bioinformatics/btu638 PMC4287950

[57] Robinson MD, McCarthy DJ, Smyth GK. edgeR: a Bioconductor package for differential expression analysis of digital gene expression data. Bioinformatics. 2010;26(1):139–40. doi: 10.1093/bioinformatics/btp616 19910308 10.1093/bioinformatics/btp616 PMC2796818

[58] McCarthy DJ, Chen Y, Smyth GK. Differential expression analysis of multifactor RNA-Seq experiments with respect to biological variation. Nucleic Acids Res. 2012;40(10):4288–97. doi: 10.1093/nar/gks042 22287627 10.1093/nar/gks042 PMC3378882

[59] Gregory R. Warnes BB, Lodewijk Bonebakker, Robert Gentleman, Wolfgang Huber Andy Liaw, Thomas Lumley, Martin Maechler, Arni Magnusson, Steffen Moeller, Marc Schwartz, Bill Venables. gplots: Various R programming tools for plotting data. The Comprehensive R Archive Network. 2016.

[60] Bailey TL. DREME: motif discovery in transcription factor ChIP-seq data. Bioinformatics. 2011;27(12):1653–9. doi: 10.1093/bioinformatics/btr261 21543442 10.1093/bioinformatics/btr261 PMC3106199

[61] Mahony S, Benos PV. STAMP: a web tool for exploring DNA-binding motif similarities. Nucleic Acids Res. 2007;35:W253–W8. doi: 10.1093/nar/gkm272 17478497 10.1093/nar/gkm272 PMC1933206

