# Supplementary figures and images for: RNA-Seq analysis reveals insight into enhanced rice Xa7-mediated bacterial blight resistance at high temperature
Source: PLoS One. 2017 Nov 6;12(11):e0187625. doi: 10.1371/journal.pone.0187625 (PMC5673197; doi:10.1371/journal.pone.0187625)

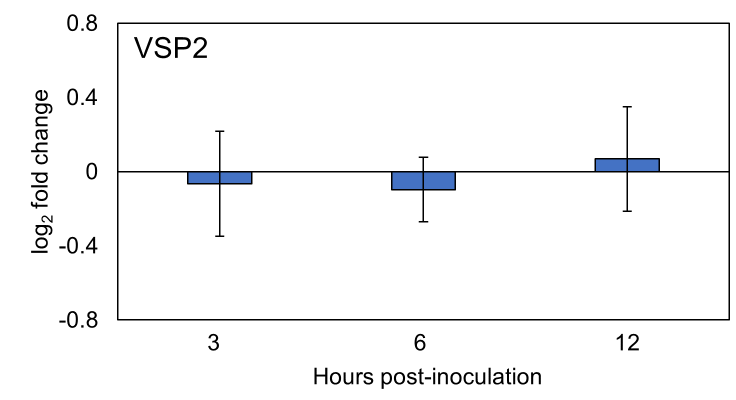

Supplement: S1 Fig — There were no significant differences in expression of the wound-responsive jasmonic acid marker VSP2 at 3, 6, and 12 h post-mock inoculation at high temperature relative to low temperature (Student’s t-test, p > 0.05). Error bars represent SEM (n = 6). (PNG) [file pone.0187625.s001.png]

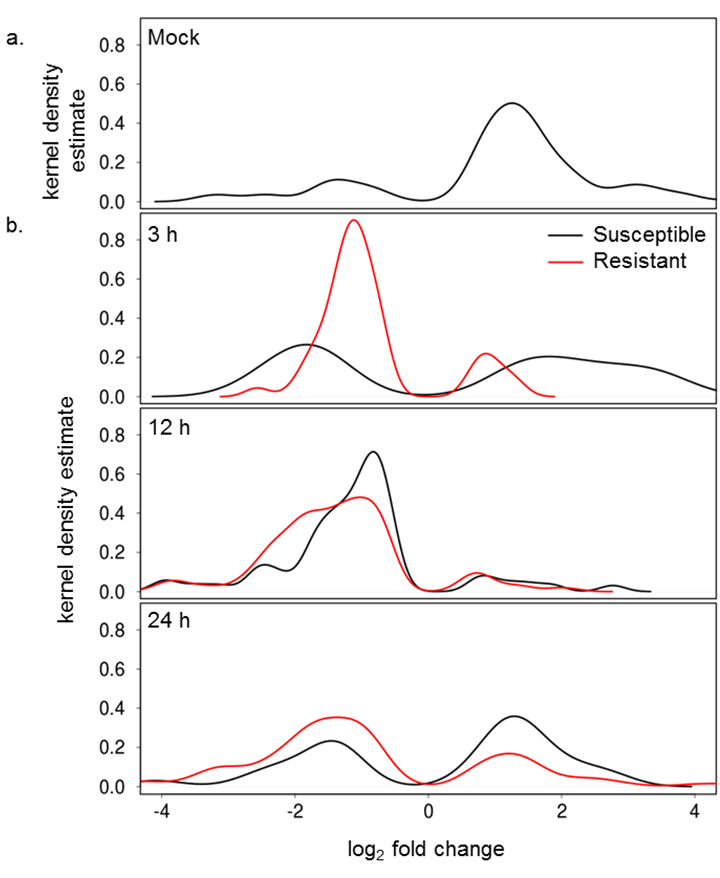

Supplement: S2 Fig — (a) Kernel density estimate of log2 fold change for SA up-regulated genes differentially regulated by high temperature in mock inoculated plants. (b) Kernel density estimates of log2 fold change for SA up-regulated genes differentially regulated by high temperature in plants during susceptible and resistant interactions at 3, 12, and 24 hpi. (PNG) [file pone.0187625.s002.png]
