# Supplementary material for: RNA-Seq analysis reveals insight into enhanced rice Xa7-mediated bacterial blight resistance at high temperature
Source: PLoS One. 2017 Nov 6;12(11):e0187625. doi: 10.1371/journal.pone.0187625 (PMC5673197; doi:10.1371/journal.pone.0187625)
Supplement: S1 Table — (DOCX) [file pone.0187625.s003.docx]

**Table S1: Sequencing reads and mapping summary statistics.**

| **Samples** | **Repeat** | **Raw Reads** | | | **Mapped Reads** | | **Mapped to Genes** | |
| --- | --- | --- | --- | --- | --- | --- | --- | --- |
|  |  | **Total** | **High-quality** | **%** | **Total** | **%** | **Total** | **%** |
| Mock  NT 6 hpi | 1 | 28856641 | 28797259 | 99.79 | 27803387 | 96.35 | 25304614 | 87.69 |
|  | 2 | 30695442 | 30638812 | 99.82 | 29600586 | 96.43 | 26848926 | 87.47 |
| Mock  HT 6 hpi | 1 | 41141433 | 41070267 | 99.83 | 39789983 | 96.72 | 36328352 | 88.30 |
|  | 2 | 30123157 | 30054334 | 99.77 | 28809619 | 95.64 | 26283199 | 87.25 |
| Susceptible  NT 3 hpi | 1 | 18821068 | 18655171 | 99.12 | 18152259 | 96.45 | 16166375 | 85.90 |
|  | 2 | 13444900 | 13408422 | 99.73 | 13058055 | 97.12 | 11616326 | 86.40 |
| Susceptible  NT 12 hpi | 1 | 22988953 | 22926231 | 99.73 | 22301041 | 97.01 | 20033428 | 87.14 |
|  | 2 | 18575425 | 18526120 | 99.73 | 18001933 | 96.91 | 16199997 | 87.21 |
| Susceptible  NT 24 hpi | 1 | 23032633 | 22976160 | 99.75 | 22291085 | 96.78 | 19999269 | 86.83 |
|  | 2 | 12956182 | 12924337 | 99.75 | 12517108 | 96.61 | 11153937 | 86.09 |
| Susceptible  HT 3 hpi | 1 | 23971411 | 23908160 | 99.74 | 23267192 | 97.06 | 20852465 | 86.99 |
|  | 2 | 21613616 | 21555636 | 99.73 | 20975915 | 97.05 | 18787524 | 86.92 |
| Susceptible  HT 12 hpi | 1 | 27982082 | 27894994 | 99.69 | 27068780 | 96.74 | 24365034 | 87.07 |
|  | 2 | 23750702 | 23678271 | 99.70 | 22952651 | 96.64 | 20657166 | 86.97 |
| Susceptible  HT 24 hpi | 1 | 12757097 | 12721172 | 99.72 | 12310495 | 96.50 | 10965566 | 85.96 |
|  | 2 | 18194844 | 18140633 | 99.70 | 17532615 | 96.36 | 15647360 | 86.00 |
| Resistant  NT 3 hpi | 1 | 23216413 | 23154090 | 99.73 | 22531147 | 97.05 | 20277909 | 87.34 |
|  | 2 | 17686011 | 17640774 | 99.74 | 17163755 | 97.05 | 15428030 | 87.23 |
| Resistant  NT 12 hpi | 1 | 13651450 | 13615929 | 99.74 | 13229846 | 96.91 | 11903526 | 87.20 |
|  | 2 | 22218398 | 22157390 | 99.73 | 21529837 | 96.90 | 19382683 | 87.24 |
| Resistant  NT 24 hpi | 1 | 22393658 | 22329613 | 99.71 | 21673815 | 96.79 | 19035385 | 85.00 |
|  | 2 | 14395418 | 14360572 | 99.76 | 13919943 | 96.70 | 12372688 | 85.95 |
| Resistant  HT 3 hpi | 1 | 22577109 | 22517295 | 99.74 | 21889456 | 96.95 | 19612136 | 86.87 |
|  | 2 | 25936686 | 25856236 | 99.69 | 25140318 | 96.93 | 22637373 | 87.28 |
| Resistant  HT 12 hpi | 1 | 17682014 | 17631071 | 99.71 | 17117963 | 96.81 | 15248118 | 86.24 |
|  | 2 | 24896378 | 24828591 | 99.73 | 24115576 | 96.86 | 21627216 | 86.87 |
| Resistant  HT 24 hpi | 1 | 26980561 | 26721295 | 99.04 | 25723891 | 95.34 | 22705900 | 84.16 |
|  | 2 | 20501057 | 20440854 | 99.71 | 19770695 | 96.44 | 17521343 | 85.47 |

NT = normal temperature; HT = high temperature; hpi = hours post-inoculation; percentages are per total raw reads per row
