# Supplementary material for: RNA-Seq analysis reveals insight into enhanced rice Xa7-mediated bacterial blight resistance at high temperature
Source: PLoS One. 2017 Nov 6;12(11):e0187625. doi: 10.1371/journal.pone.0187625 (PMC5673197; doi:10.1371/journal.pone.0187625)
Supplement: S2 Table — (DOCX) [file pone.0187625.s004.docx]

**Table S2: GO term enrichment analysis for genes differentially expressed by high temperature.**

**Table A. Terms enriched in genes up-regulated by high temperature alone.**

| **GO Term** | **# Regulated** | **# Not Regulated** | **log odds ratio** | **FDR-corrected p-value** |
| --- | --- | --- | --- | --- |
| biosynthetic process | 205 / 917 | 3491 / 26832 | 0.945671 | 9.80E-13 |
| response to endogenous stimulus | 87 | 1024 | 1.402012 | 3.13E-12 |
| cellular process | 291 | 5655 | 0.80055 | 4.56E-12 |
| metabolic process | 285 | 5503 | 0.806354 | 4.68E-12 |
| response to stress | 162 | 2576 | 1.015332 | 4.88E-12 |
| carbohydrate metabolic process | 65 | 724 | 1.460453 | 3.61E-10 |
| response to abiotic stimulus | 104 | 1588 | 1.024647 | 1.62E-08 |
| response to biotic stimulus | 63 | 785 | 1.291966 | 5.36E-08 |
| secondary metabolic process | 29 | 300 | 1.530591 | 1.94E-05 |
| translation | 2 | 550 | -3.24492 | 2.89E-05 |
| multicellular organismal development | 66 | 1094 | 0.868266 | 0.000197 |
| signal transduction | 66 | 1113 | 0.84242 | 0.00031 |
| transport | 94 | 1876 | 0.604408 | 0.002109 |
| lipid metabolic process | 44 | 736 | 0.838311 | 0.004324 |
| nucleobase, nucleoside, nucleotide and nucleic acid metabolic process | 125 | 2772 | 0.455095 | 0.010172 |
| cell growth | 22 | 309 | 1.077804 | 0.012999 |
| cell differentiation | 23 | 378 | 0.849111 | 0.040416 |

The columns indicate GO term, number of annotated genes upregulated by high temperature, number of annotated genes not upregulated, log_2_ odds ratio, and FDR-corrected p-value. The first row includes the total number of genes upregulated and not upregulated.

**Table B. Terms enriched in genes down-regulated by high temperature alone.**

| **GO Term** | **# Regulated** | **# Not Regulated** | **log odds ratio** | **FDR-corrected p-value** |
| --- | --- | --- | --- | --- |
| photosynthesis | 33 / 594 | 140 / 27155 | 3.50486 | 5.14E-20 |
| generation of precursor metabolites and energy | 28 | 233 | 2.51504 | 6.54E-11 |
| metabolic process | 165 | 5623 | 0.559515 | 0.000396 |
| response to stress | 86 | 2652 | 0.646269 | 0.001977 |
| response to biotic stimulus | 33 | 815 | 0.927582 | 0.005596 |
| protein modification process | 33 | 2326 | -0.66904 | 0.030064 |

**Table C. Terms enriched in genes up-regulated by high temperature during the susceptible interaction at 3h post-inoculation.**

| **GO Term** | **# Regulated** | **# Not Regulated** | **log odds ratio** | **FDR-corrected p-value** |
| --- | --- | --- | --- | --- |
| response to endogenous stimulus | 18 / 153 | 1093 / 27596 | 1.693224 | 0.000341 |
| response to stress | 32 | 2706 | 1.283078 | 0.000396 |
| cellular homeostasis | 7 | 184 | 2.836594 | 0.000706 |
| response to abiotic stimulus | 20 | 1672 | 1.221842 | 0.005964 |
| biosynthetic process | 35 | 3661 | 0.956224 | 0.006095 |
| lipid metabolic process | 12 | 768 | 1.572274 | 0.006948 |
| cellular process | 50 | 5896 | 0.838013 | 0.007315 |
| metabolic process | 47 | 5741 | 0.756078 | 0.017197 |
| transport | 21 | 1949 | 1.066544 | 0.017207 |
| catabolic process | 14 | 1205 | 1.14204 | 0.034787 |

**Table D. Terms enriched in genes up-regulated by high temperature during the susceptible interaction at 3h post-inoculation.**

| **GO Term** | **# Regulated** | **# Not Regulated** | **log odds ratio** | **FDR-corrected p-value** |
| --- | --- | --- | --- | --- |
| photosynthesis | 9 / 135 | 164 / 27614 | 3.579208 | 2.37E-06 |
| metabolic process | 50 | 5738 | 1.165798 | 0.000143 |
| generation of precursor metabolites and energy | 7 | 254 | 2.558439 | 0.002049 |
| response to stress | 26 | 2712 | 1.131691 | 0.004598 |
| response to biotic stimulus | 12 | 836 | 1.644191 | 0.005297 |
| transport | 18 | 1952 | 1.016838 | 0.039546 |

**Table E: Terms enriched in genes up-regulated by high temperature during the susceptible interaction at 12h post-inoculation.**

| **GO Term** | **# Regulated** | **# Not Regulated** | **log odds ratio** | **FDR-corrected p-value** |
| --- | --- | --- | --- | --- |
| photosynthesis | 40 / 1192 | 133 / 26557 | 2.786277 | 7.84E-17 |
| metabolic process | 374 | 5414 | 0.83711 | 1.80E-16 |
| generation of precursor metabolites and energy | 37 | 224 | 1.913428 | 3.45E-09 |
| translation | 57 | 495 | 1.403305 | 2.67E-08 |
| cellular process | 329 | 5617 | 0.508111 | 2.86E-06 |
| response to abiotic stimulus | 116 | 1576 | 0.773817 | 7.17E-06 |
| secondary metabolic process | 32 | 297 | 1.286863 | 0.000203 |
| protein modification process | 65 | 2294 | -0.7107 | 0.000396 |
| cellular component organization | 79 | 1120 | 0.689756 | 0.001344 |
| DNA metabolic process | 4 | 379 | -2.09807 | 0.003226 |
| lipid metabolic process | 54 | 726 | 0.756408 | 0.003857 |
| anatomical structure morphogenesis | 14 | 633 | -1.03589 | 0.01928 |
| signal transduction | 34 | 1145 | -0.61569 | 0.046676 |

**Table F: Terms enriched in genes down-regulated by high temperature during the susceptible interaction at 12h post-inoculation.**

| **GO Term** | **# Regulated** | **# Not Regulated** | **log odds ratio** | **FDR-corrected p-value** |
| --- | --- | --- | --- | --- |
| metabolic process | 288 / 820 | 5500 / 26929 | 1.077367 | 6.45E-20 |
| response to stress | 156 | 2582 | 1.148121 | 2.61E-14 |
| cellular process | 271 | 5675 | 0.887225 | 2.11E-13 |
| response to biotic stimulus | 70 | 778 | 1.649835 | 3.49E-13 |
| response to abiotic stimulus | 93 | 1599 | 1.019617 | 1.12E-07 |
| biosynthetic process | 167 | 3529 | 0.762756 | 2.05E-07 |
| catabolic process | 69 | 1150 | 1.042995 | 2.93E-06 |
| transport | 97 | 1873 | 0.84455 | 8.41E-06 |
| response to endogenous stimulus | 60 | 1051 | 0.959631 | 7.09E-05 |
| signal transduction | 62 | 1117 | 0.919233 | 0.000122 |
| photosynthesis | 15 | 158 | 1.65898 | 0.00144 |
| translation | 4 | 548 | -2.0771 | 0.003257 |
| generation of precursor metabolites and energy | 18 | 243 | 1.301911 | 0.006334 |
| secondary metabolic process | 21 | 308 | 1.184424 | 0.007211 |
| ripening | 2 | 2 | 4 | 0.021854 |
| protein modification process | 92 | 2267 | 0.460225 | 0.026131 |
| nucleobase, nucleoside, nucleotide and nucleic acid metabolic process | 109 | 2788 | 0.409728 | 0.035725 |
| lipid metabolic process | 36 | 744 | 0.69336 | 0.037443 |
| carbohydrate metabolic process | 36 | 753 | 0.675527 | 0.039403 |

**Table G: Terms enriched in genes up-regulated by high temperature during the susceptible interaction at 24h post-inoculation.**

| **GO Term** | **# Regulated** | **# Not Regulated** | **log odds ratio** | **FDR-corrected p-value** |
| --- | --- | --- | --- | --- |
| metabolic process | 119 / 355 | 5669 / 27394 | 0.950988 | 3.45E-07 |
| secondary metabolic process | 14 | 315 | 1.819591 | 0.00072 |
| cell communication | 9 | 160 | 2.146664 | 0.002287 |
| response to extracellular stimulus | 9 | 172 | 2.041607 | 0.003613 |
| response to stress | 55 | 2683 | 0.756591 | 0.004922 |
| cellular process | 102 | 5844 | 0.573038 | 0.005964 |
| response to abiotic stimulus | 36 | 1656 | 0.81142 | 0.015777 |
| translation | 1 | 551 | -2.85057 | 0.044306 |
| cellular homeostasis | 7 | 184 | 1.57306 | 0.044482 |

**Table H: Terms enriched in genes down-regulated by high temperature during the susceptible interaction at 24h post-inoculation.**

| **GO Term** | **# Regulated** | **# Not Regulated** | **log odds ratio** | **FDR-corrected p-value** |
| --- | --- | --- | --- | --- |
| translation | 94 / 440 | 458 / 27309 | 3.993133 | 1.29E-66 |
| cellular process | 132 | 5814 | 0.664888 | 0.000197 |
| nucleobase, nucleoside, nucleotide and nucleic acid metabolic process | 69 | 2828 | 0.687915 | 0.004183 |
| response to abiotic stimulus | 41 | 1651 | 0.676164 | 0.027297 |

**Table I: Terms enriched in genes up-regulated by high temperature during the resistant interaction at 3h post-inoculation.**

| **GO Term** | **# Regulated** | **# Not Regulated** | **log odds ratio** | **FDR-corrected p-value** |
| --- | --- | --- | --- | --- |
| response to stress | 112 / 694 | 2626 / 27055 | 0.840902 | 2.49E-06 |
| nucleobase, nucleoside, nucleotide and nucleic acid metabolic process | 112 | 2785 | 0.746727 | 2.89E-05 |
| metabolic process | 195 | 5593 | 0.585462 | 4.01E-05 |
| biosynthetic process | 133 | 3563 | 0.645321 | 0.000114 |
| DNA metabolic process | 21 | 362 | 1.202742 | 0.004627 |
| cellular homeostasis | 13 | 178 | 1.527673 | 0.005964 |
| cell communication | 12 | 157 | 1.592287 | 0.006194 |
| carbohydrate metabolic process | 34 | 755 | 0.844304 | 0.011597 |
| response to abiotic stimulus | 62 | 1630 | 0.614618 | 0.01347 |
| cellular process | 181 | 5765 | 0.382906 | 0.014815 |
| response to extracellular stimulus | 11 | 170 | 1.349301 | 0.025769 |
| protein modification process | 40 | 2319 | -0.61395 | 0.02932 |
| biological_process | 195 | 6437 | 0.325023 | 0.039403 |
| cellular component organization | 44 | 1155 | 0.603026 | 0.049575 |

**Table J: Terms enriched in genes down-regulated by high temperature during the resistant interaction at 3h post-inoculation.**

| **GO Term** | **# Regulated** | **# Not Regulated** | **log odds ratio** | **FDR-corrected p-value** |
| --- | --- | --- | --- | --- |
| biosynthetic process | 186 / 769 | 3510 / 26980 | 1.093712 | 8.14E-15 |
| cellular process | 260 | 5686 | 0.93644 | 3.32E-14 |
| metabolic process | 246 | 5542 | 0.86427 | 6.81E-12 |
| signal transduction | 74 | 1105 | 1.31851 | 1.34E-09 |
| response to biotic stimulus | 56 | 792 | 1.377337 | 4.81E-08 |
| response to stress | 123 | 2615 | 0.827826 | 1.02E-06 |
| response to endogenous stimulus | 61 | 1050 | 1.089909 | 5.80E-06 |
| protein modification process | 106 | 2253 | 0.812007 | 8.05E-06 |
| transport | 92 | 1878 | 0.8618 | 1.10E-05 |
| lipid metabolic process | 46 | 734 | 1.186359 | 2.06E-05 |
| translation | 2 | 550 | -2.98527 | 0.000305 |
| nucleobase, nucleoside, nucleotide and nucleic acid metabolic process | 116 | 2781 | 0.629213 | 0.000354 |
| response to abiotic stimulus | 74 | 1618 | 0.739794 | 0.000866 |
| carbohydrate metabolic process | 40 | 749 | 0.943031 | 0.001753 |
| DNA metabolic process | 1 | 382 | -3.44727 | 0.002853 |
| ripening | 2 | 2 | 4 | 0.019673 |

**Table K: Terms enriched in genes up-regulated by high temperature during the resistant interaction at 12h post-inoculation.**

| **GO Term** | **# Regulated** | **# Not Regulated** | **log odds ratio** | **FDR-corrected p-value** |
| --- | --- | --- | --- | --- |
| photosynthesis | 117 / 3107 | 56 / 24642 | 4 | 5.52E-67 |
| generation of precursor metabolites and energy | 106 | 155 | 2.4804 | 1.55E-32 |
| metabolic process | 873 | 4915 | 0.650226 | 2.01E-22 |
| cellular process | 820 | 5126 | 0.45003 | 6.01E-11 |
| lipid metabolic process | 126 | 654 | 0.633475 | 0.000203 |
| response to abiotic stimulus | 233 | 1459 | 0.366461 | 0.004325 |
| cellular homeostasis | 37 | 154 | 0.939214 | 0.004616 |
| biosynthetic process | 474 | 3222 | 0.260376 | 0.005389 |
| anatomical structure morphogenesis | 47 | 600 | -0.69791 | 0.005596 |
| multicellular organismal development | 96 | 1064 | -0.49914 | 0.005596 |
| carbohydrate metabolic process | 117 | 672 | 0.482069 | 0.008022 |
| cell cycle | 18 | 277 | -0.96138 | 0.017389 |
| DNA metabolic process | 26 | 357 | -0.79821 | 0.019005 |
| secondary metabolic process | 54 | 275 | 0.649111 | 0.020539 |
| protein modification process | 224 | 2135 | -0.28616 | 0.024521 |
| cell growth | 22 | 309 | -0.82987 | 0.026929 |
| regulation of gene expression, epigenetic | 4 | 110 | -1.79338 | 0.027297 |
| translation | 82 | 470 | 0.480375 | 0.031394 |

**Table L: Terms enriched in genes down-regulated by high temperature during the resistant interaction at 12h post-inoculation.**

| **GO Term** | **# Regulated** | **# Not Regulated** | **log odds ratio** | **FDR-corrected p-value** |
| --- | --- | --- | --- | --- |
| translation | 192 / 2878 | 360 / 24871 | 2.283178 | 1.23E-52 |
| cellular process | 809 | 5137 | 0.5879 | 2.22E-17 |
| response to stress | 415 | 2323 | 0.710554 | 8.83E-15 |
| response to abiotic stimulus | 280 | 1412 | 0.841171 | 3.32E-14 |
| transport | 305 | 1665 | 0.725172 | 7.58E-12 |
| response to endogenous stimulus | 190 | 921 | 0.87891 | 6.18E-11 |
| response to biotic stimulus | 148 | 700 | 0.905197 | 3.86E-09 |
| metabolic process | 731 | 5057 | 0.416852 | 1.04E-08 |
| nucleobase, nucleoside, nucleotide and nucleic acid metabolic process | 395 | 2502 | 0.509178 | 6.43E-08 |
| biosynthetic process | 479 | 3217 | 0.427565 | 9.31E-07 |
| multicellular organismal development | 156 | 1004 | 0.447174 | 0.004183 |
| secondary metabolic process | 53 | 276 | 0.742262 | 0.006948 |
| signal transduction | 155 | 1024 | 0.407732 | 0.010172 |
| catabolic process | 158 | 1061 | 0.38355 | 0.015688 |
| biological_process | 745 | 5887 | 0.172863 | 0.035975 |
| post-embryonic development | 119 | 798 | 0.380862 | 0.0426 |

**Table M: Terms enriched in genes up-regulated by high temperature during the resistant interaction at 24h post-inoculation.**

| **GO Term** | **# Regulated** | **# Not Regulated** | **log odds ratio** | **FDR-corrected p-value** |
| --- | --- | --- | --- | --- |
| metabolic process | 343 / 1022 | 5445 / 26727 | 0.982175 | 5.14E-20 |
| protein modification process | 162 | 2197 | 1.073233 | 1.98E-13 |
| cellular process | 311 | 5635 | 0.712123 | 1.33E-10 |
| response to stress | 163 | 2575 | 0.832484 | 1.04E-08 |
| secondary metabolic process | 32 | 297 | 1.524688 | 7.51E-06 |
| signal transduction | 77 | 1102 | 0.922715 | 1.21E-05 |
| response to endogenous stimulus | 72 | 1039 | 0.906732 | 4.01E-05 |
| response to biotic stimulus | 57 | 791 | 0.954347 | 0.000143 |
| response to abiotic stimulus | 96 | 1596 | 0.707874 | 0.000206 |
| lipid metabolic process | 49 | 731 | 0.841423 | 0.002126 |
| cell death | 22 | 275 | 1.082071 | 0.012924 |
| pollen-pistil interaction | 9 | 75 | 1.658983 | 0.017207 |
| cell communication | 14 | 155 | 1.252102 | 0.024684 |
| response to extracellular stimulus | 14 | 167 | 1.143942 | 0.034011 |

**Table N: Terms enriched in genes down-regulated by high temperature during the resistant interaction at 24h post-inoculation.**

| **GO Term** | **# Regulated** | **# Not Regulated** | **log odds ratio** | **FDR-corrected p-value** |
| --- | --- | --- | --- | --- |
| translation | 220 / 1598 | 332 / 26151 | 3.633829 | 8.05E-125 |
| protein modification process | 82 | 2277 | -0.8157 | 2.09E-06 |
| response to abiotic stimulus | 149 | 1543 | 0.714487 | 2.49E-06 |
| response to stress | 207 | 2531 | 0.474825 | 0.000313 |
| cellular process | 396 | 5550 | 0.291467 | 0.005497 |
| regulation of gene expression, epigenetic | 0 | 114 | -4 | 0.009868 |
| nucleobase, nucleoside, nucleotide and nucleic acid metabolic process | 201 | 2696 | 0.32507 | 0.02071 |
| carbohydrate metabolic process | 62 | 727 | 0.498315 | 0.048263 |
| response to biotic stimulus | 66 | 782 | 0.483949 | 0.04917 |
