# Supplementary material for: RNA-Seq analysis reveals insight into enhanced rice Xa7-mediated bacterial blight resistance at high temperature
Source: PLoS One. 2017 Nov 6;12(11):e0187625. doi: 10.1371/journal.pone.0187625 (PMC5673197; doi:10.1371/journal.pone.0187625)
Supplement: S3 Table — (DOCX) [file pone.0187625.s005.docx]

**Table S3: Differential expression of rice wound response genes from NCBI GEO Accession** **GSE77097.**

| **Wound response gene** | **Mock**  **6 hpi** | **Susceptible**  **3 hpi** | **Susceptible**  **12 hpi** | **Susceptible**  **24 hpi** | **Resistant**  **3 hpi** | **Resistant**  **12 hpi** | **Resistant**  **24 hpi** |
| --- | --- | --- | --- | --- | --- | --- | --- |
| LOC_Os01g03740 | *n.s.* | *n.s.* | *n.s.* | *n.s.* | *n.s.* | Down | Down |
| LOC_Os01g08860 | *n.s.* | *n.s.* | Up | *n.s.* | *n.s.* | *n.s.* | Up |
| LOC_Os01g13950 | *n.s.* | *n.s.* | *n.s.* | *n.s.* | *n.s.* | Down | *n.s.* |
| LOC_Os01g16810 | *n.s.* | *n.s.* | *n.s.* | *n.s.* | *n.s.* | *n.s.* | *n.s.* |
| LOC_Os01g20980 | *n.s.* | *n.s.* | *n.s.* | *n.s.* | *n.s.* | *n.s.* | *n.s.* |
| LOC_Os01g32380 | *n.s.* | *n.s.* | *n.s.* | *n.s.* | *n.s.* | *n.s.* | *n.s.* |
| LOC_Os01g38580 | *n.s.* | Up | *n.s.* | *n.s.* | *n.s.* | *n.s.* | Up |
| LOC_Os01g50440 | *n.s.* | *n.s.* | *n.s.* | Down | *n.s.* | Down | Down |
| LOC_Os01g57240 | *n.s.* | *n.s.* | *n.s.* | *n.s.* | *n.s.* | *n.s.* | Down |
| LOC_Os01g61610 | *n.s.* | *n.s.* | Up | *n.s.* | *n.s.* | *n.s.* | Down |
| LOC_Os01g62190 | *n.s.* | *n.s.* | *n.s.* | *n.s.* | *n.s.* | Down | *n.s.* |
| LOC_Os01g62980 | *n.s.* | Up | *n.s.* | *n.s.* | *n.s.* | *n.s.* | *n.s.* |
| LOC_Os01g65950 | *n.s.* | *n.s.* | *n.s.* | *n.s.* | *n.s.* | *n.s.* | *n.s.* |
| LOC_Os02g02930 | *n.s.* | *n.s.* | *n.s.* | *n.s.* | *n.s.* | *n.s.* | *n.s.* |
| LOC_Os02g26810 | *n.s.* | *n.s.* | *n.s.* | *n.s.* | *n.s.* | *n.s.* | Up |
| LOC_Os02g32060 | *n.s.* | *n.s.* | *n.s.* | *n.s.* | *n.s.* | *n.s.* | *n.s.* |
| LOC_Os02g32520 | Up | *n.s.* | Down | *n.s.* | Down | Down | Down |
| LOC_Os02g46830 | *n.s.* | *n.s.* | *n.s.* | *n.s.* | *n.s.* | *n.s.* | *n.s.* |
| LOC_Os02g47370 | *n.s.* | *n.s.* | *n.s.* | *n.s.* | *n.s.* | Down | *n.s.* |
| LOC_Os02g47810 | Down | *n.s.* | *n.s.* | *n.s.* | Down | Down | Down |
| LOC_Os02g48770 | *n.s.* | Up | *n.s.* | *n.s.* | *n.s.* | *n.s.* | *n.s.* |
| LOC_Os02g50810 | *n.s.* | *n.s.* | *n.s.* | *n.s.* | *n.s.* | Down | Down |
| LOC_Os02g51040 | *n.s.* | *n.s.* | *n.s.* | *n.s.* | *n.s.* | *n.s.* | *n.s.* |
| LOC_Os02g52150 | *n.s.* | *n.s.* | *n.s.* | Up | Down | *n.s.* | Up |
| LOC_Os02g52380 | *n.s.* | *n.s.* | *n.s.* | *n.s.* | Down | Down | Down |
| LOC_Os03g04870 | *n.s.* | *n.s.* | *n.s.* | *n.s.* | *n.s.* | Down | *n.s.* |
| LOC_Os03g04890 | *n.s.* | *n.s.* | *n.s.* | *n.s.* | Down | Down | *n.s.* |
| LOC_Os03g12370 | *n.s.* | *n.s.* | *n.s.* | *n.s.* | Down | Down | *n.s.* |
| LOC_Os03g16350 | *n.s.* | *n.s.* | *n.s.* | *n.s.* | Up | Down | Down |
| LOC_Os03g17810 | *n.s.* | *n.s.* | *n.s.* | *n.s.* | *n.s.* | *n.s.* | Down |
| LOC_Os03g24390 | *n.s.* | *n.s.* | *n.s.* | *n.s.* | *n.s.* | *n.s.* | *n.s.* |
| LOC_Os03g45450 | *n.s.* | *n.s.* | *n.s.* | *n.s.* | *n.s.* | *n.s.* | *n.s.* |
| LOC_Os03g50960 | *n.s.* | *n.s.* | *n.s.* | *n.s.* | *n.s.* | Down | *n.s.* |
| LOC_Os03g53900 | *n.s.* | *n.s.* | Down | *n.s.* | *n.s.* | Down | Down |
| LOC_Os03g57200 | *n.s.* | *n.s.* | Down | Up | *n.s.* | Down | Up |
| LOC_Os03g58500 | *n.s.* | *n.s.* | *n.s.* | *n.s.* | *n.s.* | Down | Down |
| LOC_Os03g60560 | Up | *n.s.* | *n.s.* | *n.s.* | *n.s.* | Down | Down |
| LOC_Os03g61360 | *n.s.* | *n.s.* | Down | *n.s.* | *n.s.* | Down | *n.s.* |
| LOC_Os03g63390 | *n.s.* | *n.s.* | *n.s.* | *n.s.* | *n.s.* | *n.s.* | Down |
| LOC_Os04g14690 | *n.s.* | *n.s.* | Down | Down | Down | Down | Down |
| LOC_Os04g27060 | *n.s.* | *n.s.* | Down | *n.s.* | *n.s.* | *n.s.* | *n.s.* |
| LOC_Os04g33920 | *n.s.* | *n.s.* | *n.s.* | *n.s.* | *n.s.* | *n.s.* | *n.s.* |
| LOC_Os04g43200 | *n.s.* | *n.s.* | *n.s.* | *n.s.* | *n.s.* | Down | Down |
| LOC_Os04g46400 | Up | *n.s.* | *n.s.* | *n.s.* | *n.s.* | *n.s.* | Down |
| LOC_Os04g47620 | *n.s.* | *n.s.* | *n.s.* | *n.s.* | *n.s.* | *n.s.* | *n.s.* |
| LOC_Os04g52780 | *n.s.* | Down | *n.s.* | *n.s.* | Down | *n.s.* | *n.s.* |
| LOC_Os04g53606 | *n.s.* | *n.s.* | *n.s.* | Down | *n.s.* | Down | Down |
| LOC_Os04g56430 | *n.s.* | *n.s.* | *n.s.* | *n.s.* | *n.s.* | Down | Down |
| LOC_Os04g56690 | *n.s.* | *n.s.* | *n.s.* | *n.s.* | *n.s.* | *n.s.* | *n.s.* |
| LOC_Os04g57430 | *n.s.* | *n.s.* | *n.s.* | *n.s.* | *n.s.* | *n.s.* | *n.s.* |
| LOC_Os04g57860 | *n.s.* | *n.s.* | *n.s.* | *n.s.* | *n.s.* | Down | *n.s.* |
| LOC_Os05g10740 | *n.s.* | *n.s.* | *n.s.* | *n.s.* | *n.s.* | *n.s.* | *n.s.* |
| LOC_Os05g30760 | *n.s.* | *n.s.* | *n.s.* | *n.s.* | *n.s.* | Down | *n.s.* |
| LOC_Os05g39310 | *n.s.* | *n.s.* | *n.s.* | *n.s.* | *n.s.* | Down | Down |
| LOC_Os05g39320 | *n.s.* | *n.s.* | *n.s.* | *n.s.* | *n.s.* | Down | Down |
| LOC_Os05g45100 | *n.s.* | *n.s.* | *n.s.* | *n.s.* | *n.s.* | Down | *n.s.* |
| LOC_Os05g46510 | *n.s.* | *n.s.* | *n.s.* | *n.s.* | *n.s.* | *n.s.* | Down |
| LOC_Os05g49940 | *n.s.* | *n.s.* | *n.s.* | *n.s.* | *n.s.* | Down | Down |
| LOC_Os06g04080 | *n.s.* | *n.s.* | *n.s.* | Down | *n.s.* | Down | Down |
| LOC_Os06g05000 | *n.s.* | *n.s.* | *n.s.* | *n.s.* | *n.s.* | *n.s.* | *n.s.* |
| LOC_Os06g05420 | *n.s.* | *n.s.* | *n.s.* | *n.s.* | Down | *n.s.* | Down |
| LOC_Os06g10130 | *n.s.* | *n.s.* | Down | *n.s.* | *n.s.* | Down | *n.s.* |
| LOC_Os06g11210 | *n.s.* | *n.s.* | *n.s.* | *n.s.* | *n.s.* | *n.s.* | *n.s.* |
| LOC_Os06g11280 | *n.s.* | *n.s.* | Down | *n.s.* | *n.s.* | Down | *n.s.* |
| LOC_Os06g11290 | *n.s.* | *n.s.* | Down | *n.s.* | Down | Down | *n.s.* |
| LOC_Os06g35520 | *n.s.* | Down | Down | Down | Down | *n.s.* | Down |
| LOC_Os06g39240 | *n.s.* | *n.s.* | *n.s.* | *n.s.* | *n.s.* | *n.s.* | Up |
| LOC_Os06g39390 | *n.s.* | *n.s.* | *n.s.* | *n.s.* | *n.s.* | *n.s.* | *n.s.* |
| LOC_Os06g47270 | *n.s.* | *n.s.* | *n.s.* | *n.s.* | *n.s.* | *n.s.* | *n.s.* |
| LOC_Os06g49760 | *n.s.* | *n.s.* | *n.s.* | *n.s.* | *n.s.* | *n.s.* | *n.s.* |
| LOC_Os07g23570 | *n.s.* | *n.s.* | *n.s.* | *n.s.* | *n.s.* | *n.s.* | Down |
| LOC_Os07g44140 | *n.s.* | *n.s.* | Down | *n.s.* | *n.s.* | Down | *n.s.* |
| LOC_Os08g02030 | *n.s.* | *n.s.* | *n.s.* | *n.s.* | *n.s.* | Down | Down |
| LOC_Os08g04350 | *n.s.* | *n.s.* | *n.s.* | *n.s.* | Down | Down | *n.s.* |
| LOC_Os08g04800 | *n.s.* | *n.s.* | *n.s.* | *n.s.* | *n.s.* | *n.s.* | *n.s.* |
| LOC_Os08g23290 | *n.s.* | *n.s.* | Down | *n.s.* | *n.s.* | *n.s.* | *n.s.* |
| LOC_Os08g24300 | *n.s.* | *n.s.* | Down | *n.s.* | *n.s.* | *n.s.* | *n.s.* |
| LOC_Os08g39730 | *n.s.* | *n.s.* | *n.s.* | *n.s.* | *n.s.* | Down | Down |
| LOC_Os08g40910 | *n.s.* | *n.s.* | *n.s.* | *n.s.* | *n.s.* | *n.s.* | *n.s.* |
| LOC_Os08g41290 | *n.s.* | *n.s.* | *n.s.* | *n.s.* | Down | Down | *n.s.* |
| LOC_Os08g41670 | *n.s.* | *n.s.* | *n.s.* | *n.s.* | *n.s.* | *n.s.* | *n.s.* |
| LOC_Os08g43334 | *n.s.* | *n.s.* | *n.s.* | *n.s.* | *n.s.* | Down | *n.s.* |
| LOC_Os09g20220 | *n.s.* | *n.s.* | Down | *n.s.* | *n.s.* | Down | *n.s.* |
| LOC_Os09g27260 | *n.s.* | *n.s.* | *n.s.* | *n.s.* | *n.s.* | Down | *n.s.* |
| LOC_Os09g30250 | *n.s.* | *n.s.* | *n.s.* | *n.s.* | *n.s.* | *n.s.* | *n.s.* |
| LOC_Os09g34250 | *n.s.* | *n.s.* | Down | Up | *n.s.* | Down | Up |
| LOC_Os09g36619 | *n.s.* | *n.s.* | *n.s.* | *n.s.* | *n.s.* | Up | *n.s.* |
| LOC_Os10g23310 | *n.s.* | *n.s.* | *n.s.* | *n.s.* | *n.s.* | Down | *n.s.* |
| LOC_Os10g23820 | *n.s.* | *n.s.* | *n.s.* | *n.s.* | *n.s.* | Down | *n.s.* |
| LOC_Os10g25400 | *n.s.* | *n.s.* | *n.s.* | *n.s.* | *n.s.* | Down | Down |
| LOC_Os10g35300 | *n.s.* | *n.s.* | *n.s.* | *n.s.* | *n.s.* | Down | Down |
| LOC_Os10g38340 | *n.s.* | *n.s.* | *n.s.* | *n.s.* | Down | Down | *n.s.* |
| LOC_Os10g39920 | *n.s.* | *n.s.* | *n.s.* | *n.s.* | *n.s.* | Down | *n.s.* |
| LOC_Os10g41020 | *n.s.* | *n.s.* | *n.s.* | *n.s.* | *n.s.* | Down | *n.s.* |
| LOC_Os11g03910 | *n.s.* | *n.s.* | *n.s.* | *n.s.* | *n.s.* | *n.s.* | Down |
| LOC_Os11g31060 | *n.s.* | *n.s.* | *n.s.* | *n.s.* | *n.s.* | *n.s.* | *n.s.* |
| LOC_Os11g35330 | *n.s.* | *n.s.* | *n.s.* | *n.s.* | *n.s.* | Down | *n.s.* |
| LOC_Os12g13890 | *n.s.* | *n.s.* | Down | Down | Down | *n.s.* | Down |
| LOC_Os12g18560 | *n.s.* | *n.s.* | *n.s.* | *n.s.* | *n.s.* | *n.s.* | *n.s.* |
| LOC_Os12g26290 | *n.s.* | *n.s.* | *n.s.* | Up | *n.s.* | *n.s.* | *n.s.* |
| **Up-regulated** | 3 | 3 | 2 | 4 | 1 | 1 | 7 |
| **Down-regulated** | 1 | 2 | 16 | 6 | 15 | 50 | 33 |
| **Not differentially expressed** | 96 | 95 | 82 | 90 | 84 | 49 | 60 |

Up = up-regulated at high temperature; Down = down-regulated at high temperature; *n.s.* = not significantly differentially expressed at high temperature
