# Supplementary material for: RNA-Seq analysis reveals insight into enhanced rice Xa7-mediated bacterial blight resistance at high temperature
Source: PLoS One. 2017 Nov 6;12(11):e0187625. doi: 10.1371/journal.pone.0187625 (PMC5673197; doi:10.1371/journal.pone.0187625)
Supplement: S4 Table — (DOCX) [file pone.0187625.s006.docx]

**Table S4: Hormone biosynthesis genes that were differentially expressed due to high temperature in at least one treatment/time point.**

| **Locus** | **Pathway** | **Function** | **Mock logFC** | **Mock FDR** | **S3 logFC** | **S3 FDR** | **S12 logFC** | **S12 FDR** | **S24 logFC** | **S24 FDR** | **R3 logFC** | **R3 FDR** | **R12 logFC** | **R12 FDR** | **R24 logFC** | **R24 FDR** |
| --- | --- | --- | --- | --- | --- | --- | --- | --- | --- | --- | --- | --- | --- | --- | --- | --- |
| LOC_Os03g44380 | abscisic acid biosynthesis | 9-cis-epoxycarotenoid dioxygenase 1, chloroplast precursor, putative, expressed | 1.1044 | 0.0013 | 0.8203 | 0.1020 | 0.0297 | 0.9792 | -0.2894 | 0.8328 | -1.7493 | 0.0014 | -0.8194 | 0.1269 | -1.2887 | 0.0191 |
| LOC_Os07g05940 | abscisic acid biosynthesis | 9-cis-epoxycarotenoid dioxygenase 1, chloroplast precursor, putative, expressed | 7.1078 | 0.0000 | 3.2167 | 0.0005 | -0.0943 | 0.9514 | -1.0021 | 0.6042 | -1.7932 | 0.1628 | -0.8614 | 0.5642 | -6.6624 | 0.0000 |
| LOC_Os12g44310 | abscisic acid biosynthesis | carotenoid cleavage dioxygenase, putative, expressed | -0.1737 | 0.7847 | -0.5817 | 0.4257 | 0.6723 | 0.0086 | 0.6032 | 0.0382 | -0.0998 | 0.8134 | 1.3608 | 0.0000 | 0.7300 | 0.0096 |
| LOC_Os03g57690 | auxin biosynthesis | aldehyde oxidase, putative, expressed | -0.0670 | 0.8998 | -0.5567 | 0.3188 | -0.3711 | 0.0719 | 0.1834 | 0.7828 | 0.4021 | 0.1557 | -0.0656 | 0.8373 | 0.7959 | 0.0010 |
| LOC_Os07g18120 | auxin biosynthesis | aldehyde oxidase, putative, expressed | -0.4256 | 0.5591 | 0.4621 | 0.6666 | -0.1204 | 0.9045 | -1.1450 | 0.2033 | 0.2646 | 0.5193 | -1.2341 | 0.0000 | -2.2105 | 0.0000 |
| LOC_Os10g04860 | auxin biosynthesis | aldehyde oxidase, putative, expressed | -0.4641 | 0.4196 | -0.5188 | 0.6352 | -0.0851 | 0.9207 | 0.4602 | 0.2554 | 0.7289 | 0.0792 | 0.9343 | 0.0001 | 0.6706 | 0.0350 |
| LOC_Os04g02754 | auxin biosynthesis | amidase family protein, putative, expressed | -0.1455 | 0.8525 | 0.2935 | 0.8104 | 0.5526 | 0.0091 | 0.3023 | 0.4667 | 1.0063 | 0.0018 | 0.5784 | 0.0022 | 0.2951 | 0.5655 |
| LOC_Os04g02780 | auxin biosynthesis | amidase family protein, putative, expressed | -0.4214 | 0.1857 | 0.0715 | 0.9791 | 0.6060 | 0.0026 | -0.2249 | 0.6768 | 0.4596 | 0.2280 | 1.0534 | 0.0000 | -0.1802 | 0.5832 |
| LOC_Os04g10460 | auxin biosynthesis | amidase, putative, expressed | -0.0541 | 0.9444 | 1.4414 | 0.1037 | 0.6625 | 0.0387 | -0.3190 | 0.6742 | 2.1565 | 0.0000 | 0.7082 | 0.0497 | -1.2447 | 0.0014 |
| LOC_Os04g55050 | auxin biosynthesis | amidase, putative, expressed | -0.1942 | 0.6639 | 0.3078 | 0.8270 | 0.6968 | 0.0000 | -0.0476 | 0.9564 | 0.7991 | 0.0004 | 0.9768 | 0.0000 | 0.1299 | 0.7522 |
| LOC_Os11g33090 | auxin biosynthesis | amidase, putative, expressed | -0.0496 | 0.9444 | -0.0860 | 0.9701 | -0.4773 | 0.1339 | -0.1888 | 0.7874 | -0.3306 | 0.5410 | -0.6859 | 0.0025 | 0.0622 | 0.9093 |
| LOC_Os06g23140 | auxin biosynthesis | copper methylamine oxidase precursor, putative, expressed | -0.4882 | 0.5706 | -1.9193 | 0.0063 | -0.8464 | 0.2763 | 0.3647 | 0.7839 | -0.6456 | 0.4549 | -0.5229 | 0.2141 | -1.0728 | 0.2495 |
| LOC_Os01g12770 | auxin biosynthesis | cytochrome P450, putative, expressed | -0.8601 | 0.0030 | -1.5848 | 0.2393 | 0.4937 | 0.0991 | 0.1669 | 0.8038 | 0.0934 | 0.9400 | 1.6893 | 0.0000 | 0.1876 | 0.7899 |
| LOC_Os01g56380 | auxin biosynthesis | decarboxylase, putative, expressed | 0.8318 | 0.0255 | 0.9890 | 0.1847 | 0.7685 | 0.0049 | 0.6498 | 0.1829 | 0.3512 | 0.5781 | 1.4218 | 0.0000 | 1.0488 | 0.0046 |
| LOC_Os07g25590 | auxin biosynthesis | decarboxylase, putative, expressed | -0.3633 | 0.2875 | 0.1223 | 0.9699 | 0.1203 | 0.7239 | 0.0939 | 0.9196 | 0.3196 | 0.5057 | 0.7972 | 0.0002 | 0.3466 | 0.3525 |
| LOC_Os01g12490 | auxin biosynthesis | flavin monooxygenase, putative, expressed | 0.8074 | 0.1425 | 1.2499 | 0.0431 | 0.4872 | 0.4778 | 1.3865 | 0.0774 | 1.3395 | 0.0012 | 1.0053 | 0.0507 | 1.0392 | 0.0158 |
| LOC_Os03g06654 | auxin biosynthesis | flavin monooxygenase, putative, expressed | 0.1147 | 0.8450 | 0.8172 | 0.4195 | 0.6979 | 0.0411 | -0.4968 | 0.4951 | 1.7167 | 0.0014 | 0.9812 | 0.0005 | -0.7372 | 0.1000 |
| LOC_Os04g03980 | auxin biosynthesis | flavin monooxygenase, putative, expressed | 0.8555 | 0.0932 | -0.6053 | 0.6576 | -0.4303 | 0.6619 | 0.8780 | 0.6473 | -1.9454 | 0.0000 | -0.2880 | 0.5433 | 1.0388 | 0.2317 |
| LOC_Os07g25540 | auxin biosynthesis | flavin monooxygenase, putative, expressed | 0.8090 | 0.3187 | 2.7662 | 0.0030 | 1.6405 | 0.0634 | -0.3885 | 0.9096 | 1.4377 | 0.0142 | 1.6699 | 0.0228 | 1.5972 | 0.0343 |
| LOC_Os12g32750 | auxin biosynthesis | flavin monooxygenase, putative, expressed | 1.3242 | 0.0029 | 2.6178 | 0.0897 | 0.8407 | 0.5418 | 1.3783 | 0.0760 | 1.8103 | 0.0466 | 3.0088 | 0.0001 | 2.5487 | 0.0000 |
| LOC_Os01g02500 | auxin biosynthesis | glutamyl-tRNA amidotransferase, putative, expressed | -0.3361 | 0.5874 | -0.0535 | 0.9899 | 0.8487 | 0.1007 | 0.5805 | 0.5520 | 0.0491 | 0.9610 | 0.4046 | 0.4171 | 1.4904 | 0.0002 |
| LOC_Os02g42330 | auxin biosynthesis | nitrilase, putative, expressed | 0.2930 | 0.4435 | -0.5849 | 0.8207 | 0.1987 | 0.5629 | 0.5235 | 0.1685 | 1.0034 | 0.0004 | 0.9528 | 0.0000 | 0.4974 | 0.1027 |
| LOC_Os05g39310 | auxin biosynthesis | thiamine pyrophosphate enzyme, C-terminal TPP binding domain containing protein, expressed | 0.5359 | 0.7908 | 0.2211 | 0.9671 | -0.2969 | 0.7477 | -0.6348 | 0.7352 | -1.1000 | 0.1167 | -1.6953 | 0.0008 | -2.1467 | 0.0051 |
| LOC_Os05g39320 | auxin biosynthesis | thiamine pyrophosphate enzyme, C-terminal TPP binding domain containing protein, expressed | -0.6992 | 0.7343 | -0.5063 | 0.8003 | -0.5355 | 0.4046 | -1.3833 | 0.0807 | -0.7562 | 0.1804 | -1.5903 | 0.0000 | -1.6296 | 0.0009 |
| LOC_Os03g59570 | cytokinin biosynthesis | IPP transferase, putative, expressed | -1.4972 | 0.2811 | -1.1636 | 0.7935 | -1.8652 | 0.0026 | -4.6541 | 0.0000 | 0.1661 | 0.9340 | -1.7053 | 0.0225 | -0.4743 | 0.7653 |
| LOC_Os06g51350 | cytokinin biosynthesis | IPP transferase, putative, expressed | -0.3570 | 0.5010 | -0.3418 | 0.8541 | 0.4685 | 0.2651 | 0.0573 | 0.9624 | 0.4185 | 0.5678 | 1.0034 | 0.0035 | 0.1285 | 0.8484 |
| LOC_Os01g39860 | ethylene biosynthesis | 1-aminocyclopropane-1-carboxylate oxidase protein, putative, expressed | 1.3432 | 0.0000 | 0.3735 | 0.7113 | -1.0618 | 0.0236 | 0.3490 | 0.8557 | 1.1487 | 0.0000 | -1.1038 | 0.0435 | 0.7318 | 0.0391 |
| LOC_Os05g05680 | ethylene biosynthesis | 1-aminocyclopropane-1-carboxylate oxidase, putative, expressed | 0.3559 | 0.3195 | -0.1392 | 0.9525 | 0.2032 | 0.6073 | 0.3025 | 0.5991 | 1.0068 | 0.0000 | -0.3072 | 0.2553 | 0.2899 | 0.4549 |
| LOC_Os01g09700 | ethylene biosynthesis | aminotransferase, classes I and II, domain containing protein, expressed | 1.8504 | 0.0000 | 1.4421 | 0.0844 | 0.7062 | 0.2400 | -0.4342 | 0.8054 | -0.4479 | 0.3744 | -0.8170 | 0.0754 | 0.5199 | 0.6432 |
| LOC_Os04g48850 | ethylene biosynthesis | aminotransferase, classes I and II, domain containing protein, expressed | 1.4437 | 0.1293 | -1.1707 | 0.2174 | -0.3583 | 0.8571 | 1.3792 | 0.1501 | 0.7910 | 0.1347 | -2.1544 | 0.0035 | 2.2917 | 0.0000 |
| LOC_Os06g03990 | ethylene biosynthesis | aminotransferase, classes I and II, domain containing protein, expressed | 0.6529 | 0.0048 | 0.1561 | 0.9318 | 0.0119 | 0.9728 | 0.3944 | 0.3334 | 0.1076 | 0.7886 | -0.2419 | 0.3096 | 0.6416 | 0.0145 |
| LOC_Os01g22010 | ethylene biosynthesis | S-adenosylmethionine synthetase, putative, expressed | 1.2231 | 0.0002 | -0.0779 | 0.9726 | -0.3249 | 0.2690 | 0.0353 | 0.9677 | -0.3042 | 0.2926 | -0.6831 | 0.0036 | 0.0210 | 0.9709 |
| LOC_Os05g04510 | ethylene biosynthesis | S-adenosylmethionine synthetase, putative, expressed | 0.7395 | 0.0349 | 0.1688 | 0.9166 | -0.3055 | 0.1922 | 0.2764 | 0.5134 | -0.3967 | 0.1675 | -0.7828 | 0.0001 | 0.2530 | 0.6295 |
| LOC_Os06g11200 | jasmonic acid biosynthesis | 12-oxophytodienoate reductase, putative, expressed | 0.7421 | 0.5234 | -2.0795 | 0.0554 | -0.1356 | 0.9318 | 0.6263 | 0.6802 | -0.3072 | 0.8409 | -2.5032 | 0.0028 | 0.0676 | 0.9540 |
| LOC_Os06g11240 | jasmonic acid biosynthesis | 12-oxophytodienoate reductase, putative, expressed | 0.7279 | 0.0644 | -0.2560 | 0.9389 | -0.6223 | 0.0040 | 0.8377 | 0.0964 | -0.2811 | 0.6690 | -0.9479 | 0.0001 | 0.7812 | 0.0541 |
| LOC_Os06g11280 | jasmonic acid biosynthesis | 12-oxophytodienoate reductase, putative, expressed | 0.8508 | 0.3810 | -0.6658 | 0.8299 | -1.7666 | 0.0000 | 0.1773 | 0.9471 | -1.1061 | 0.0373 | -2.4428 | 0.0000 | -0.3139 | 0.6432 |
| LOC_Os06g11290 | jasmonic acid biosynthesis | 12-oxophytodienoate reductase, putative, expressed | 0.6286 | 0.3484 | -0.8989 | 0.5818 | -1.7975 | 0.0000 | 0.2477 | 0.8021 | -1.2844 | 0.0022 | -2.5672 | 0.0000 | -0.7095 | 0.2081 |
| LOC_Os08g35740 | jasmonic acid biosynthesis | 12-oxophytodienoate reductase, putative, expressed | 0.6828 | 0.0026 | 0.2769 | 0.7711 | -0.0744 | 0.8185 | 0.0979 | 0.8699 | -0.4637 | 0.1717 | -0.2391 | 0.3808 | -0.1638 | 0.6017 |
| LOC_Os03g32314 | jasmonic acid biosynthesis | allene oxide cyclase 4, chloroplast precursor, putative, expressed | 0.5084 | 0.0453 | -0.3824 | 0.6145 | 0.4903 | 0.0081 | 0.3973 | 0.2737 | -0.5691 | 0.1233 | 0.2581 | 0.3920 | 0.3143 | 0.2769 |
| LOC_Os12g26290 | jasmonic acid biosynthesis | alpha-DOX2, putative, expressed | 0.9558 | 0.0221 | 0.0045 | 1.0000 | 0.0451 | 0.9403 | 0.9769 | 0.0002 | -0.4284 | 0.0943 | 0.3787 | 0.0491 | 0.9163 | 0.2150 |
| LOC_Os03g55800 | jasmonic acid biosynthesis | cytochrome P450, putative, expressed | 0.7787 | 0.0512 | -0.2279 | 0.9408 | 0.9067 | 0.0010 | 0.9008 | 0.3145 | -1.0599 | 0.0575 | -0.1676 | 0.6484 | 0.3122 | 0.4797 |
| LOC_Os01g40070 | jasmonic acid biosynthesis | expressed protein | 1.9343 | 0.0000 | -0.2228 | 0.9187 | -0.0022 | 0.9990 | 0.4922 | 0.5994 | -0.9205 | 0.0111 | -0.9005 | 0.0001 | -0.2630 | 0.4115 |
| LOC_Os03g17260 | jasmonic acid biosynthesis | expressed protein | -0.0569 | 0.9811 | -1.1679 | 0.2264 | -0.2080 | 0.7702 | -2.1027 | 0.1082 | -0.0687 | 0.9597 | 1.0184 | 0.0574 | -3.5773 | 0.0019 |
| LOC_Os05g51520 | jasmonic acid biosynthesis | expressed protein | -0.2044 | 0.6869 | -0.1691 | 0.9218 | -0.5318 | 0.1756 | -0.0857 | 0.9278 | -0.1562 | 0.7708 | -0.6595 | 0.0003 | -0.3674 | 0.2592 |
| LOC_Os09g04880 | jasmonic acid biosynthesis | expressed protein | -0.6304 | 0.0285 | 0.1070 | 0.9699 | 0.2524 | 0.3094 | 0.0336 | 0.9632 | 0.6230 | 0.0454 | 0.8846 | 0.0001 | 0.3662 | 0.2273 |
| LOC_Os12g37260 | jasmonic acid biosynthesis | lipoxygenase 2.1, chloroplast precursor, putative, expressed | -0.5505 | 0.1200 | -0.3357 | 0.8724 | -0.0837 | 0.8022 | -0.4473 | 0.3297 | 1.5456 | 0.4568 | 0.8241 | 0.0004 | 0.3143 | 0.4550 |
| LOC_Os03g08220 | jasmonic acid biosynthesis | lipoxygenase protein, putative, expressed | 0.4651 | 0.1309 | 0.2197 | 0.8299 | 0.4311 | 0.0490 | 0.2138 | 0.6741 | -0.1233 | 0.7414 | 0.2117 | 0.3387 | 0.6385 | 0.0054 |
| LOC_Os04g37430 | jasmonic acid biosynthesis | lipoxygenase protein, putative, expressed | 0.8276 | 0.0008 | -0.7284 | 0.4452 | -0.1305 | 0.7105 | 0.4092 | 0.6453 | -0.6460 | 0.0375 | -1.0842 | 0.0000 | 0.4966 | 0.1102 |
| LOC_Os08g39840 | jasmonic acid biosynthesis | lipoxygenase, chloroplast precursor, putative, expressed | 2.0424 | 0.0012 | -1.3406 | 0.0294 | -1.5515 | 0.0000 | -0.1069 | 0.9173 | 0.1620 | 0.7799 | -2.7601 | 0.0000 | -0.3921 | 0.4818 |
| LOC_Os08g39850 | jasmonic acid biosynthesis | lipoxygenase, chloroplast precursor, putative, expressed | 0.6299 | 0.5446 | -1.1011 | 0.4484 | -1.9496 | 0.0012 | -1.1928 | 0.2845 | -0.5206 | 0.4804 | -3.1737 | 0.0000 | -0.2199 | 0.8018 |
| LOC_Os02g10120 | jasmonic acid biosynthesis | lipoxygenase, putative, expressed | -0.6724 | 0.2807 | 1.3931 | 0.4452 | 4.1583 | 0.0000 | 1.7283 | 0.0000 | -0.1942 | 0.8683 | 2.8080 | 0.0000 | 0.5833 | 0.3516 |
| LOC_Os03g49380 | jasmonic acid biosynthesis | lipoxygenase, putative, expressed | 2.3353 | 0.0000 | -0.4308 | 0.7528 | 0.0309 | 0.9578 | 0.6714 | 0.2488 | -1.2532 | 0.0030 | -0.9731 | 0.0002 | -0.2605 | 0.6940 |
| LOC_Os05g23880 | jasmonic acid biosynthesis | lipoxygenase, putative, expressed | 0.3327 | 0.3034 | -0.0171 | 1.0000 | 1.0601 | 0.0000 | 0.8975 | 0.0323 | 0.5661 | 0.3326 | 1.6633 | 0.0000 | 1.2690 | 0.0003 |
| LOC_Os04g57440 | jasmonic acid biosynthesis | oryzain beta chain precursor, putative, expressed | -0.2458 | 0.5874 | 0.0037 | 1.0000 | -0.5353 | 0.0236 | -0.4829 | 0.3988 | -0.2586 | 0.4510 | -0.8195 | 0.0000 | -1.1564 | 0.0000 |
| LOC_Os01g47330 | jasmonic acid biosynthesis | ribosomal protein L7/L12 C-terminal domain containing protein, expressed | -0.6993 | 0.0686 | 0.0838 | 0.9879 | 1.3666 | 0.0000 | 0.0411 | 0.9635 | 0.9943 | 0.1118 | 1.8922 | 0.0000 | -0.2106 | 0.6533 |
| LOC_Os02g46970 | salicylic acid biosynthesis | AMP-binding domain containing protein, expressed | 1.1789 | 0.0339 | -0.4661 | 0.9417 | 2.0935 | 0.0000 | -0.2297 | 0.8844 | 0.4041 | 0.3863 | 2.0982 | 0.0000 | -1.4200 | 0.0341 |
| LOC_Os04g58710 | salicylic acid biosynthesis | AMP-binding domain containing protein, expressed | -2.3568 | 0.1153 | 0.1833 | 0.9546 | -0.4740 | 0.2286 | 0.5050 | 0.3510 | 1.0942 | 0.0001 | -0.4571 | 0.1965 | -0.4489 | 0.3470 |
| LOC_Os06g44620 | salicylic acid biosynthesis | AMP-binding domain containing protein, expressed | 0.8740 | 0.0002 | 0.1609 | 0.8992 | -0.0612 | 0.8834 | -0.1225 | 0.8359 | -0.2436 | 0.4691 | -0.6597 | 0.0255 | -0.1163 | 0.7277 |
| LOC_Os08g14760 | salicylic acid biosynthesis | AMP-binding domain containing protein, expressed | 0.7709 | 0.0194 | -0.5452 | 0.8694 | 1.1253 | 0.0021 | 0.5701 | 0.4213 | 0.3135 | 0.5192 | 1.8467 | 0.0000 | 1.0209 | 0.0780 |
| LOC_Os08g34790 | salicylic acid biosynthesis | AMP-binding domain containing protein, expressed | 0.8295 | 0.2799 | -0.1000 | 0.9743 | -0.4124 | 0.2130 | 0.1803 | 0.9220 | 0.2310 | 0.6902 | -1.1059 | 0.0000 | 0.6416 | 0.2469 |
| LOC_Os10g42800 | salicylic acid biosynthesis | AMP-binding enzyme, putative, expressed | -1.3573 | 0.0001 | -0.8105 | 0.2911 | -1.2235 | 0.0031 | 0.4374 | 0.3606 | -0.7329 | 0.0111 | -0.5166 | 0.0238 | 0.3607 | 0.2966 |
| LOC_Os09g19734 | salicylic acid biosynthesis | isochorismate synthase 1, chloroplast precursor, putative, expressed | -0.2747 | 0.3902 | 0.2285 | 0.8299 | 0.1871 | 0.4713 | -0.4175 | 0.1929 | 0.2941 | 0.2986 | 0.0371 | 0.8954 | -0.6325 | 0.0098 |
| LOC_Os02g41630 | salicylic acid biosynthesis | phenylalanine ammonia-lyase, putative, expressed | 1.2301 | 0.0009 | 0.1102 | 0.9743 | 0.1873 | 0.4864 | 0.0162 | 0.9838 | 0.1070 | 0.8357 | -0.4938 | 0.0963 | -0.0953 | 0.8159 |
| LOC_Os02g41650 | salicylic acid biosynthesis | phenylalanine ammonia-lyase, putative, expressed | 1.2701 | 0.0000 | -0.0860 | 0.9743 | -0.3344 | 0.1224 | -0.0476 | 0.9610 | -0.6563 | 0.0056 | -0.9583 | 0.0001 | -0.1347 | 0.7446 |
| LOC_Os02g41670 | salicylic acid biosynthesis | phenylalanine ammonia-lyase, putative, expressed | -0.5925 | 0.6957 | -0.3074 | 0.8970 | -0.4401 | 0.5708 | 0.8550 | 0.5275 | 0.1126 | 0.9073 | -0.8810 | 0.0063 | 1.5874 | 0.0001 |
| LOC_Os02g41680 | salicylic acid biosynthesis | phenylalanine ammonia-lyase, putative, expressed | -0.8253 | 0.1796 | -0.9637 | 0.3968 | -0.1754 | 0.7048 | 0.1149 | 0.9522 | -0.3232 | 0.4890 | 0.3035 | 0.1414 | 0.7836 | 0.0036 |
| LOC_Os04g43800 | salicylic acid biosynthesis | phenylalanine ammonia-lyase, putative, expressed | 1.1939 | 0.0590 | -1.3904 | 0.6487 | 2.1294 | 0.0000 | 0.5175 | 0.6832 | -1.0063 | 0.2277 | 2.5192 | 0.0000 | 0.4738 | 0.6688 |
| LOC_Os05g35290 | salicylic acid biosynthesis | phenylalanine ammonia-lyase, putative, expressed | 0.8593 | 0.0042 | 0.2319 | 0.9207 | 0.1245 | 0.8110 | 0.5718 | 0.5404 | 0.7338 | 0.0086 | -0.4026 | 0.0442 | 1.0181 | 0.0117 |

logFC represents the log2 fold change. FDR represents the FDR-corrected p-value. S indicates the susceptible interaction and R indicates the resistant interaction, while the numbers indicate 3, 12, and 24 h post-inoculation time points.
