# Supplementary material for: RNA-Seq analysis reveals insight into enhanced rice Xa7-mediated bacterial blight resistance at high temperature
Source: PLoS One. 2017 Nov 6;12(11):e0187625. doi: 10.1371/journal.pone.0187625 (PMC5673197; doi:10.1371/journal.pone.0187625)
Supplement: S5 Table — (DOCX) [file pone.0187625.s007.docx]

**Table S5: Hormone response genes differentially expressed at high temperature.**

|  | **6 h** | **3 h** | | **12 h** | | **24 h** | |
| --- | --- | --- | --- | --- | --- | --- | --- |
|  | **Mock** | **Susceptible** | **Resistant** | **Susceptible** | **Resistant** | **Susceptible** | **Resistant** |
| **Total DEGs** | 1511 | 288 | 1463 | 2012 | 5985 | 795 | 2620 |
| **Auxin genes** | 21  1.4% | 6  2.1% | 15  1.0% | 21  1.0% | 44  0.7% | 11  1.4% | 24  0.9% |
| **Cytokinin genes** | 14  0.9% | 2  0.7% | 11  0.8% | 11  0.6% | 31  0.5% | 5  0.6% | 16  0.6% |
| **ABA genes** | 232  15.4% | 66  22.9% | 244  16.7% | 279  13.9% | 715  12.0% | 132  16.6% | 415  15.8% |
| **Ethylene genes** | 4  0.3% | 3  1.0% | 6  0.4% | 11  0.6% | 19  0.3% | 4  0.5% | 13  0.5% |
| **SA genes** | 23  1.5% | 9  3.1% | 29  2.0% | 46  2.3% | 106  1.8% | 21  2.6% | 66  2.5% |
| **JA genes** | 33  2.2% | 7  2.4% | 32  2.2% | 35  1.7% | 71  1.2% | 8  1.0% | 39  1.5% |

The whole numbers indicate the number of DEGs that are downstream of hormone response in each treatment, and the percentages indicate the percentage of total DEGs. Genes which were responsive to more than one hormone were equally divided among the hormones they were responsive to, with the total number of genes being rounded the nearest whole number.
