# Supplementary material for: RNA-Seq analysis reveals insight into enhanced rice Xa7-mediated bacterial blight resistance at high temperature
Source: PLoS One. 2017 Nov 6;12(11):e0187625. doi: 10.1371/journal.pone.0187625 (PMC5673197; doi:10.1371/journal.pone.0187625)
Supplement: S6 Table — (DOCX) [file pone.0187625.s008.docx]

**Table S6: Differential expression analysis of ABA-responsive genes.**

|  | **Mock** | **Susceptible Interaction** | | | **Resistant Interaction** | | |
| --- | --- | --- | --- | --- | --- | --- | --- |
| **Locus** | **6 hpi** | **3 hpi** | **12 hpi** | **24 hpi** | **3 hpi** | **12 hpi** | **24 hpi** |
| LOC_Os01g01620 | *n.s.* | 1.2204 | *n.s.* | *n.s.* | *n.s.* | 0.7899 | *n.s.* |
| LOC_Os01g02390 | *n.s.* | *n.s.* | *n.s.* | *n.s.* | 0.7036 | *n.s.* | *n.s.* |
| LOC_Os01g02400 | *n.s.* | *n.s.* | *n.s.* | *n.s.* | *n.s.* | 0.5059 | *n.s.* |
| LOC_Os01g03144 | *n.s.* | *n.s.* | *n.s.* | *n.s.* | *n.s.* | 0.6489 | *n.s.* |
| LOC_Os01g03390 | -3.1964 | *n.s.* | *n.s.* | *n.s.* | *n.s.* | -2.0837 | -3.1473 |
| LOC_Os01g03730 | *n.s.* | *n.s.* | *n.s.* | *n.s.* | *n.s.* | -0.9327 | -1.4372 |
| LOC_Os01g04330 | 2.195 | *n.s.* | *n.s.* | *n.s.* | *n.s.* | *n.s.* | *n.s.* |
| LOC_Os01g04590 | *n.s.* | *n.s.* | *n.s.* | *n.s.* | -0.9063 | *n.s.* | *n.s.* |
| LOC_Os01g05150 | *n.s.* | *n.s.* | *n.s.* | *n.s.* | *n.s.* | -1.7679 | *n.s.* |
| LOC_Os01g06920 | *n.s.* | *n.s.* | *n.s.* | *n.s.* | *n.s.* | -1.7016 | *n.s.* |
| LOC_Os01g07120 | 1.3255 | *n.s.* | *n.s.* | *n.s.* | -1.0307 | *n.s.* | *n.s.* |
| LOC_Os01g07200 | *n.s.* | *n.s.* | *n.s.* | *n.s.* | *n.s.* | -1.2737 | -1.0376 |
| LOC_Os01g07530 | *n.s.* | *n.s.* | *n.s.* | *n.s.* | *n.s.* | 0.5913 | *n.s.* |
| LOC_Os01g07700 | *n.s.* | *n.s.* | *n.s.* | *n.s.* | *n.s.* | 0.5402 | 0.6644 |
| LOC_Os01g07800 | *n.s.* | *n.s.* | *n.s.* | 0.8337 | *n.s.* | 1.3799 | 0.7277 |
| LOC_Os01g08470 | *n.s.* | *n.s.* | -0.7779 | *n.s.* | -1.2418 | -2.2164 | -2.3444 |
| LOC_Os01g08860 | *n.s.* | *n.s.* | 2.7673 | *n.s.* | *n.s.* | *n.s.* | 1.5399 |
| LOC_Os01g09020 | *n.s.* | *n.s.* | *n.s.* | *n.s.* | *n.s.* | -0.8677 | *n.s.* |
| LOC_Os01g09620 | *n.s.* | *n.s.* | *n.s.* | *n.s.* | 1.6586 | *n.s.* | *n.s.* |
| LOC_Os01g09640 | *n.s.* | 2.7146 | 1.2587 | *n.s.* | *n.s.* | *n.s.* | *n.s.* |
| LOC_Os01g09700 | 1.8504 | *n.s.* | *n.s.* | *n.s.* | *n.s.* | *n.s.* | *n.s.* |
| LOC_Os01g10320 | *n.s.* | 2.2536 | *n.s.* | *n.s.* | *n.s.* | *n.s.* | *n.s.* |
| LOC_Os01g10440 | 1.454 | *n.s.* | *n.s.* | *n.s.* | *n.s.* | *n.s.* | *n.s.* |
| LOC_Os01g10890 | *n.s.* | 1.921 | 1.0964 | *n.s.* | *n.s.* | 1.758 | *n.s.* |
| LOC_Os01g11150 | 1.4327 | *n.s.* | *n.s.* | *n.s.* | -1.2814 | -2.8476 | -1.8034 |
| LOC_Os01g11960 | *n.s.* | *n.s.* | *n.s.* | *n.s.* | *n.s.* | 0.6427 | *n.s.* |
| LOC_Os01g12160 | *n.s.* | *n.s.* | *n.s.* | *n.s.* | *n.s.* | 1.0808 | 0.9797 |
| LOC_Os01g12720 | 0.6328 | *n.s.* | *n.s.* | *n.s.* | *n.s.* | *n.s.* | *n.s.* |
| LOC_Os01g12820 | *n.s.* | *n.s.* | *n.s.* | *n.s.* | -1.0939 | -2.4277 | *n.s.* |
| LOC_Os01g12920 | *n.s.* | *n.s.* | 1.9146 | *n.s.* | *n.s.* | *n.s.* | *n.s.* |
| LOC_Os01g13210 | *n.s.* | 1.4982 | 0.8894 | *n.s.* | *n.s.* | 0.8289 | *n.s.* |
| LOC_Os01g13280 | *n.s.* | *n.s.* | *n.s.* | *n.s.* | *n.s.* | -0.9818 | -1.3226 |
| LOC_Os01g13950 | *n.s.* | *n.s.* | *n.s.* | *n.s.* | *n.s.* | -4.9125 | *n.s.* |
| LOC_Os01g14080 | 1.1307 | *n.s.* | *n.s.* | *n.s.* | *n.s.* | *n.s.* | *n.s.* |
| LOC_Os01g14510 | *n.s.* | *n.s.* | *n.s.* | *n.s.* | -1.1236 | *n.s.* | *n.s.* |
| LOC_Os01g15000 | 0.7187 | *n.s.* | *n.s.* | *n.s.* | *n.s.* | -1.1526 | -0.9402 |
| LOC_Os01g18220 | *n.s.* | *n.s.* | *n.s.* | *n.s.* | *n.s.* | -1.2546 | *n.s.* |
| LOC_Os01g19130 | *n.s.* | *n.s.* | *n.s.* | *n.s.* | -0.7085 | *n.s.* | *n.s.* |
| LOC_Os01g19800 | *n.s.* | *n.s.* | *n.s.* | *n.s.* | *n.s.* | *n.s.* | -1.495 |
| LOC_Os01g21420 | *n.s.* | *n.s.* | *n.s.* | *n.s.* | *n.s.* | *n.s.* | 0.6616 |
| LOC_Os01g22600 | *n.s.* | *n.s.* | *n.s.* | *n.s.* | -1.0158 | -0.9199 | -0.7408 |
| LOC_Os01g24480 | *n.s.* | *n.s.* | *n.s.* | *n.s.* | *n.s.* | 0.515 | *n.s.* |
| LOC_Os01g24710 | -2.8761 | *n.s.* | *n.s.* | *n.s.* | *n.s.* | *n.s.* | *n.s.* |
| LOC_Os01g26039 | *n.s.* | *n.s.* | 0.8033 | 0.8871 | *n.s.* | 1.2623 | *n.s.* |
| LOC_Os01g26120 | *n.s.* | *n.s.* | 0.8372 | *n.s.* | *n.s.* | *n.s.* | *n.s.* |
| LOC_Os01g26970 | *n.s.* | *n.s.* | *n.s.* | *n.s.* | *n.s.* | 1.5114 | *n.s.* |
| LOC_Os01g28840 | *n.s.* | *n.s.* | *n.s.* | -6.9188 | *n.s.* | -2.3203 | -6.9483 |
| LOC_Os01g32780 | *n.s.* | *n.s.* | -0.8908 | *n.s.* | *n.s.* | -1.5195 | -1.8631 |
| LOC_Os01g32980 | *n.s.* | *n.s.* | *n.s.* | *n.s.* | *n.s.* | -0.6659 | *n.s.* |
| LOC_Os01g33000 | *n.s.* | *n.s.* | *n.s.* | *n.s.* | *n.s.* | -0.5691 | *n.s.* |
| LOC_Os01g36790 | *n.s.* | *n.s.* | *n.s.* | *n.s.* | *n.s.* | -0.8412 | -1.6661 |
| LOC_Os01g37590 | *n.s.* | *n.s.* | 1.2315 | *n.s.* | *n.s.* | 1.5257 | *n.s.* |
| LOC_Os01g37750 | *n.s.* | *n.s.* | *n.s.* | *n.s.* | *n.s.* | -1.1908 | *n.s.* |
| LOC_Os01g37832 | *n.s.* | *n.s.* | *n.s.* | *n.s.* | *n.s.* | 0.8052 | *n.s.* |
| LOC_Os01g38990 | 1.2042 | *n.s.* | *n.s.* | *n.s.* | *n.s.* | *n.s.* | *n.s.* |
| LOC_Os01g40094 | *n.s.* | *n.s.* | *n.s.* | *n.s.* | -1.3457 | -0.6588 | -0.7842 |
| LOC_Os01g40280 | *n.s.* | *n.s.* | *n.s.* | -1.4391 | -1.1313 | -1.2053 | -1.774 |
| LOC_Os01g42190 | *n.s.* | *n.s.* | *n.s.* | -1.4645 | *n.s.* | *n.s.* | -2.3415 |
| LOC_Os01g42380 | *n.s.* | *n.s.* | *n.s.* | *n.s.* | *n.s.* | -1.5014 | -1.2703 |
| LOC_Os01g42850 | *n.s.* | *n.s.* | *n.s.* | *n.s.* | *n.s.* | 0.6395 | *n.s.* |
| LOC_Os01g43700 | -1.4154 | -1.8174 | -2.6308 | *n.s.* | -1.4416 | -1.2214 | *n.s.* |
| LOC_Os01g43851 | 1.2639 | *n.s.* | *n.s.* | *n.s.* | *n.s.* | -2.5195 | *n.s.* |
| LOC_Os01g45460 | *n.s.* | *n.s.* | *n.s.* | *n.s.* | *n.s.* | -1.6899 | -1.1964 |
| LOC_Os01g45659 | *n.s.* | 3.7876 | *n.s.* | *n.s.* | *n.s.* | *n.s.* | -6.5711 |
| LOC_Os01g45830 | *n.s.* | *n.s.* | *n.s.* | *n.s.* | *n.s.* | -1.2038 | -1.3775 |
| LOC_Os01g46970 | *n.s.* | *n.s.* | *n.s.* | *n.s.* | -1.4257 | -0.9639 | *n.s.* |
| LOC_Os01g47460 | *n.s.* | *n.s.* | -0.7066 | *n.s.* | *n.s.* | *n.s.* | *n.s.* |
| LOC_Os01g47490 | *n.s.* | 1.3034 | *n.s.* | *n.s.* | *n.s.* | *n.s.* | *n.s.* |
| LOC_Os01g47690 | *n.s.* | *n.s.* | -0.8464 | *n.s.* | 0.895 | -0.9117 | *n.s.* |
| LOC_Os01g47760 | *n.s.* | *n.s.* | -1.3272 | *n.s.* | *n.s.* | -1.8555 | *n.s.* |
| LOC_Os01g48446 | 1.3072 | *n.s.* | *n.s.* | *n.s.* | *n.s.* | *n.s.* | *n.s.* |
| LOC_Os01g48820 | *n.s.* | *n.s.* | *n.s.* | *n.s.* | *n.s.* | 1.0033 | *n.s.* |
| LOC_Os01g49280 | -1.6325 | *n.s.* | *n.s.* | *n.s.* | *n.s.* | -1.5358 | *n.s.* |
| LOC_Os01g49720 | *n.s.* | *n.s.* | *n.s.* | 1.4162 | *n.s.* | *n.s.* | *n.s.* |
| LOC_Os01g50030 | 1.2673 | *n.s.* | *n.s.* | *n.s.* | *n.s.* | -1.7499 | *n.s.* |
| LOC_Os01g50040 | *n.s.* | *n.s.* | *n.s.* | *n.s.* | *n.s.* | -1.0621 | *n.s.* |
| LOC_Os01g50050 | *n.s.* | *n.s.* | *n.s.* | *n.s.* | -1.1406 | *n.s.* | *n.s.* |
| LOC_Os01g50400 | *n.s.* | 2.2137 | *n.s.* | *n.s.* | *n.s.* | *n.s.* | -1.9288 |
| LOC_Os01g50616 | *n.s.* | *n.s.* | *n.s.* | *n.s.* | *n.s.* | -1.3648 | -0.9992 |
| LOC_Os01g51380 | *n.s.* | *n.s.* | *n.s.* | *n.s.* | -0.6549 | -0.7926 | *n.s.* |
| LOC_Os01g52030 | *n.s.* | *n.s.* | 0.918 | 1.2893 | 1.478 | *n.s.* | *n.s.* |
| LOC_Os01g52110 | *n.s.* | *n.s.* | *n.s.* | *n.s.* | *n.s.* | 0.7166 | 0.8937 |
| LOC_Os01g52130 | *n.s.* | 2.051 | *n.s.* | *n.s.* | *n.s.* | *n.s.* | *n.s.* |
| LOC_Os01g52730 | *n.s.* | *n.s.* | *n.s.* | *n.s.* | *n.s.* | -1.9734 | *n.s.* |
| LOC_Os01g52980 | *n.s.* | *n.s.* | *n.s.* | *n.s.* | *n.s.* | -1.0948 | *n.s.* |
| LOC_Os01g53650 | *n.s.* | *n.s.* | *n.s.* | *n.s.* | *n.s.* | *n.s.* | -5.6202 |
| LOC_Os01g55160 | *n.s.* | *n.s.* | -1.7409 | *n.s.* | *n.s.* | -1.8758 | *n.s.* |
| LOC_Os01g55340 | *n.s.* | *n.s.* | *n.s.* | *n.s.* | *n.s.* | *n.s.* | -2.8152 |
| LOC_Os01g55450 | 0.8147 | *n.s.* | *n.s.* | 1.0644 | *n.s.* | *n.s.* | 1.1039 |
| LOC_Os01g56070 | *n.s.* | *n.s.* | *n.s.* | *n.s.* | *n.s.* | -0.8138 | *n.s.* |
| LOC_Os01g56180 | *n.s.* | *n.s.* | -1.5333 | *n.s.* | -1.615 | -2.0162 | *n.s.* |
| LOC_Os01g57420 | -0.6576 | *n.s.* | *n.s.* | *n.s.* | *n.s.* | *n.s.* | *n.s.* |
| LOC_Os01g57450 | *n.s.* | *n.s.* | *n.s.* | *n.s.* | *n.s.* | -0.8841 | -1.0217 |
| LOC_Os01g58194 | *n.s.* | *n.s.* | *n.s.* | *n.s.* | *n.s.* | -0.7015 | *n.s.* |
| LOC_Os01g58420 | 0.8964 | *n.s.* | *n.s.* | *n.s.* | *n.s.* | -0.6193 | *n.s.* |
| LOC_Os01g59000 | *n.s.* | -2.29 | *n.s.* | *n.s.* | *n.s.* | -1.53 | -1.8102 |
| LOC_Os01g59100 | *n.s.* | *n.s.* | -1.2629 | *n.s.* | *n.s.* | -0.739 | *n.s.* |
| LOC_Os01g59970 | *n.s.* | *n.s.* | -0.5293 | *n.s.* | *n.s.* | *n.s.* | *n.s.* |
| LOC_Os01g60309 | *n.s.* | *n.s.* | *n.s.* | *n.s.* | -0.6602 | -0.6476 | *n.s.* |
| LOC_Os01g60730 | 0.8928 | *n.s.* | *n.s.* | *n.s.* | *n.s.* | -1.2898 | *n.s.* |
| LOC_Os01g60910 | *n.s.* | *n.s.* | *n.s.* | *n.s.* | *n.s.* | -0.6301 | *n.s.* |
| LOC_Os01g61410 | *n.s.* | *n.s.* | *n.s.* | *n.s.* | *n.s.* | 0.5767 | *n.s.* |
| LOC_Os01g61670 | *n.s.* | *n.s.* | *n.s.* | *n.s.* | *n.s.* | 0.521 | *n.s.* |
| LOC_Os01g61680 | 1.4804 | 1.0964 | *n.s.* | *n.s.* | -0.7972 | -0.8198 | *n.s.* |
| LOC_Os01g62190 | *n.s.* | *n.s.* | *n.s.* | *n.s.* | *n.s.* | -1.7322 | *n.s.* |
| LOC_Os01g62610 | -2.434 | *n.s.* | *n.s.* | *n.s.* | *n.s.* | *n.s.* | -1.2369 |
| LOC_Os01g62760 | *n.s.* | 1.4347 | *n.s.* | *n.s.* | -1.0217 | -1.0849 | *n.s.* |
| LOC_Os01g62780 | *n.s.* | *n.s.* | *n.s.* | *n.s.* | -1.231 | -0.8286 | *n.s.* |
| LOC_Os01g62810 | *n.s.* | *n.s.* | -1.5058 | *n.s.* | -1.4791 | -2.1004 | *n.s.* |
| LOC_Os01g62970 | *n.s.* | *n.s.* | *n.s.* | *n.s.* | *n.s.* | -1.0499 | *n.s.* |
| LOC_Os01g62980 | *n.s.* | 1.1885 | *n.s.* | *n.s.* | *n.s.* | *n.s.* | *n.s.* |
| LOC_Os01g63010 | *n.s.* | *n.s.* | 0.8327 | *n.s.* | *n.s.* | *n.s.* | *n.s.* |
| LOC_Os01g63060 | *n.s.* | *n.s.* | -0.6641 | *n.s.* | -1.3574 | -1.3716 | -0.7261 |
| LOC_Os01g63230 | *n.s.* | *n.s.* | *n.s.* | *n.s.* | *n.s.* | *n.s.* | 1.4886 |
| LOC_Os01g63480 | *n.s.* | *n.s.* | 0.973 | *n.s.* | *n.s.* | *n.s.* | *n.s.* |
| LOC_Os01g63930 | 1.3512 | *n.s.* | *n.s.* | *n.s.* | *n.s.* | *n.s.* | *n.s.* |
| LOC_Os01g63980 | *n.s.* | *n.s.* | *n.s.* | *n.s.* | *n.s.* | -1.0668 | *n.s.* |
| LOC_Os01g64300 | *n.s.* | *n.s.* | *n.s.* | *n.s.* | *n.s.* | *n.s.* | -1.355 |
| LOC_Os01g64360 | 1.4725 | *n.s.* | *n.s.* | *n.s.* | -1.4777 | -1.4031 | *n.s.* |
| LOC_Os01g64730 | *n.s.* | *n.s.* | *n.s.* | *n.s.* | *n.s.* | -0.7598 | -1.6696 |
| LOC_Os01g64750 | 0.7023 | *n.s.* | *n.s.* | *n.s.* | *n.s.* | *n.s.* | *n.s.* |
| LOC_Os01g65520 | 0.9347 | *n.s.* | -0.7376 | *n.s.* | -1.659 | -1.3072 | *n.s.* |
| LOC_Os01g65670 | *n.s.* | *n.s.* | -1.8797 | *n.s.* | -1.4954 | -2.7764 | *n.s.* |
| LOC_Os01g66120 | *n.s.* | *n.s.* | -0.7285 | *n.s.* | *n.s.* | -1.3683 | *n.s.* |
| LOC_Os01g66240 | *n.s.* | *n.s.* | *n.s.* | *n.s.* | *n.s.* | -0.917 | *n.s.* |
| LOC_Os01g67420 | *n.s.* | *n.s.* | -0.5856 | *n.s.* | *n.s.* | -0.6188 | *n.s.* |
| LOC_Os01g67870 | *n.s.* | *n.s.* | *n.s.* | *n.s.* | -0.8556 | -0.7527 | *n.s.* |
| LOC_Os01g68570 | *n.s.* | *n.s.* | -1.2109 | -2.1697 | *n.s.* | *n.s.* | -1.8878 |
| LOC_Os01g70550 | *n.s.* | *n.s.* | 1.4189 | *n.s.* | *n.s.* | 1.6943 | 0.6732 |
| LOC_Os01g70860 | *n.s.* | *n.s.* | *n.s.* | *n.s.* | *n.s.* | -0.9345 | *n.s.* |
| LOC_Os01g70950 | *n.s.* | *n.s.* | 0.511 | *n.s.* | *n.s.* | 0.7154 | *n.s.* |
| LOC_Os01g71690 | *n.s.* | *n.s.* | *n.s.* | *n.s.* | *n.s.* | -1.0596 | -0.9452 |
| LOC_Os01g72520 | 0.8975 | *n.s.* | *n.s.* | *n.s.* | *n.s.* | *n.s.* | *n.s.* |
| LOC_Os01g72570 | *n.s.* | *n.s.* | *n.s.* | *n.s.* | *n.s.* | -0.7626 | -0.8206 |
| LOC_Os01g72610 | 0.845 | *n.s.* | *n.s.* | 1.046 | *n.s.* | -0.6534 | *n.s.* |
| LOC_Os01g72970 | *n.s.* | *n.s.* | *n.s.* | *n.s.* | -0.88 | -1.7992 | -1.1865 |
| LOC_Os01g73110 | -2.5434 | *n.s.* | *n.s.* | *n.s.* | -2.4876 | *n.s.* | *n.s.* |
| LOC_Os02g01700 | *n.s.* | *n.s.* | 1.391 | *n.s.* | *n.s.* | *n.s.* | *n.s.* |
| LOC_Os02g04630 | *n.s.* | *n.s.* | -1.7952 | *n.s.* | -2.6567 | -1.6923 | *n.s.* |
| LOC_Os02g04840 | *n.s.* | *n.s.* | 0.4766 | *n.s.* | *n.s.* | 0.9458 | *n.s.* |
| LOC_Os02g06640 | *n.s.* | *n.s.* | *n.s.* | *n.s.* | *n.s.* | -0.7332 | *n.s.* |
| LOC_Os02g06950 | *n.s.* | 3.7659 | *n.s.* | *n.s.* | *n.s.* | *n.s.* | *n.s.* |
| LOC_Os02g07690 | 0.9871 | *n.s.* | *n.s.* | *n.s.* | *n.s.* | -1.1548 | *n.s.* |
| LOC_Os02g07930 | *n.s.* | *n.s.* | *n.s.* | 1.1474 | 1.0701 | -1.8253 | 0.9061 |
| LOC_Os02g09480 | *n.s.* | *n.s.* | -1.338 | *n.s.* | *n.s.* | *n.s.* | *n.s.* |
| LOC_Os02g09810 | *n.s.* | *n.s.* | *n.s.* | *n.s.* | *n.s.* | -0.6782 | -2.577 |
| LOC_Os02g09830 | *n.s.* | *n.s.* | *n.s.* | *n.s.* | *n.s.* | -1.3891 | *n.s.* |
| LOC_Os02g10070 | *n.s.* | *n.s.* | -0.4951 | *n.s.* | *n.s.* | *n.s.* | *n.s.* |
| LOC_Os02g10630 | *n.s.* | *n.s.* | *n.s.* | *n.s.* | *n.s.* | 1.0614 | *n.s.* |
| LOC_Os02g10730 | 0.9722 | *n.s.* | -0.7603 | *n.s.* | *n.s.* | -1.4179 | *n.s.* |
| LOC_Os02g10780 | *n.s.* | *n.s.* | -0.8724 | 1.7912 | 0.8866 | *n.s.* | 1.6222 |
| LOC_Os02g11760 | *n.s.* | *n.s.* | *n.s.* | -1.267 | *n.s.* | -1.1416 | -1.5671 |
| LOC_Os02g12350 | *n.s.* | *n.s.* | *n.s.* | *n.s.* | *n.s.* | -1.8315 | *n.s.* |
| LOC_Os02g12420 | *n.s.* | *n.s.* | *n.s.* | -1.5968 | *n.s.* | *n.s.* | *n.s.* |
| LOC_Os02g12670 | *n.s.* | *n.s.* | *n.s.* | *n.s.* | *n.s.* | -0.8981 | *n.s.* |
| LOC_Os02g13800 | *n.s.* | 2.2716 | *n.s.* | *n.s.* | *n.s.* | -1.0983 | *n.s.* |
| LOC_Os02g15870 | -0.822 | *n.s.* | *n.s.* | *n.s.* | *n.s.* | *n.s.* | *n.s.* |
| LOC_Os02g21920 | 0.9634 | *n.s.* | *n.s.* | *n.s.* | *n.s.* | -2.0468 | -1.19 |
| LOC_Os02g22160 | 2.5965 | *n.s.* | *n.s.* | *n.s.* | *n.s.* | -3.5942 | *n.s.* |
| LOC_Os02g26720 | *n.s.* | *n.s.* | 1.7536 | *n.s.* | *n.s.* | *n.s.* | -1.7019 |
| LOC_Os02g27400 | *n.s.* | *n.s.* | 0.5757 | *n.s.* | *n.s.* | 0.6434 | *n.s.* |
| LOC_Os02g30910 | *n.s.* | *n.s.* | *n.s.* | -5.277 | *n.s.* | -3.6739 | -5.9935 |
| LOC_Os02g32140 | *n.s.* | *n.s.* | *n.s.* | *n.s.* | *n.s.* | -1.4313 | -1.0769 |
| LOC_Os02g32450 | *n.s.* | *n.s.* | *n.s.* | *n.s.* | 1.258 | *n.s.* | *n.s.* |
| LOC_Os02g32520 | 1.676 | *n.s.* | -0.997 | *n.s.* | -1.7025 | -2.2617 | -1.776 |
| LOC_Os02g32580 | 1.0768 | 2.4186 | 1.067 | 0.8987 | *n.s.* | *n.s.* | *n.s.* |
| LOC_Os02g32590 | *n.s.* | *n.s.* | *n.s.* | *n.s.* | -0.7905 | -1.1909 | *n.s.* |
| LOC_Os02g35190 | *n.s.* | *n.s.* | *n.s.* | *n.s.* | *n.s.* | 0.7234 | *n.s.* |
| LOC_Os02g35590 | *n.s.* | *n.s.* | *n.s.* | *n.s.* | *n.s.* | 0.7184 | *n.s.* |
| LOC_Os02g35820 | *n.s.* | *n.s.* | *n.s.* | *n.s.* | *n.s.* | *n.s.* | 0.8529 |
| LOC_Os02g35830 | *n.s.* | *n.s.* | *n.s.* | *n.s.* | *n.s.* | -1.0014 | *n.s.* |
| LOC_Os02g35870 | 1.1775 | *n.s.* | -0.7835 | *n.s.* | -0.8971 | -0.9679 | *n.s.* |
| LOC_Os02g36490 | *n.s.* | *n.s.* | *n.s.* | *n.s.* | *n.s.* | -0.9443 | *n.s.* |
| LOC_Os02g37610 | -0.8003 | *n.s.* | *n.s.* | *n.s.* | *n.s.* | *n.s.* | *n.s.* |
| LOC_Os02g37880 | *n.s.* | *n.s.* | -0.5461 | *n.s.* | *n.s.* | *n.s.* | *n.s.* |
| LOC_Os02g39790 | *n.s.* | *n.s.* | -0.7416 | *n.s.* | *n.s.* | -0.6862 | *n.s.* |
| LOC_Os02g39930 | *n.s.* | *n.s.* | *n.s.* | *n.s.* | *n.s.* | -0.6925 | -1.5567 |
| LOC_Os02g40500 | *n.s.* | *n.s.* | *n.s.* | *n.s.* | *n.s.* | -1.153 | *n.s.* |
| LOC_Os02g42020 | *n.s.* | *n.s.* | -0.7086 | *n.s.* | *n.s.* | -0.9293 | *n.s.* |
| LOC_Os02g42220 | -0.6593 | *n.s.* | *n.s.* | *n.s.* | *n.s.* | *n.s.* | *n.s.* |
| LOC_Os02g42940 | *n.s.* | *n.s.* | *n.s.* | *n.s.* | *n.s.* | -0.5396 | *n.s.* |
| LOC_Os02g43330 | *n.s.* | 3.5259 | *n.s.* | -5.4619 | *n.s.* | -3.8751 | -5.9295 |
| LOC_Os02g44770 | *n.s.* | *n.s.* | *n.s.* | *n.s.* | *n.s.* | -1.9366 | *n.s.* |
| LOC_Os02g44870 | *n.s.* | *n.s.* | *n.s.* | *n.s.* | -1.2827 | -1.4125 | -1.9787 |
| LOC_Os02g44990 | *n.s.* | *n.s.* | *n.s.* | *n.s.* | *n.s.* | -1.5715 | -1.6276 |
| LOC_Os02g45690 | *n.s.* | *n.s.* | *n.s.* | *n.s.* | *n.s.* | 1.7647 | *n.s.* |
| LOC_Os02g46030 | *n.s.* | *n.s.* | *n.s.* | -2.0208 | *n.s.* | *n.s.* | -2.4303 |
| LOC_Os02g46640 | -0.9994 | *n.s.* | *n.s.* | -0.8836 | *n.s.* | 0.7056 | -0.8662 |
| LOC_Os02g47200 | *n.s.* | *n.s.* | *n.s.* | 1.1629 | *n.s.* | *n.s.* | *n.s.* |
| LOC_Os02g47470 | 1.8543 | *n.s.* | *n.s.* | *n.s.* | *n.s.* | -1.7853 | *n.s.* |
| LOC_Os02g47560 | 0.7436 | *n.s.* | *n.s.* | 1.2914 | *n.s.* | *n.s.* | 1.5313 |
| LOC_Os02g47650 | 1.0695 | *n.s.* | *n.s.* | *n.s.* | *n.s.* | *n.s.* | *n.s.* |
| LOC_Os02g48170 | *n.s.* | *n.s.* | *n.s.* | *n.s.* | *n.s.* | -1.1014 | *n.s.* |
| LOC_Os02g48190 | *n.s.* | *n.s.* | *n.s.* | *n.s.* | -1.5654 | -1.4669 | -2.5907 |
| LOC_Os02g48630 | *n.s.* | *n.s.* | -0.5871 | *n.s.* | *n.s.* | -1.216 | *n.s.* |
| LOC_Os02g48710 | 2.0543 | *n.s.* | *n.s.* | *n.s.* | -0.7624 | -1.9687 | -1.5021 |
| LOC_Os02g48740 | *n.s.* | *n.s.* | *n.s.* | *n.s.* | *n.s.* | -0.7515 | *n.s.* |
| LOC_Os02g50350 | *n.s.* | *n.s.* | *n.s.* | *n.s.* | *n.s.* | *n.s.* | -1.3126 |
| LOC_Os02g50690 | *n.s.* | -1.8885 | *n.s.* | *n.s.* | *n.s.* | -2.11 | -3.1802 |
| LOC_Os02g50730 | *n.s.* | *n.s.* | *n.s.* | *n.s.* | *n.s.* | -1.0301 | *n.s.* |
| LOC_Os02g50805 | *n.s.* | *n.s.* | *n.s.* | *n.s.* | *n.s.* | -1.5449 | *n.s.* |
| LOC_Os02g51350 | 2.0409 | *n.s.* | *n.s.* | *n.s.* | -1.3538 | -1.9578 | -1.7168 |
| LOC_Os02g51540 | *n.s.* | *n.s.* | *n.s.* | *n.s.* | *n.s.* | -1.2872 | *n.s.* |
| LOC_Os02g51890 | *n.s.* | *n.s.* | *n.s.* | *n.s.* | *n.s.* | -1.3479 | *n.s.* |
| LOC_Os02g52780 | *n.s.* | *n.s.* | *n.s.* | *n.s.* | -1.0007 | -0.5227 | -0.6723 |
| LOC_Os02g53994 | *n.s.* | *n.s.* | *n.s.* | *n.s.* | *n.s.* | 0.7218 | *n.s.* |
| LOC_Os02g54240 | *n.s.* | *n.s.* | *n.s.* | *n.s.* | *n.s.* | -1.3639 | -5.2911 |
| LOC_Os02g54254 | *n.s.* | *n.s.* | *n.s.* | *n.s.* | *n.s.* | -1.3899 | -2.0508 |
| LOC_Os02g54780 | 0.8349 | *n.s.* | *n.s.* | *n.s.* | *n.s.* | *n.s.* | *n.s.* |
| LOC_Os02g56310 | *n.s.* | *n.s.* | *n.s.* | *n.s.* | *n.s.* | *n.s.* | 1.0885 |
| LOC_Os02g57840 | *n.s.* | 4.9935 | *n.s.* | *n.s.* | *n.s.* | *n.s.* | *n.s.* |
| LOC_Os02g58150 | *n.s.* | *n.s.* | -0.7518 | *n.s.* | *n.s.* | -0.7994 | *n.s.* |
| LOC_Os02g58160 | *n.s.* | *n.s.* | *n.s.* | *n.s.* | *n.s.* | 1.0441 | *n.s.* |
| LOC_Os03g01350 | *n.s.* | *n.s.* | *n.s.* | *n.s.* | *n.s.* | 0.9676 | 0.8594 |
| LOC_Os03g02874 | *n.s.* | *n.s.* | -0.7654 | *n.s.* | *n.s.* | -2.0183 | -1.7534 |
| LOC_Os03g03370 | 1.7543 | 1.5136 | *n.s.* | *n.s.* | -1.1467 | -1.8527 | *n.s.* |
| LOC_Os03g03810 | -2.2185 | *n.s.* | 1.2423 | *n.s.* | *n.s.* | *n.s.* | *n.s.* |
| LOC_Os03g04130 | *n.s.* | *n.s.* | 1.7997 | *n.s.* | 1.8658 | 2.2448 | *n.s.* |
| LOC_Os03g04370 | 1.7648 | *n.s.* | *n.s.* | *n.s.* | *n.s.* | *n.s.* | *n.s.* |
| LOC_Os03g04410 | *n.s.* | *n.s.* | *n.s.* | *n.s.* | *n.s.* | -0.6185 | *n.s.* |
| LOC_Os03g04890 | *n.s.* | *n.s.* | *n.s.* | *n.s.* | -1.3978 | -1.7527 | *n.s.* |
| LOC_Os03g04920 | *n.s.* | *n.s.* | *n.s.* | *n.s.* | -0.8962 | -0.7567 | -0.7909 |
| LOC_Os03g05280 | *n.s.* | *n.s.* | *n.s.* | *n.s.* | *n.s.* | -0.7349 | *n.s.* |
| LOC_Os03g05310 | *n.s.* | *n.s.* | *n.s.* | *n.s.* | *n.s.* | -0.7097 | *n.s.* |
| LOC_Os03g05910 | 0.9298 | *n.s.* | -1.6819 | *n.s.* | 1.3359 | *n.s.* | *n.s.* |
| LOC_Os03g06250 | *n.s.* | *n.s.* | *n.s.* | *n.s.* | *n.s.* | -1.1504 | -1.3342 |
| LOC_Os03g06350 | *n.s.* | *n.s.* | *n.s.* | *n.s.* | *n.s.* | 1.1736 | *n.s.* |
| LOC_Os03g06520 | *n.s.* | 1.4308 | *n.s.* | *n.s.* | *n.s.* | *n.s.* | *n.s.* |
| LOC_Os03g07190 | *n.s.* | *n.s.* | *n.s.* | *n.s.* | *n.s.* | -1.5614 | *n.s.* |
| LOC_Os03g08200 | *n.s.* | *n.s.* | *n.s.* | *n.s.* | *n.s.* | *n.s.* | -3.1149 |
| LOC_Os03g08320 | *n.s.* | *n.s.* | *n.s.* | *n.s.* | *n.s.* | -1.2946 | -1.3039 |
| LOC_Os03g08390 | *n.s.* | *n.s.* | *n.s.* | *n.s.* | *n.s.* | 0.7187 | *n.s.* |
| LOC_Os03g08490 | *n.s.* | *n.s.* | -1.8537 | *n.s.* | *n.s.* | *n.s.* | -2.2194 |
| LOC_Os03g08550 | 1.4544 | *n.s.* | *n.s.* | *n.s.* | -0.8518 | *n.s.* | -1.0308 |
| LOC_Os03g08900 | *n.s.* | *n.s.* | -1.3606 | *n.s.* | -1.7313 | -1.6307 | *n.s.* |
| LOC_Os03g09170 | 3.608 | *n.s.* | *n.s.* | *n.s.* | -2.2395 | -3.2831 | -2.7149 |
| LOC_Os03g09250 | 1.07 | *n.s.* | *n.s.* | *n.s.* | *n.s.* | *n.s.* | *n.s.* |
| LOC_Os03g10080 | *n.s.* | *n.s.* | *n.s.* | *n.s.* | *n.s.* | 0.8474 | 0.8749 |
| LOC_Os03g10090 | *n.s.* | *n.s.* | 1.5869 | 1.0121 | *n.s.* | 0.9233 | 1.5979 |
| LOC_Os03g10100 | *n.s.* | *n.s.* | *n.s.* | *n.s.* | *n.s.* | -1.8576 | *n.s.* |
| LOC_Os03g10320 | *n.s.* | *n.s.* | *n.s.* | *n.s.* | *n.s.* | -0.9859 | -1.3046 |
| LOC_Os03g11420 | *n.s.* | *n.s.* | *n.s.* | *n.s.* | *n.s.* | *n.s.* | -1.6949 |
| LOC_Os03g11490 | *n.s.* | *n.s.* | *n.s.* | *n.s.* | *n.s.* | 0.6764 | *n.s.* |
| LOC_Os03g11550 | *n.s.* | *n.s.* | *n.s.* | *n.s.* | *n.s.* | -0.5654 | *n.s.* |
| LOC_Os03g12940 | *n.s.* | *n.s.* | *n.s.* | *n.s.* | *n.s.* | 0.5046 | *n.s.* |
| LOC_Os03g13300 | *n.s.* | *n.s.* | 1.5298 | *n.s.* | -1.0161 | *n.s.* | -2.1681 |
| LOC_Os03g13820 | *n.s.* | *n.s.* | 0.7982 | *n.s.* | *n.s.* | *n.s.* | *n.s.* |
| LOC_Os03g13840 | 1.0677 | *n.s.* | *n.s.* | *n.s.* | -0.978 | -1.7505 | *n.s.* |
| LOC_Os03g14370 | 1.0339 | *n.s.* | *n.s.* | *n.s.* | -1.0769 | -1.9725 | -0.9847 |
| LOC_Os03g14420 | *n.s.* | *n.s.* | *n.s.* | *n.s.* | *n.s.* | 1.518 | *n.s.* |
| LOC_Os03g16170 | 1.1963 | *n.s.* | *n.s.* | -1.1553 | -1.5725 | -1.9945 | -3.0009 |
| LOC_Os03g16334 | *n.s.* | *n.s.* | *n.s.* | *n.s.* | -1.1805 | -1.0256 | -1.243 |
| LOC_Os03g16940 | -5.7405 | *n.s.* | *n.s.* | *n.s.* | *n.s.* | *n.s.* | *n.s.* |
| LOC_Os03g17480 | *n.s.* | *n.s.* | *n.s.* | 2.645 | *n.s.* | *n.s.* | 2.8928 |
| LOC_Os03g17790 | *n.s.* | *n.s.* | *n.s.* | -3.7045 | *n.s.* | *n.s.* | -5.5119 |
| LOC_Os03g18150 | 1.3411 | *n.s.* | *n.s.* | *n.s.* | -1.2319 | -1.0513 | *n.s.* |
| LOC_Os03g18490 | *n.s.* | *n.s.* | *n.s.* | *n.s.* | *n.s.* | *n.s.* | -2.6754 |
| LOC_Os03g19090 | *n.s.* | 1.6841 | *n.s.* | *n.s.* | *n.s.* | *n.s.* | *n.s.* |
| LOC_Os03g19250 | *n.s.* | *n.s.* | *n.s.* | *n.s.* | 1.2036 | *n.s.* | *n.s.* |
| LOC_Os03g19290 | *n.s.* | *n.s.* | *n.s.* | *n.s.* | *n.s.* | *n.s.* | -2.3524 |
| LOC_Os03g20020 | *n.s.* | *n.s.* | *n.s.* | *n.s.* | *n.s.* | -0.5738 | *n.s.* |
| LOC_Os03g20120 | *n.s.* | *n.s.* | *n.s.* | *n.s.* | *n.s.* | *n.s.* | 1.3771 |
| LOC_Os03g20680 | *n.s.* | 6.37 | *n.s.* | *n.s.* | *n.s.* | *n.s.* | *n.s.* |
| LOC_Os03g20870 | *n.s.* | *n.s.* | 0.7446 | *n.s.* | *n.s.* | *n.s.* | *n.s.* |
| LOC_Os03g21030 | 0.9334 | *n.s.* | *n.s.* | *n.s.* | -1.1235 | -1.2069 | -1.2689 |
| LOC_Os03g21040 | -1.0898 | *n.s.* | *n.s.* | *n.s.* | *n.s.* | *n.s.* | -1.1395 |
| LOC_Os03g21060 | *n.s.* | *n.s.* | *n.s.* | *n.s.* | *n.s.* | -1.2663 | *n.s.* |
| LOC_Os03g21850 | *n.s.* | *n.s.* | *n.s.* | *n.s.* | *n.s.* | -0.8609 | *n.s.* |
| LOC_Os03g22120 | *n.s.* | *n.s.* | *n.s.* | *n.s.* | *n.s.* | *n.s.* | -0.7254 |
| LOC_Os03g22200 | *n.s.* | *n.s.* | 0.9857 | *n.s.* | *n.s.* | *n.s.* | *n.s.* |
| LOC_Os03g22680 | *n.s.* | *n.s.* | *n.s.* | *n.s.* | *n.s.* | *n.s.* | -2.2711 |
| LOC_Os03g22790 | *n.s.* | *n.s.* | *n.s.* | *n.s.* | *n.s.* | *n.s.* | -1.8929 |
| LOC_Os03g25790 | *n.s.* | *n.s.* | -0.9174 | -1.6651 | *n.s.* | -1.9439 | -2.1677 |
| LOC_Os03g26910 | *n.s.* | *n.s.* | *n.s.* | *n.s.* | -1.487 | -1.157 | *n.s.* |
| LOC_Os03g27110 | *n.s.* | 1.9048 | *n.s.* | *n.s.* | *n.s.* | -2.0577 | *n.s.* |
| LOC_Os03g27280 | *n.s.* | *n.s.* | *n.s.* | *n.s.* | *n.s.* | -1.1225 | *n.s.* |
| LOC_Os03g28090 | -3.6323 | *n.s.* | *n.s.* | *n.s.* | *n.s.* | *n.s.* | *n.s.* |
| LOC_Os03g28300 | 0.6614 | *n.s.* | 1.1379 | 0.9655 | *n.s.* | 1.3271 | 1.2393 |
| LOC_Os03g29960 | *n.s.* | *n.s.* | *n.s.* | *n.s.* | *n.s.* | 0.6395 | *n.s.* |
| LOC_Os03g31180 | *n.s.* | *n.s.* | *n.s.* | *n.s.* | *n.s.* | *n.s.* | 0.6917 |
| LOC_Os03g31510 | *n.s.* | *n.s.* | -1.7909 | *n.s.* | -1.1935 | -0.8713 | -1.4753 |
| LOC_Os03g31750 | *n.s.* | *n.s.* | -1.5139 | *n.s.* | *n.s.* | *n.s.* | *n.s.* |
| LOC_Os03g32420 | 1.8742 | *n.s.* | *n.s.* | *n.s.* | *n.s.* | -1.5244 | *n.s.* |
| LOC_Os03g37490 | *n.s.* | *n.s.* | *n.s.* | *n.s.* | *n.s.* | -1.0521 | *n.s.* |
| LOC_Os03g43720 | *n.s.* | *n.s.* | *n.s.* | *n.s.* | *n.s.* | *n.s.* | -0.8477 |
| LOC_Os03g44150 | *n.s.* | *n.s.* | *n.s.* | *n.s.* | *n.s.* | -0.6838 | -0.9214 |
| LOC_Os03g45210 | *n.s.* | *n.s.* | *n.s.* | *n.s.* | *n.s.* | *n.s.* | -3.074 |
| LOC_Os03g46060 | *n.s.* | *n.s.* | -1.7278 | *n.s.* | *n.s.* | -2.2366 | -3.1316 |
| LOC_Os03g48310 | *n.s.* | *n.s.* | *n.s.* | *n.s.* | *n.s.* | -0.524 | -1.4087 |
| LOC_Os03g48660 | *n.s.* | *n.s.* | *n.s.* | *n.s.* | -1.169 | -0.6263 | -0.7988 |
| LOC_Os03g48970 | *n.s.* | *n.s.* | *n.s.* | *n.s.* | *n.s.* | *n.s.* | 0.6668 |
| LOC_Os03g49190 | *n.s.* | *n.s.* | *n.s.* | *n.s.* | *n.s.* | -5.3238 | *n.s.* |
| LOC_Os03g50960 | *n.s.* | *n.s.* | *n.s.* | *n.s.* | *n.s.* | -1.7321 | *n.s.* |
| LOC_Os03g51330 | *n.s.* | *n.s.* | *n.s.* | *n.s.* | *n.s.* | -1.254 | *n.s.* |
| LOC_Os03g51350 | *n.s.* | 3.1287 | *n.s.* | *n.s.* | *n.s.* | *n.s.* | -8.1491 |
| LOC_Os03g51390 | *n.s.* | *n.s.* | *n.s.* | *n.s.* | *n.s.* | -1.2702 | *n.s.* |
| LOC_Os03g51920 | *n.s.* | *n.s.* | *n.s.* | *n.s.* | *n.s.* | -1.6663 | *n.s.* |
| LOC_Os03g52380 | *n.s.* | *n.s.* | *n.s.* | *n.s.* | *n.s.* | -1.4124 | -2.1773 |
| LOC_Os03g54050 | *n.s.* | *n.s.* | *n.s.* | *n.s.* | *n.s.* | *n.s.* | -1.506 |
| LOC_Os03g55100 | *n.s.* | *n.s.* | *n.s.* | *n.s.* | *n.s.* | *n.s.* | -0.9321 |
| LOC_Os03g55240 | *n.s.* | *n.s.* | *n.s.* | 1.2609 | *n.s.* | *n.s.* | 1.3641 |
| LOC_Os03g55290 | *n.s.* | *n.s.* | *n.s.* | *n.s.* | *n.s.* | *n.s.* | -4.0542 |
| LOC_Os03g55410 | *n.s.* | *n.s.* | *n.s.* | *n.s.* | *n.s.* | 1.0747 | 1.3073 |
| LOC_Os03g55870 | 0.5916 | *n.s.* | *n.s.* | *n.s.* | *n.s.* | *n.s.* | *n.s.* |
| LOC_Os03g55990 | *n.s.* | *n.s.* | *n.s.* | *n.s.* | *n.s.* | 0.5794 | *n.s.* |
| LOC_Os03g56060 | *n.s.* | *n.s.* | *n.s.* | *n.s.* | *n.s.* | *n.s.* | -2.0478 |
| LOC_Os03g56250 | 1.2022 | *n.s.* | *n.s.* | *n.s.* | *n.s.* | -0.6312 | *n.s.* |
| LOC_Os03g57200 | *n.s.* | *n.s.* | -2.2507 | 2.7186 | *n.s.* | -2.6361 | 1.9592 |
| LOC_Os03g57880 | 2.0995 | *n.s.* | *n.s.* | *n.s.* | *n.s.* | -1.6774 | -2.1888 |
| LOC_Os03g57970 | *n.s.* | *n.s.* | 6.4633 | *n.s.* | *n.s.* | *n.s.* | *n.s.* |
| LOC_Os03g59040 | 1.0881 | *n.s.* | *n.s.* | *n.s.* | *n.s.* | *n.s.* | *n.s.* |
| LOC_Os03g59110 | *n.s.* | *n.s.* | *n.s.* | *n.s.* | *n.s.* | 1.4855 | *n.s.* |
| LOC_Os03g59180 | 1.041 | *n.s.* | *n.s.* | 1.0393 | *n.s.* | -0.9395 | 1.0109 |
| LOC_Os03g59320 | *n.s.* | *n.s.* | *n.s.* | *n.s.* | -1.1206 | *n.s.* | *n.s.* |
| LOC_Os03g59360 | *n.s.* | *n.s.* | *n.s.* | *n.s.* | *n.s.* | -0.624 | -1.2504 |
| LOC_Os03g59430 | *n.s.* | *n.s.* | 1.1866 | *n.s.* | *n.s.* | *n.s.* | *n.s.* |
| LOC_Os03g60080 | 1.4507 | *n.s.* | -0.9308 | *n.s.* | *n.s.* | -1.6383 | *n.s.* |
| LOC_Os03g60260 | *n.s.* | *n.s.* | *n.s.* | *n.s.* | *n.s.* | *n.s.* | -1.5632 |
| LOC_Os03g60370 | *n.s.* | *n.s.* | -0.6877 | *n.s.* | -0.9658 | -1.0504 | -1.0577 |
| LOC_Os03g60560 | 1.7408 | *n.s.* | *n.s.* | *n.s.* | *n.s.* | -1.5908 | -2.1123 |
| LOC_Os03g60570 | 5.7132 | *n.s.* | *n.s.* | *n.s.* | *n.s.* | -1.9774 | -2.2691 |
| LOC_Os03g60580 | *n.s.* | *n.s.* | *n.s.* | -2.5948 | *n.s.* | -1.9932 | -3.3147 |
| LOC_Os03g61360 | *n.s.* | *n.s.* | -2.4518 | *n.s.* | *n.s.* | -2.2968 | *n.s.* |
| LOC_Os03g61920 | *n.s.* | *n.s.* | *n.s.* | *n.s.* | *n.s.* | -1.1524 | -1.104 |
| LOC_Os03g62580 | *n.s.* | *n.s.* | *n.s.* | *n.s.* | *n.s.* | 0.7908 | *n.s.* |
| LOC_Os04g12499 | -2.2152 | *n.s.* | -0.7563 | *n.s.* | -2.391 | *n.s.* | *n.s.* |
| LOC_Os04g17100 | *n.s.* | 1.2937 | *n.s.* | *n.s.* | -0.9255 | -1.5263 | *n.s.* |
| LOC_Os04g19740 | *n.s.* | *n.s.* | *n.s.* | *n.s.* | *n.s.* | *n.s.* | -1.4795 |
| LOC_Os04g23580 | *n.s.* | *n.s.* | *n.s.* | *n.s.* | -0.8957 | -1.3056 | *n.s.* |
| LOC_Os04g31340 | *n.s.* | *n.s.* | *n.s.* | *n.s.* | *n.s.* | 1.0654 | *n.s.* |
| LOC_Os04g32620 | *n.s.* | *n.s.* | *n.s.* | *n.s.* | *n.s.* | -0.9064 | *n.s.* |
| LOC_Os04g33150 | *n.s.* | 5.7571 | *n.s.* | *n.s.* | *n.s.* | *n.s.* | *n.s.* |
| LOC_Os04g33240 | *n.s.* | *n.s.* | *n.s.* | *n.s.* | *n.s.* | *n.s.* | -1.474 |
| LOC_Os04g33490 | 0.7251 | *n.s.* | *n.s.* | *n.s.* | *n.s.* | -0.8219 | *n.s.* |
| LOC_Os04g33640 | 1.6045 | *n.s.* | *n.s.* | *n.s.* | *n.s.* | *n.s.* | *n.s.* |
| LOC_Os04g34250 | *n.s.* | *n.s.* | 1.0542 | *n.s.* | *n.s.* | 1.8274 | 1.3366 |
| LOC_Os04g35100 | *n.s.* | 1.8987 | *n.s.* | *n.s.* | *n.s.* | *n.s.* | *n.s.* |
| LOC_Os04g35130 | 1.1331 | *n.s.* | *n.s.* | *n.s.* | *n.s.* | -1.542 | -1.0779 |
| LOC_Os04g35270 | *n.s.* | *n.s.* | *n.s.* | *n.s.* | *n.s.* | -3.6127 | *n.s.* |
| LOC_Os04g35280 | *n.s.* | *n.s.* | 0.6554 | *n.s.* | *n.s.* | *n.s.* | *n.s.* |
| LOC_Os04g35840 | *n.s.* | *n.s.* | 0.7353 | 1.1302 | *n.s.* | 0.9871 | 1.0939 |
| LOC_Os04g36040 | *n.s.* | *n.s.* | *n.s.* | *n.s.* | -0.9229 | *n.s.* | *n.s.* |
| LOC_Os04g37490 | *n.s.* | *n.s.* | -1.118 | *n.s.* | -1.5722 | -1.6561 | *n.s.* |
| LOC_Os04g37500 | -1.6775 | *n.s.* | 1.8228 | *n.s.* | *n.s.* | *n.s.* | *n.s.* |
| LOC_Os04g37550 | *n.s.* | *n.s.* | *n.s.* | *n.s.* | *n.s.* | *n.s.* | -0.9077 |
| LOC_Os04g38720 | *n.s.* | *n.s.* | *n.s.* | *n.s.* | *n.s.* | *n.s.* | -1.0245 |
| LOC_Os04g39010 | *n.s.* | *n.s.* | *n.s.* | *n.s.* | *n.s.* | *n.s.* | 2.7045 |
| LOC_Os04g39489 | *n.s.* | *n.s.* | 1.0197 | *n.s.* | *n.s.* | 1.3351 | *n.s.* |
| LOC_Os04g39540 | *n.s.* | *n.s.* | -0.7613 | *n.s.* | *n.s.* | *n.s.* | *n.s.* |
| LOC_Os04g41460 | *n.s.* | *n.s.* | *n.s.* | *n.s.* | *n.s.* | 0.5206 | *n.s.* |
| LOC_Os04g41570 | 0.9607 | *n.s.* | *n.s.* | *n.s.* | -1.0969 | -1.6414 | -1.6743 |
| LOC_Os04g42470 | *n.s.* | *n.s.* | *n.s.* | *n.s.* | *n.s.* | 1.1028 | *n.s.* |
| LOC_Os04g42700 | -1.0644 | *n.s.* | *n.s.* | -1.2745 | *n.s.* | -0.6845 | -1.4044 |
| LOC_Os04g43200 | *n.s.* | *n.s.* | *n.s.* | *n.s.* | *n.s.* | -2.3641 | -4.1137 |
| LOC_Os04g43990 | *n.s.* | *n.s.* | 1.6534 | *n.s.* | 2.2338 | 1.5585 | 0.916 |
| LOC_Os04g44530 | 0.8993 | 1.3544 | *n.s.* | *n.s.* | -1.3728 | -1.405 | *n.s.* |
| LOC_Os04g45810 | *n.s.* | 1.0734 | *n.s.* | *n.s.* | -0.7375 | -1.3306 | *n.s.* |
| LOC_Os04g46440 | 2.0547 | *n.s.* | *n.s.* | *n.s.* | *n.s.* | *n.s.* | *n.s.* |
| LOC_Os04g47140 | *n.s.* | *n.s.* | *n.s.* | 1.3395 | *n.s.* | *n.s.* | *n.s.* |
| LOC_Os04g47720 | *n.s.* | *n.s.* | *n.s.* | *n.s.* | *n.s.* | -1.6664 | *n.s.* |
| LOC_Os04g48070 | *n.s.* | *n.s.* | *n.s.* | *n.s.* | -0.8895 | *n.s.* | *n.s.* |
| LOC_Os04g48270 | *n.s.* | *n.s.* | *n.s.* | *n.s.* | *n.s.* | *n.s.* | -1.1252 |
| LOC_Os04g48390 | *n.s.* | *n.s.* | *n.s.* | *n.s.* | *n.s.* | 0.5685 | *n.s.* |
| LOC_Os04g48830 | *n.s.* | *n.s.* | *n.s.* | *n.s.* | 1.5648 | *n.s.* | *n.s.* |
| LOC_Os04g49980 | *n.s.* | *n.s.* | *n.s.* | *n.s.* | *n.s.* | *n.s.* | -4.4965 |
| LOC_Os04g51160 | *n.s.* | *n.s.* | *n.s.* | *n.s.* | *n.s.* | -1.9618 | *n.s.* |
| LOC_Os04g51460 | 4.9642 | *n.s.* | *n.s.* | *n.s.* | -2.5661 | -3.3708 | *n.s.* |
| LOC_Os04g51610 | *n.s.* | *n.s.* | *n.s.* | -0.9805 | -0.932 | -1.0631 | -0.6483 |
| LOC_Os04g51970 | *n.s.* | *n.s.* | *n.s.* | *n.s.* | -0.8889 | *n.s.* | *n.s.* |
| LOC_Os04g52390 | *n.s.* | *n.s.* | -0.8603 | *n.s.* | -0.6382 | -0.5935 | *n.s.* |
| LOC_Os04g53970 | *n.s.* | *n.s.* | *n.s.* | 1.9612 | *n.s.* | *n.s.* | *n.s.* |
| LOC_Os04g54010 | *n.s.* | *n.s.* | *n.s.* | *n.s.* | *n.s.* | *n.s.* | 1.1747 |
| LOC_Os04g54300 | *n.s.* | 1.6151 | *n.s.* | *n.s.* | *n.s.* | *n.s.* | *n.s.* |
| LOC_Os04g55700 | *n.s.* | *n.s.* | *n.s.* | *n.s.* | *n.s.* | -0.6972 | *n.s.* |
| LOC_Os04g56580 | *n.s.* | *n.s.* | *n.s.* | *n.s.* | *n.s.* | *n.s.* | -0.7602 |
| LOC_Os04g57440 | *n.s.* | *n.s.* | *n.s.* | *n.s.* | *n.s.* | -0.8195 | -1.1564 |
| LOC_Os04g57550 | 1.3471 | *n.s.* | -0.5676 | *n.s.* | -0.7783 | -1.2762 | *n.s.* |
| LOC_Os04g57739 | 1.3646 | *n.s.* | *n.s.* | *n.s.* | *n.s.* | -1.3792 | *n.s.* |
| LOC_Os04g57760 | 0.9452 | *n.s.* | *n.s.* | *n.s.* | *n.s.* | -1.0612 | *n.s.* |
| LOC_Os04g57810 | 1.4254 | *n.s.* | -0.6566 | *n.s.* | -1.3105 | -1.4766 | -0.8922 |
| LOC_Os04g58320 | *n.s.* | *n.s.* | *n.s.* | *n.s.* | *n.s.* | *n.s.* | -0.6889 |
| LOC_Os04g58890 | 1.5528 | *n.s.* | *n.s.* | 1.0256 | *n.s.* | *n.s.* | *n.s.* |
| LOC_Os04g59420 | 1.602 | *n.s.* | *n.s.* | *n.s.* | *n.s.* | *n.s.* | *n.s.* |
| LOC_Os04g59540 | *n.s.* | 2.4575 | *n.s.* | *n.s.* | *n.s.* | 2.2247 | *n.s.* |
| LOC_Os04g59580 | *n.s.* | *n.s.* | *n.s.* | *n.s.* | *n.s.* | 0.8639 | *n.s.* |
| LOC_Os05g01240 | *n.s.* | *n.s.* | *n.s.* | *n.s.* | *n.s.* | *n.s.* | -1.3127 |
| LOC_Os05g01760 | *n.s.* | *n.s.* | -0.4943 | *n.s.* | *n.s.* | *n.s.* | *n.s.* |
| LOC_Os05g02770 | *n.s.* | *n.s.* | *n.s.* | *n.s.* | *n.s.* | -0.7438 | *n.s.* |
| LOC_Os05g03070 | *n.s.* | *n.s.* | 2.1646 | *n.s.* | *n.s.* | 1.9603 | *n.s.* |
| LOC_Os05g03460 | *n.s.* | *n.s.* | *n.s.* | *n.s.* | *n.s.* | -0.891 | -1.2146 |
| LOC_Os05g03920 | 0.7847 | *n.s.* | *n.s.* | *n.s.* | *n.s.* | *n.s.* | *n.s.* |
| LOC_Os05g04360 | *n.s.* | *n.s.* | *n.s.* | *n.s.* | *n.s.* | -0.6295 | *n.s.* |
| LOC_Os05g04700 | 1.168 | *n.s.* | -0.6538 | *n.s.* | -1.5855 | -1.2167 | -1.2441 |
| LOC_Os05g06990 | 1.4362 | *n.s.* | *n.s.* | *n.s.* | *n.s.* | -1.4885 | *n.s.* |
| LOC_Os05g09380 | *n.s.* | *n.s.* | *n.s.* | *n.s.* | *n.s.* | -0.7882 | *n.s.* |
| LOC_Os05g10650 | *n.s.* | *n.s.* | *n.s.* | *n.s.* | *n.s.* | -3.6306 | *n.s.* |
| LOC_Os05g10690 | *n.s.* | 1.8483 | *n.s.* | *n.s.* | *n.s.* | *n.s.* | *n.s.* |
| LOC_Os05g10930 | 0.864 | *n.s.* | *n.s.* | 1.0107 | *n.s.* | -0.7159 | *n.s.* |
| LOC_Os05g11620 | *n.s.* | *n.s.* | *n.s.* | *n.s.* | *n.s.* | -2.2592 | -2.2003 |
| LOC_Os05g11910 | *n.s.* | *n.s.* | *n.s.* | *n.s.* | *n.s.* | 1.4147 | *n.s.* |
| LOC_Os05g21180 | 1.1183 | *n.s.* | -0.7013 | *n.s.* | -1.1296 | -1.1527 | *n.s.* |
| LOC_Os05g25260 | 0.7729 | *n.s.* | *n.s.* | *n.s.* | *n.s.* | -0.6948 | *n.s.* |
| LOC_Os05g26940 | *n.s.* | *n.s.* | *n.s.* | 1.3659 | *n.s.* | 1.6392 | 1.7569 |
| LOC_Os05g27780 | *n.s.* | *n.s.* | *n.s.* | -1.1584 | 1.296 | *n.s.* | -1.3288 |
| LOC_Os05g28740 | *n.s.* | 1.8711 | *n.s.* | *n.s.* | 1.9629 | *n.s.* | *n.s.* |
| LOC_Os05g31020 | *n.s.* | *n.s.* | *n.s.* | *n.s.* | *n.s.* | *n.s.* | -2.1785 |
| LOC_Os05g31056 | *n.s.* | *n.s.* | *n.s.* | *n.s.* | *n.s.* | *n.s.* | 1.2244 |
| LOC_Os05g31620 | *n.s.* | *n.s.* | *n.s.* | *n.s.* | *n.s.* | -1.2538 | *n.s.* |
| LOC_Os05g31670 | *n.s.* | *n.s.* | *n.s.* | *n.s.* | *n.s.* | -3.206 | -3.5773 |
| LOC_Os05g32320 | *n.s.* | *n.s.* | *n.s.* | *n.s.* | -0.9389 | -0.7881 | -0.7058 |
| LOC_Os05g32630 | *n.s.* | *n.s.* | *n.s.* | *n.s.* | *n.s.* | -0.5502 | *n.s.* |
| LOC_Os05g33010 | *n.s.* | *n.s.* | *n.s.* | *n.s.* | *n.s.* | 0.5808 | *n.s.* |
| LOC_Os05g33900 | *n.s.* | *n.s.* | *n.s.* | *n.s.* | -0.9219 | *n.s.* | *n.s.* |
| LOC_Os05g33960 | *n.s.* | *n.s.* | *n.s.* | *n.s.* | *n.s.* | -1.9141 | -3.0117 |
| LOC_Os05g34830 | *n.s.* | *n.s.* | -0.6758 | *n.s.* | *n.s.* | -1.1902 | *n.s.* |
| LOC_Os05g37450 | 0.9411 | *n.s.* | *n.s.* | *n.s.* | *n.s.* | *n.s.* | *n.s.* |
| LOC_Os05g38150 | *n.s.* | *n.s.* | *n.s.* | -0.9214 | *n.s.* | *n.s.* | -0.7461 |
| LOC_Os05g38270 | *n.s.* | *n.s.* | *n.s.* | *n.s.* | -2.4306 | -1.4942 | *n.s.* |
| LOC_Os05g38290 | *n.s.* | *n.s.* | *n.s.* | *n.s.* | -1.9794 | -2.1383 | -1.4435 |
| LOC_Os05g38350 | *n.s.* | *n.s.* | *n.s.* | *n.s.* | *n.s.* | -0.971 | *n.s.* |
| LOC_Os05g38710 | *n.s.* | *n.s.* | *n.s.* | *n.s.* | -0.8739 | *n.s.* | *n.s.* |
| LOC_Os05g39060 | 0.6683 | *n.s.* | *n.s.* | *n.s.* | -1.3396 | -0.637 | *n.s.* |
| LOC_Os05g39250 | *n.s.* | *n.s.* | *n.s.* | *n.s.* | *n.s.* | -1.9321 | -2.3895 |
| LOC_Os05g39410 | 0.7883 | *n.s.* | -0.969 | *n.s.* | -1.0529 | -1.2723 | *n.s.* |
| LOC_Os05g39760 | *n.s.* | *n.s.* | *n.s.* | *n.s.* | *n.s.* | *n.s.* | 0.7681 |
| LOC_Os05g39930 | 1.6467 | *n.s.* | *n.s.* | *n.s.* | *n.s.* | -1.7886 | *n.s.* |
| LOC_Os05g41200 | *n.s.* | *n.s.* | *n.s.* | *n.s.* | *n.s.* | 1.1843 | *n.s.* |
| LOC_Os05g41490 | *n.s.* | 2.4269 | *n.s.* | *n.s.* | *n.s.* | -3.3008 | *n.s.* |
| LOC_Os05g42040 | 0.6655 | *n.s.* | *n.s.* | *n.s.* | *n.s.* | -0.8328 | -1.0886 |
| LOC_Os05g43390 | *n.s.* | *n.s.* | -1.408 | *n.s.* | *n.s.* | -1.807 | -2.373 |
| LOC_Os05g44570 | *n.s.* | *n.s.* | *n.s.* | *n.s.* | *n.s.* | 0.9403 | *n.s.* |
| LOC_Os05g45020 | *n.s.* | *n.s.* | *n.s.* | *n.s.* | *n.s.* | *n.s.* | -1.8584 |
| LOC_Os05g45320 | *n.s.* | *n.s.* | *n.s.* | *n.s.* | *n.s.* | -0.809 | *n.s.* |
| LOC_Os05g45810 | *n.s.* | *n.s.* | *n.s.* | *n.s.* | -1.4414 | *n.s.* | *n.s.* |
| LOC_Os05g46040 | *n.s.* | *n.s.* | *n.s.* | *n.s.* | -1.0699 | -0.5081 | *n.s.* |
| LOC_Os05g46460 | *n.s.* | *n.s.* | *n.s.* | *n.s.* | -0.6996 | *n.s.* | *n.s.* |
| LOC_Os05g46510 | *n.s.* | *n.s.* | *n.s.* | *n.s.* | *n.s.* | *n.s.* | -1.5354 |
| LOC_Os05g46760 | *n.s.* | *n.s.* | *n.s.* | *n.s.* | *n.s.* | -2.265 | -1.3443 |
| LOC_Os05g47660 | *n.s.* | *n.s.* | *n.s.* | *n.s.* | *n.s.* | -1.4128 | *n.s.* |
| LOC_Os05g48060 | *n.s.* | *n.s.* | *n.s.* | 1.1176 | *n.s.* | *n.s.* | *n.s.* |
| LOC_Os05g48330 | *n.s.* | *n.s.* | *n.s.* | *n.s.* | *n.s.* | -1.5101 | *n.s.* |
| LOC_Os05g48340 | *n.s.* | *n.s.* | *n.s.* | *n.s.* | *n.s.* | *n.s.* | -0.7595 |
| LOC_Os05g48650 | *n.s.* | *n.s.* | *n.s.* | -1.7498 | *n.s.* | *n.s.* | *n.s.* |
| LOC_Os05g49170 | *n.s.* | *n.s.* | *n.s.* | -1.0284 | *n.s.* | *n.s.* | -1.3994 |
| LOC_Os05g49300 | -0.7452 | *n.s.* | *n.s.* | *n.s.* | *n.s.* | *n.s.* | *n.s.* |
| LOC_Os05g49350 | *n.s.* | *n.s.* | *n.s.* | *n.s.* | -1.2393 | *n.s.* | *n.s.* |
| LOC_Os05g49730 | *n.s.* | 4.0609 | *n.s.* | *n.s.* | *n.s.* | *n.s.* | -4.3914 |
| LOC_Os05g49770 | 0.9161 | *n.s.* | -0.7752 | *n.s.* | *n.s.* | -1.174 | *n.s.* |
| LOC_Os05g49940 | *n.s.* | *n.s.* | *n.s.* | *n.s.* | *n.s.* | -2.6045 | -2.781 |
| LOC_Os05g50380 | *n.s.* | *n.s.* | *n.s.* | -1.6559 | 1.455 | -2.4523 | -1.6793 |
| LOC_Os05g51630 | -0.5943 | *n.s.* | *n.s.* | *n.s.* | -0.7968 | -0.5845 | *n.s.* |
| LOC_Os05g51670 | 0.915 | *n.s.* | *n.s.* | *n.s.* | *n.s.* | -0.8616 | *n.s.* |
| LOC_Os06g01360 | *n.s.* | *n.s.* | *n.s.* | *n.s.* | *n.s.* | -0.57 | *n.s.* |
| LOC_Os06g01630 | *n.s.* | *n.s.* | *n.s.* | *n.s.* | *n.s.* | -0.8659 | *n.s.* |
| LOC_Os06g03540 | *n.s.* | *n.s.* | *n.s.* | *n.s.* | *n.s.* | *n.s.* | 2.5792 |
| LOC_Os06g03800 | *n.s.* | *n.s.* | 0.8853 | *n.s.* | *n.s.* | 1.6434 | 1.471 |
| LOC_Os06g04220 | 4.0218 | *n.s.* | *n.s.* | *n.s.* | *n.s.* | -3.5705 | *n.s.* |
| LOC_Os06g04230 | 2.1018 | *n.s.* | *n.s.* | *n.s.* | *n.s.* | -2.1053 | *n.s.* |
| LOC_Os06g04240 | 2.8361 | *n.s.* | *n.s.* | *n.s.* | *n.s.* | *n.s.* | -1.3053 |
| LOC_Os06g04480 | *n.s.* | *n.s.* | *n.s.* | *n.s.* | *n.s.* | *n.s.* | -1.3319 |
| LOC_Os06g05320 | *n.s.* | *n.s.* | *n.s.* | *n.s.* | *n.s.* | -1.5827 | *n.s.* |
| LOC_Os06g05420 | *n.s.* | *n.s.* | *n.s.* | *n.s.* | -1.0622 | *n.s.* | -3.2075 |
| LOC_Os06g05470 | *n.s.* | *n.s.* | *n.s.* | *n.s.* | *n.s.* | -1.7551 | *n.s.* |
| LOC_Os06g06080 | -1.4824 | *n.s.* | *n.s.* | *n.s.* | *n.s.* | 0.6141 | -0.7312 |
| LOC_Os06g06760 | *n.s.* | *n.s.* | *n.s.* | *n.s.* | *n.s.* | 1.9187 | 1.4659 |
| LOC_Os06g07030 | *n.s.* | *n.s.* | *n.s.* | *n.s.* | *n.s.* | *n.s.* | -1.4147 |
| LOC_Os06g08110 | *n.s.* | *n.s.* | *n.s.* | *n.s.* | *n.s.* | *n.s.* | -1.3657 |
| LOC_Os06g08280 | *n.s.* | *n.s.* | *n.s.* | *n.s.* | *n.s.* | -0.9487 | *n.s.* |
| LOC_Os06g10880 | *n.s.* | 1.5666 | *n.s.* | *n.s.* | *n.s.* | *n.s.* | *n.s.* |
| LOC_Os06g11090 | *n.s.* | *n.s.* | *n.s.* | *n.s.* | *n.s.* | -1.0066 | *n.s.* |
| LOC_Os06g11450 | *n.s.* | *n.s.* | *n.s.* | *n.s.* | *n.s.* | -1.1371 | *n.s.* |
| LOC_Os06g11660 | 1.7228 | *n.s.* | *n.s.* | *n.s.* | *n.s.* | *n.s.* | *n.s.* |
| LOC_Os06g11980 | *n.s.* | *n.s.* | *n.s.* | *n.s.* | *n.s.* | -2.7157 | *n.s.* |
| LOC_Os06g12370 | *n.s.* | *n.s.* | *n.s.* | *n.s.* | *n.s.* | *n.s.* | -2.6148 |
| LOC_Os06g13720 | *n.s.* | *n.s.* | *n.s.* | *n.s.* | *n.s.* | -1.2976 | *n.s.* |
| LOC_Os06g16370 | *n.s.* | *n.s.* | *n.s.* | *n.s.* | *n.s.* | *n.s.* | -1.0606 |
| LOC_Os06g19630 | *n.s.* | *n.s.* | *n.s.* | *n.s.* | *n.s.* | -2.4749 | *n.s.* |
| LOC_Os06g20040 | *n.s.* | *n.s.* | 2.366 | *n.s.* | *n.s.* | 1.6979 | *n.s.* |
| LOC_Os06g21380 | *n.s.* | *n.s.* | *n.s.* | 1.1908 | *n.s.* | *n.s.* | *n.s.* |
| LOC_Os06g23684 | *n.s.* | *n.s.* | 1.3008 | *n.s.* | *n.s.* | 2.401 | *n.s.* |
| LOC_Os06g24730 | 1.0282 | *n.s.* | *n.s.* | *n.s.* | *n.s.* | -0.7833 | *n.s.* |
| LOC_Os06g24990 | *n.s.* | *n.s.* | *n.s.* | -1.6542 | *n.s.* | -1.0931 | -2.3062 |
| LOC_Os06g27560 | *n.s.* | *n.s.* | *n.s.* | *n.s.* | *n.s.* | -0.7964 | *n.s.* |
| LOC_Os06g30130 | *n.s.* | *n.s.* | *n.s.* | *n.s.* | *n.s.* | -0.6221 | *n.s.* |
| LOC_Os06g30370 | *n.s.* | 2.2075 | *n.s.* | *n.s.* | *n.s.* | *n.s.* | *n.s.* |
| LOC_Os06g33330 | -1.5848 | *n.s.* | *n.s.* | *n.s.* | *n.s.* | *n.s.* | *n.s.* |
| LOC_Os06g34040 | *n.s.* | *n.s.* | 0.7269 | *n.s.* | 0.6821 | 0.9751 | *n.s.* |
| LOC_Os06g35960 | *n.s.* | *n.s.* | *n.s.* | *n.s.* | -1.915 | -1.0942 | -2.6347 |
| LOC_Os06g36390 | *n.s.* | *n.s.* | *n.s.* | *n.s.* | 1.5443 | 1.2443 | *n.s.* |
| LOC_Os06g39370 | *n.s.* | *n.s.* | *n.s.* | *n.s.* | 0.8711 | *n.s.* | *n.s.* |
| LOC_Os06g40060 | *n.s.* | *n.s.* | *n.s.* | *n.s.* | *n.s.* | -0.7215 | *n.s.* |
| LOC_Os06g41360 | *n.s.* | *n.s.* | *n.s.* | *n.s.* | -0.7722 | *n.s.* | -1.0357 |
| LOC_Os06g42030 | *n.s.* | *n.s.* | *n.s.* | -1.1715 | *n.s.* | -1.228 | -1.5574 |
| LOC_Os06g42850 | *n.s.* | 1.373 | *n.s.* | *n.s.* | *n.s.* | -1.1744 | 0.7256 |
| LOC_Os06g43090 | 1.6228 | *n.s.* | *n.s.* | *n.s.* | *n.s.* | -2.2632 | -1.7634 |
| LOC_Os06g44040 | 1.337 | *n.s.* | *n.s.* | *n.s.* | *n.s.* | -1.1749 | *n.s.* |
| LOC_Os06g44160 | *n.s.* | *n.s.* | *n.s.* | *n.s.* | *n.s.* | -1.9345 | -1.2512 |
| LOC_Os06g44250 | *n.s.* | *n.s.* | *n.s.* | *n.s.* | *n.s.* | -1.4488 | *n.s.* |
| LOC_Os06g46740 | *n.s.* | *n.s.* | *n.s.* | -3.42 | *n.s.* | -2.1859 | -5.137 |
| LOC_Os06g46900 | *n.s.* | *n.s.* | 1.9506 | *n.s.* | *n.s.* | *n.s.* | *n.s.* |
| LOC_Os06g46940 | *n.s.* | *n.s.* | 2.7808 | *n.s.* | *n.s.* | *n.s.* | *n.s.* |
| LOC_Os06g46950 | 2.5666 | *n.s.* | *n.s.* | *n.s.* | *n.s.* | -2.1962 | *n.s.* |
| LOC_Os06g47200 | *n.s.* | *n.s.* | *n.s.* | *n.s.* | *n.s.* | *n.s.* | -1.4579 |
| LOC_Os06g48160 | 4.5637 | *n.s.* | *n.s.* | *n.s.* | *n.s.* | *n.s.* | *n.s.* |
| LOC_Os06g48180 | *n.s.* | *n.s.* | *n.s.* | *n.s.* | -1.0511 | *n.s.* | *n.s.* |
| LOC_Os06g48200 | 1.6254 | *n.s.* | *n.s.* | *n.s.* | *n.s.* | *n.s.* | *n.s.* |
| LOC_Os06g48300 | *n.s.* | *n.s.* | *n.s.* | *n.s.* | *n.s.* | -0.7517 | *n.s.* |
| LOC_Os06g48500 | -2.0335 | *n.s.* | *n.s.* | *n.s.* | *n.s.* | *n.s.* | -1.1305 |
| LOC_Os06g48810 | *n.s.* | 3.0253 | 1.871 | *n.s.* | *n.s.* | 1.9127 | *n.s.* |
| LOC_Os06g49640 | *n.s.* | *n.s.* | *n.s.* | *n.s.* | *n.s.* | -0.7906 | *n.s.* |
| LOC_Os06g50920 | *n.s.* | -1.6129 | *n.s.* | *n.s.* | *n.s.* | 1.1922 | *n.s.* |
| LOC_Os06g51260 | -1.6577 | *n.s.* | *n.s.* | *n.s.* | *n.s.* | -0.7631 | *n.s.* |
| LOC_Os07g03120 | *n.s.* | *n.s.* | *n.s.* | *n.s.* | -1.3715 | -2.0724 | *n.s.* |
| LOC_Os07g04040 | *n.s.* | *n.s.* | *n.s.* | *n.s.* | *n.s.* | -1.1612 | *n.s.* |
| LOC_Os07g05940 | 7.1078 | 3.2167 | *n.s.* | *n.s.* | *n.s.* | *n.s.* | -6.6624 |
| LOC_Os07g07040 | *n.s.* | *n.s.* | -0.7384 | *n.s.* | -1.087 | -1.4648 | -1.8227 |
| LOC_Os07g07410 | 0.8281 | *n.s.* | *n.s.* | *n.s.* | *n.s.* | *n.s.* | -1.4642 |
| LOC_Os07g07930 | *n.s.* | *n.s.* | *n.s.* | *n.s.* | *n.s.* | -2.0935 | -2.0147 |
| LOC_Os07g07974 | *n.s.* | *n.s.* | -0.6252 | *n.s.* | *n.s.* | *n.s.* | -0.7151 |
| LOC_Os07g08140 | *n.s.* | *n.s.* | *n.s.* | *n.s.* | *n.s.* | -0.9254 | *n.s.* |
| LOC_Os07g08460 | *n.s.* | *n.s.* | *n.s.* | *n.s.* | *n.s.* | 1.4808 | *n.s.* |
| LOC_Os07g08970 | *n.s.* | *n.s.* | -0.6158 | 1.114 | -0.7808 | -0.6756 | 0.9551 |
| LOC_Os07g09670 | 1.2328 | *n.s.* | *n.s.* | *n.s.* | -1.2094 | -1.0399 | *n.s.* |
| LOC_Os07g10840 | -1.9324 | *n.s.* | 0.7395 | *n.s.* | *n.s.* | *n.s.* | *n.s.* |
| LOC_Os07g16970 | *n.s.* | *n.s.* | *n.s.* | *n.s.* | *n.s.* | -1.0257 | *n.s.* |
| LOC_Os07g19444 | *n.s.* | *n.s.* | *n.s.* | *n.s.* | *n.s.* | -0.8513 | *n.s.* |
| LOC_Os07g23570 | *n.s.* | *n.s.* | *n.s.* | *n.s.* | *n.s.* | *n.s.* | -4.6353 |
| LOC_Os07g23640 | *n.s.* | *n.s.* | *n.s.* | *n.s.* | *n.s.* | 1.7212 | *n.s.* |
| LOC_Os07g23660 | *n.s.* | *n.s.* | *n.s.* | *n.s.* | *n.s.* | 2.4549 | *n.s.* |
| LOC_Os07g35510 | -2.9124 | *n.s.* | *n.s.* | *n.s.* | *n.s.* | *n.s.* | -1.4822 |
| LOC_Os07g36170 | 0.7115 | *n.s.* | *n.s.* | *n.s.* | *n.s.* | *n.s.* | *n.s.* |
| LOC_Os07g36400 | *n.s.* | *n.s.* | *n.s.* | 1.4147 | *n.s.* | *n.s.* | *n.s.* |
| LOC_Os07g37210 | *n.s.* | *n.s.* | *n.s.* | *n.s.* | *n.s.* | *n.s.* | -2.325 |
| LOC_Os07g37620 | 1.8205 | *n.s.* | -0.7498 | *n.s.* | -1.4021 | *n.s.* | *n.s.* |
| LOC_Os07g39270 | *n.s.* | *n.s.* | *n.s.* | *n.s.* | *n.s.* | 1.3497 | *n.s.* |
| LOC_Os07g39300 | *n.s.* | *n.s.* | *n.s.* | *n.s.* | *n.s.* | 0.8272 | *n.s.* |
| LOC_Os07g39520 | *n.s.* | *n.s.* | *n.s.* | *n.s.* | -0.9572 | -1.3789 | *n.s.* |
| LOC_Os07g39530 | *n.s.* | *n.s.* | *n.s.* | *n.s.* | *n.s.* | *n.s.* | -1.4162 |
| LOC_Os07g39740 | *n.s.* | *n.s.* | *n.s.* | *n.s.* | *n.s.* | -1.4019 | *n.s.* |
| LOC_Os07g40300 | *n.s.* | *n.s.* | *n.s.* | *n.s.* | *n.s.* | 3.3112 | *n.s.* |
| LOC_Os07g41140 | *n.s.* | *n.s.* | *n.s.* | *n.s.* | *n.s.* | -0.5989 | *n.s.* |
| LOC_Os07g42160 | 1.3786 | *n.s.* | *n.s.* | *n.s.* | *n.s.* | -1.0054 | -1.6193 |
| LOC_Os07g42220 | *n.s.* | *n.s.* | 0.7657 | *n.s.* | *n.s.* | *n.s.* | *n.s.* |
| LOC_Os07g42280 | *n.s.* | *n.s.* | *n.s.* | *n.s.* | -1.6639 | *n.s.* | *n.s.* |
| LOC_Os07g42700 | *n.s.* | *n.s.* | *n.s.* | *n.s.* | *n.s.* | *n.s.* | -0.7246 |
| LOC_Os07g43950 | 0.8937 | *n.s.* | *n.s.* | *n.s.* | *n.s.* | *n.s.* | *n.s.* |
| LOC_Os07g44140 | *n.s.* | *n.s.* | -2.193 | *n.s.* | *n.s.* | -2.348 | *n.s.* |
| LOC_Os07g44330 | *n.s.* | *n.s.* | *n.s.* | 1.0004 | *n.s.* | *n.s.* | 1.4757 |
| LOC_Os07g44410 | -1.3717 | *n.s.* | *n.s.* | *n.s.* | *n.s.* | -0.8404 | -2.5495 |
| LOC_Os07g44850 | *n.s.* | *n.s.* | *n.s.* | *n.s.* | *n.s.* | *n.s.* | -4.5726 |
| LOC_Os07g45000 | *n.s.* | *n.s.* | *n.s.* | *n.s.* | -0.7078 | *n.s.* | *n.s.* |
| LOC_Os07g46220 | *n.s.* | *n.s.* | *n.s.* | *n.s.* | -0.8397 | -0.8445 | -0.8721 |
| LOC_Os07g46630 | *n.s.* | *n.s.* | *n.s.* | *n.s.* | *n.s.* | -0.9121 | -1.1767 |
| LOC_Os07g47590 | *n.s.* | *n.s.* | *n.s.* | *n.s.* | *n.s.* | 0.8215 | -0.7527 |
| LOC_Os07g47670 | *n.s.* | *n.s.* | -1.4306 | *n.s.* | *n.s.* | -2.1464 | *n.s.* |
| LOC_Os07g48090 | 1.2661 | *n.s.* | *n.s.* | *n.s.* | *n.s.* | -0.7056 | -0.8349 |
| LOC_Os07g48100 | *n.s.* | *n.s.* | *n.s.* | *n.s.* | *n.s.* | -1.2993 | *n.s.* |
| LOC_Os07g48229 | *n.s.* | *n.s.* | *n.s.* | *n.s.* | *n.s.* | *n.s.* | 0.8912 |
| LOC_Os07g48450 | *n.s.* | *n.s.* | *n.s.* | *n.s.* | *n.s.* | -2.1727 | *n.s.* |
| LOC_Os07g48550 | *n.s.* | *n.s.* | 0.6734 | *n.s.* | *n.s.* | -1.2877 | *n.s.* |
| LOC_Os07g48830 | 2.0687 | *n.s.* | *n.s.* | *n.s.* | *n.s.* | -1.1405 | *n.s.* |
| LOC_Os07g49270 | *n.s.* | *n.s.* | *n.s.* | *n.s.* | *n.s.* | -0.8394 | *n.s.* |
| LOC_Os07g49460 | *n.s.* | *n.s.* | -1.6593 | *n.s.* | *n.s.* | -2.1959 | *n.s.* |
| LOC_Os07g49470 | *n.s.* | *n.s.* | *n.s.* | *n.s.* | *n.s.* | -0.7482 | *n.s.* |
| LOC_Os08g01410 | *n.s.* | *n.s.* | *n.s.* | *n.s.* | -1.0081 | -0.7272 | *n.s.* |
| LOC_Os08g01490 | -0.9994 | *n.s.* | *n.s.* | *n.s.* | *n.s.* | *n.s.* | *n.s.* |
| LOC_Os08g02030 | *n.s.* | *n.s.* | *n.s.* | *n.s.* | *n.s.* | -1.6688 | -2.8241 |
| LOC_Os08g04470 | *n.s.* | *n.s.* | *n.s.* | *n.s.* | *n.s.* | 0.7864 | *n.s.* |
| LOC_Os08g05780 | -3.0089 | *n.s.* | *n.s.* | -2.9768 | *n.s.* | -2.7696 | -2.7997 |
| LOC_Os08g06010 | *n.s.* | *n.s.* | -0.8874 | *n.s.* | 0.8478 | -0.682 | *n.s.* |
| LOC_Os08g07390 | *n.s.* | *n.s.* | -1.0292 | *n.s.* | -1.6271 | -0.8499 | *n.s.* |
| LOC_Os08g09240 | *n.s.* | *n.s.* | *n.s.* | *n.s.* | *n.s.* | 0.4988 | *n.s.* |
| LOC_Os08g10500 | 3.1288 | *n.s.* | *n.s.* | *n.s.* | *n.s.* | -1.8745 | -1.7648 |
| LOC_Os08g18150 | *n.s.* | *n.s.* | *n.s.* | *n.s.* | 1.4686 | *n.s.* | *n.s.* |
| LOC_Os08g18974 | *n.s.* | *n.s.* | *n.s.* | *n.s.* | *n.s.* | *n.s.* | -2.8364 |
| LOC_Os08g30020 | *n.s.* | *n.s.* | 0.8424 | *n.s.* | *n.s.* | *n.s.* | 0.808 |
| LOC_Os08g31580 | *n.s.* | *n.s.* | *n.s.* | *n.s.* | *n.s.* | -0.6682 | *n.s.* |
| LOC_Os08g31860 | 2.1775 | *n.s.* | *n.s.* | *n.s.* | -1.3988 | -3.0305 | -2.5713 |
| LOC_Os08g32980 | *n.s.* | *n.s.* | 0.8341 | *n.s.* | *n.s.* | *n.s.* | *n.s.* |
| LOC_Os08g33710 | *n.s.* | *n.s.* | *n.s.* | *n.s.* | *n.s.* | -1.4756 | -2.0011 |
| LOC_Os08g34800 | *n.s.* | *n.s.* | *n.s.* | *n.s.* | *n.s.* | -4.0529 | *n.s.* |
| LOC_Os08g35620 | *n.s.* | *n.s.* | *n.s.* | *n.s.* | *n.s.* | *n.s.* | 0.6852 |
| LOC_Os08g35860 | *n.s.* | 1.3488 | *n.s.* | 1.344 | *n.s.* | *n.s.* | *n.s.* |
| LOC_Os08g36320 | *n.s.* | *n.s.* | *n.s.* | *n.s.* | -0.6824 | *n.s.* | *n.s.* |
| LOC_Os08g36440 | *n.s.* | *n.s.* | *n.s.* | *n.s.* | *n.s.* | -1.2901 | *n.s.* |
| LOC_Os08g36860 | *n.s.* | 1.3253 | *n.s.* | *n.s.* | *n.s.* | *n.s.* | 1.3592 |
| LOC_Os08g37010 | *n.s.* | *n.s.* | *n.s.* | *n.s.* | *n.s.* | 1.2331 | *n.s.* |
| LOC_Os08g37115 | *n.s.* | *n.s.* | *n.s.* | *n.s.* | *n.s.* | 0.9593 | *n.s.* |
| LOC_Os08g37130 | *n.s.* | *n.s.* | *n.s.* | *n.s.* | -1.1404 | *n.s.* | *n.s.* |
| LOC_Os08g37840 | *n.s.* | *n.s.* | *n.s.* | *n.s.* | *n.s.* | -1.8079 | -1.7481 |
| LOC_Os08g37874 | *n.s.* | *n.s.* | *n.s.* | *n.s.* | *n.s.* | -0.9639 | *n.s.* |
| LOC_Os08g38460 | *n.s.* | *n.s.* | *n.s.* | *n.s.* | *n.s.* | -1.1018 | *n.s.* |
| LOC_Os08g38700 | *n.s.* | *n.s.* | *n.s.* | *n.s.* | 0.6884 | -0.7543 | *n.s.* |
| LOC_Os08g38880 | *n.s.* | *n.s.* | *n.s.* | *n.s.* | *n.s.* | 0.6625 | *n.s.* |
| LOC_Os08g39370 | *n.s.* | 2.0681 | *n.s.* | *n.s.* | *n.s.* | *n.s.* | *n.s.* |
| LOC_Os08g39694 | *n.s.* | *n.s.* | *n.s.* | *n.s.* | *n.s.* | 0.695 | *n.s.* |
| LOC_Os08g39730 | *n.s.* | *n.s.* | *n.s.* | *n.s.* | *n.s.* | -2.4888 | -5.9734 |
| LOC_Os08g40590 | *n.s.* | *n.s.* | *n.s.* | *n.s.* | *n.s.* | -1.1768 | *n.s.* |
| LOC_Os08g40680 | *n.s.* | *n.s.* | *n.s.* | *n.s.* | *n.s.* | -2.7473 | *n.s.* |
| LOC_Os08g41270 | *n.s.* | *n.s.* | *n.s.* | *n.s.* | -0.9061 | *n.s.* | *n.s.* |
| LOC_Os08g42420 | *n.s.* | *n.s.* | *n.s.* | *n.s.* | *n.s.* | 0.8029 | *n.s.* |
| LOC_Os08g43170 | *n.s.* | *n.s.* | *n.s.* | *n.s.* | -0.7401 | *n.s.* | *n.s.* |
| LOC_Os08g43300 | *n.s.* | *n.s.* | -0.6886 | *n.s.* | *n.s.* | *n.s.* | -0.8691 |
| LOC_Os08g44270 | 1.2745 | *n.s.* | 3.4501 | *n.s.* | *n.s.* | 2.6034 | *n.s.* |
| LOC_Os08g44340 | 1.4887 | *n.s.* | -0.8329 | *n.s.* | -1.1124 | -1.6237 | *n.s.* |
| LOC_Os08g44590 | *n.s.* | 1.8562 | 1.7653 | *n.s.* | *n.s.* | 0.8995 | 1.4319 |
| LOC_Os08g45120 | *n.s.* | *n.s.* | *n.s.* | *n.s.* | *n.s.* | -2.4016 | *n.s.* |
| LOC_Os09g01960 | 0.8567 | *n.s.* | *n.s.* | *n.s.* | *n.s.* | *n.s.* | *n.s.* |
| LOC_Os09g02770 | -1.157 | *n.s.* | *n.s.* | *n.s.* | *n.s.* | *n.s.* | *n.s.* |
| LOC_Os09g03190 | *n.s.* | *n.s.* | -1.4405 | *n.s.* | *n.s.* | -2.0118 | *n.s.* |
| LOC_Os09g15320 | *n.s.* | *n.s.* | *n.s.* | -1.6226 | *n.s.* | *n.s.* | -1.4925 |
| LOC_Os09g15670 | *n.s.* | 1.44 | *n.s.* | *n.s.* | -1.2342 | -2.2807 | *n.s.* |
| LOC_Os09g19890 | *n.s.* | *n.s.* | *n.s.* | *n.s.* | *n.s.* | -0.5476 | *n.s.* |
| LOC_Os09g20220 | *n.s.* | *n.s.* | -3.8667 | *n.s.* | *n.s.* | -3.0256 | *n.s.* |
| LOC_Os09g20930 | *n.s.* | *n.s.* | *n.s.* | 1.2839 | *n.s.* | *n.s.* | 1.7836 |
| LOC_Os09g21120 | 4.047 | 3.231 | *n.s.* | *n.s.* | *n.s.* | *n.s.* | *n.s.* |
| LOC_Os09g23150 | *n.s.* | *n.s.* | *n.s.* | 0.8641 | *n.s.* | *n.s.* | 0.8689 |
| LOC_Os09g25090 | *n.s.* | 1.8983 | *n.s.* | *n.s.* | *n.s.* | *n.s.* | -0.8972 |
| LOC_Os09g25690 | *n.s.* | *n.s.* | *n.s.* | *n.s.* | *n.s.* | -2.7299 | *n.s.* |
| LOC_Os09g25770 | *n.s.* | *n.s.* | *n.s.* | *n.s.* | *n.s.* | *n.s.* | -1.954 |
| LOC_Os09g26370 | *n.s.* | *n.s.* | 1.6535 | 1.9007 | *n.s.* | *n.s.* | 2.0697 |
| LOC_Os09g26880 | *n.s.* | *n.s.* | *n.s.* | *n.s.* | *n.s.* | *n.s.* | -0.783 |
| LOC_Os09g26920 | *n.s.* | *n.s.* | *n.s.* | *n.s.* | *n.s.* | -2.5972 | -1.7559 |
| LOC_Os09g27010 | 2.3247 | 1.1101 | *n.s.* | 1.0027 | *n.s.* | *n.s.* | *n.s.* |
| LOC_Os09g27330 | *n.s.* | *n.s.* | *n.s.* | *n.s.* | *n.s.* | -3.2827 | *n.s.* |
| LOC_Os09g27940 | 1.5475 | *n.s.* | *n.s.* | *n.s.* | *n.s.* | *n.s.* | *n.s.* |
| LOC_Os09g28160 | 1.6486 | *n.s.* | *n.s.* | 1.1146 | *n.s.* | -1.6758 | *n.s.* |
| LOC_Os09g28354 | *n.s.* | *n.s.* | *n.s.* | *n.s.* | -0.7328 | -1.2021 | *n.s.* |
| LOC_Os09g29940 | *n.s.* | *n.s.* | *n.s.* | *n.s.* | -1.0663 | -1.5043 | -0.9368 |
| LOC_Os09g30474 | *n.s.* | *n.s.* | *n.s.* | *n.s.* | *n.s.* | 0.6175 | *n.s.* |
| LOC_Os09g30490 | *n.s.* | *n.s.* | *n.s.* | *n.s.* | -1.7561 | -2.3151 | -3.4931 |
| LOC_Os09g31130 | *n.s.* | *n.s.* | -0.8977 | *n.s.* | *n.s.* | -1.0309 | *n.s.* |
| LOC_Os09g31200 | 0.6976 | *n.s.* | -0.8477 | *n.s.* | *n.s.* | -2.1726 | *n.s.* |
| LOC_Os09g31454 | 0.6113 | *n.s.* | *n.s.* | *n.s.* | *n.s.* | -0.6337 | *n.s.* |
| LOC_Os09g32010 | *n.s.* | *n.s.* | *n.s.* | *n.s.* | *n.s.* | -0.9043 | *n.s.* |
| LOC_Os09g32960 | *n.s.* | *n.s.* | *n.s.* | *n.s.* | *n.s.* | 0.8294 | *n.s.* |
| LOC_Os09g33530 | *n.s.* | *n.s.* | 0.8223 | *n.s.* | *n.s.* | -1.0056 | -2.081 |
| LOC_Os09g33690 | *n.s.* | *n.s.* | *n.s.* | *n.s.* | *n.s.* | -0.7705 | -0.9666 |
| LOC_Os09g33820 | -0.7666 | *n.s.* | *n.s.* | *n.s.* | *n.s.* | *n.s.* | *n.s.* |
| LOC_Os09g34890 | *n.s.* | *n.s.* | *n.s.* | *n.s.* | *n.s.* | *n.s.* | 1.7069 |
| LOC_Os09g36200 | *n.s.* | *n.s.* | *n.s.* | *n.s.* | *n.s.* | -1.0979 | *n.s.* |
| LOC_Os09g37100 | *n.s.* | *n.s.* | *n.s.* | *n.s.* | *n.s.* | 0.5128 | *n.s.* |
| LOC_Os09g37976 | *n.s.* | *n.s.* | -1.342 | -2.3186 | -1.2616 | -2.3481 | -3.1471 |
| LOC_Os09g38110 | *n.s.* | *n.s.* | *n.s.* | *n.s.* | *n.s.* | *n.s.* | -2.0811 |
| LOC_Os09g38130 | -0.9768 | *n.s.* | *n.s.* | *n.s.* | *n.s.* | *n.s.* | *n.s.* |
| LOC_Os09g38320 | *n.s.* | *n.s.* | *n.s.* | *n.s.* | -0.9638 | *n.s.* | *n.s.* |
| LOC_Os09g38410 | *n.s.* | *n.s.* | *n.s.* | *n.s.* | -0.6255 | *n.s.* | *n.s.* |
| LOC_Os09g38510 | *n.s.* | *n.s.* | 1.2011 | *n.s.* | *n.s.* | *n.s.* | *n.s.* |
| LOC_Os09g38920 | *n.s.* | *n.s.* | 0.9456 | *n.s.* | *n.s.* | *n.s.* | *n.s.* |
| LOC_Os09g39560 | *n.s.* | *n.s.* | *n.s.* | 0.7747 | *n.s.* | *n.s.* | 0.6864 |
| LOC_Os09g39620 | *n.s.* | *n.s.* | *n.s.* | *n.s.* | *n.s.* | -1.0748 | *n.s.* |
| LOC_Os09g39910 | *n.s.* | *n.s.* | *n.s.* | *n.s.* | *n.s.* | -0.7892 | -1.0187 |
| LOC_Os09g39940 | *n.s.* | 1.1867 | *n.s.* | *n.s.* | *n.s.* | -0.5817 | *n.s.* |
| LOC_Os10g02880 | *n.s.* | 2.3132 | *n.s.* | *n.s.* | *n.s.* | 2.1136 | *n.s.* |
| LOC_Os10g03320 | *n.s.* | *n.s.* | -0.5586 | *n.s.* | *n.s.* | -0.9177 | *n.s.* |
| LOC_Os10g04520 | *n.s.* | *n.s.* | -1.353 | *n.s.* | *n.s.* | -1.6295 | *n.s.* |
| LOC_Os10g04620 | *n.s.* | *n.s.* | -0.6634 | *n.s.* | *n.s.* | -0.6299 | *n.s.* |
| LOC_Os10g07010 | *n.s.* | *n.s.* | *n.s.* | *n.s.* | *n.s.* | -1.0646 | -4.2876 |
| LOC_Os10g14870 | *n.s.* | *n.s.* | 0.7271 | 1.0085 | *n.s.* | 1.2674 | 1.4215 |
| LOC_Os10g17489 | *n.s.* | *n.s.* | -1.6514 | *n.s.* | -1.3592 | -1.9687 | -1.1659 |
| LOC_Os10g20240 | *n.s.* | *n.s.* | *n.s.* | *n.s.* | *n.s.* | *n.s.* | 0.5948 |
| LOC_Os10g20470 | *n.s.* | *n.s.* | -0.8199 | *n.s.* | *n.s.* | -1.5567 | *n.s.* |
| LOC_Os10g22520 | *n.s.* | *n.s.* | *n.s.* | *n.s.* | *n.s.* | 0.8788 | *n.s.* |
| LOC_Os10g25010 | *n.s.* | *n.s.* | *n.s.* | *n.s.* | *n.s.* | *n.s.* | -0.9262 |
| LOC_Os10g25030 | *n.s.* | *n.s.* | 0.6258 | *n.s.* | 1.0774 | *n.s.* | -0.8199 |
| LOC_Os10g25210 | *n.s.* | *n.s.* | *n.s.* | *n.s.* | *n.s.* | -0.6532 | *n.s.* |
| LOC_Os10g26620 | *n.s.* | *n.s.* | -0.6497 | *n.s.* | *n.s.* | -1.3595 | -0.9363 |
| LOC_Os10g26680 | 0.7161 | *n.s.* | *n.s.* | *n.s.* | *n.s.* | -0.7905 | *n.s.* |
| LOC_Os10g27170 | *n.s.* | *n.s.* | *n.s.* | *n.s.* | *n.s.* | *n.s.* | -1.2769 |
| LOC_Os10g28000 | *n.s.* | *n.s.* | -0.9614 | *n.s.* | *n.s.* | -1.8164 | *n.s.* |
| LOC_Os10g30156 | *n.s.* | *n.s.* | *n.s.* | *n.s.* | *n.s.* | 1.2417 | 0.9871 |
| LOC_Os10g32444 | *n.s.* | *n.s.* | *n.s.* | *n.s.* | *n.s.* | -1.2893 | *n.s.* |
| LOC_Os10g32680 | *n.s.* | *n.s.* | *n.s.* | *n.s.* | 0.8879 | *n.s.* | -1.7467 |
| LOC_Os10g32810 | *n.s.* | 2.7292 | 1.3474 | 2.8309 | *n.s.* | *n.s.* | *n.s.* |
| LOC_Os10g33760 | *n.s.* | *n.s.* | 3.0188 | *n.s.* | *n.s.* | *n.s.* | *n.s.* |
| LOC_Os10g35070 | *n.s.* | *n.s.* | *n.s.* | *n.s.* | *n.s.* | *n.s.* | -1.2868 |
| LOC_Os10g36000 | -0.8552 | *n.s.* | *n.s.* | *n.s.* | *n.s.* | 1.7028 | *n.s.* |
| LOC_Os10g36180 | *n.s.* | 2.6545 | *n.s.* | *n.s.* | *n.s.* | -1.9129 | *n.s.* |
| LOC_Os10g36360 | *n.s.* | *n.s.* | *n.s.* | *n.s.* | -2.1274 | *n.s.* | *n.s.* |
| LOC_Os10g36924 | *n.s.* | *n.s.* | 0.7682 | *n.s.* | *n.s.* | 1.6892 | *n.s.* |
| LOC_Os10g37110 | *n.s.* | *n.s.* | *n.s.* | 0.938 | *n.s.* | *n.s.* | 0.6709 |
| LOC_Os10g38040 | *n.s.* | *n.s.* | *n.s.* | -1.2738 | *n.s.* | -0.638 | -2.2726 |
| LOC_Os10g38470 | *n.s.* | *n.s.* | *n.s.* | *n.s.* | *n.s.* | -0.6138 | *n.s.* |
| LOC_Os10g38489 | *n.s.* | *n.s.* | *n.s.* | *n.s.* | *n.s.* | -0.866 | *n.s.* |
| LOC_Os10g38740 | *n.s.* | *n.s.* | *n.s.* | *n.s.* | *n.s.* | -2.3995 | *n.s.* |
| LOC_Os10g38820 | 1.7968 | *n.s.* | *n.s.* | *n.s.* | -2.3764 | *n.s.* | *n.s.* |
| LOC_Os10g39470 | *n.s.* | *n.s.* | *n.s.* | 1.8087 | *n.s.* | 0.9471 | 1.2945 |
| LOC_Os10g39920 | *n.s.* | *n.s.* | *n.s.* | *n.s.* | *n.s.* | -1.059 | *n.s.* |
| LOC_Os10g40490 | *n.s.* | *n.s.* | -0.7177 | *n.s.* | *n.s.* | -1.2344 | *n.s.* |
| LOC_Os10g40600 | *n.s.* | *n.s.* | *n.s.* | *n.s.* | *n.s.* | 1.4286 | *n.s.* |
| LOC_Os10g41550 | *n.s.* | *n.s.* | *n.s.* | -4.0957 | *n.s.* | *n.s.* | -4.3846 |
| LOC_Os10g41560 | *n.s.* | *n.s.* | *n.s.* | *n.s.* | *n.s.* | 0.5032 | *n.s.* |
| LOC_Os10g41930 | *n.s.* | *n.s.* | -0.9632 | *n.s.* | *n.s.* | -0.9225 | *n.s.* |
| LOC_Os10g42190 | *n.s.* | *n.s.* | *n.s.* | *n.s.* | *n.s.* | -0.6549 | *n.s.* |
| LOC_Os10g42610 | *n.s.* | *n.s.* | *n.s.* | *n.s.* | *n.s.* | -1.9957 | -1.2851 |
| LOC_Os11g02820 | *n.s.* | *n.s.* | *n.s.* | *n.s.* | -0.8415 | -0.712 | *n.s.* |
| LOC_Os11g03300 | *n.s.* | *n.s.* | *n.s.* | *n.s.* | *n.s.* | -3.1191 | -2.2921 |
| LOC_Os11g03780 | *n.s.* | *n.s.* | *n.s.* | *n.s.* | 1.899 | *n.s.* | *n.s.* |
| LOC_Os11g04104 | *n.s.* | *n.s.* | *n.s.* | *n.s.* | *n.s.* | -1.3564 | -0.8525 |
| LOC_Os11g05400 | *n.s.* | *n.s.* | 0.8006 | 1.0322 | *n.s.* | 1.3362 | 0.9857 |
| LOC_Os11g05470 | *n.s.* | *n.s.* | *n.s.* | *n.s.* | *n.s.* | -1.9401 | *n.s.* |
| LOC_Os11g05640 | *n.s.* | *n.s.* | *n.s.* | *n.s.* | *n.s.* | *n.s.* | -2.349 |
| LOC_Os11g06130 | *n.s.* | *n.s.* | *n.s.* | *n.s.* | -1.095 | -0.6576 | *n.s.* |
| LOC_Os11g06770 | *n.s.* | *n.s.* | -1.0399 | -1.2461 | -0.7986 | -2.7976 | -2.0734 |
| LOC_Os11g07600 | *n.s.* | *n.s.* | *n.s.* | *n.s.* | *n.s.* | -0.6538 | *n.s.* |
| LOC_Os11g09020 | *n.s.* | *n.s.* | 1.0455 | *n.s.* | *n.s.* | *n.s.* | -1.3767 |
| LOC_Os11g10480 | *n.s.* | *n.s.* | *n.s.* | -1.1971 | *n.s.* | -1.319 | -1.6324 |
| LOC_Os11g10760 | 1.0199 | *n.s.* | *n.s.* | *n.s.* | -0.795 | *n.s.* | *n.s.* |
| LOC_Os11g11694 | *n.s.* | *n.s.* | *n.s.* | *n.s.* | -0.943 | *n.s.* | *n.s.* |
| LOC_Os11g13670 | *n.s.* | *n.s.* | *n.s.* | *n.s.* | *n.s.* | -1.0672 | *n.s.* |
| LOC_Os11g18570 | -1.5921 | *n.s.* | *n.s.* | *n.s.* | *n.s.* | *n.s.* | *n.s.* |
| LOC_Os11g25780 | *n.s.* | *n.s.* | *n.s.* | *n.s.* | *n.s.* | 0.7343 | *n.s.* |
| LOC_Os11g26570 | *n.s.* | *n.s.* | *n.s.* | *n.s.* | *n.s.* | *n.s.* | -6.3742 |
| LOC_Os11g26760 | *n.s.* | *n.s.* | *n.s.* | *n.s.* | *n.s.* | *n.s.* | -3.7403 |
| LOC_Os11g26790 | *n.s.* | *n.s.* | *n.s.* | -6.1704 | *n.s.* | *n.s.* | -5.2771 |
| LOC_Os11g29780 | *n.s.* | *n.s.* | *n.s.* | *n.s.* | *n.s.* | -2.8402 | *n.s.* |
| LOC_Os11g30500 | *n.s.* | 2.0174 | *n.s.* | *n.s.* | -1.2548 | *n.s.* | *n.s.* |
| LOC_Os11g35040 | *n.s.* | *n.s.* | *n.s.* | *n.s.* | *n.s.* | *n.s.* | 1.1743 |
| LOC_Os11g35320 | *n.s.* | *n.s.* | -0.6867 | *n.s.* | *n.s.* | -0.6718 | *n.s.* |
| LOC_Os11g37260 | *n.s.* | *n.s.* | *n.s.* | *n.s.* | *n.s.* | -0.518 | *n.s.* |
| LOC_Os11g37560 | *n.s.* | *n.s.* | *n.s.* | *n.s.* | *n.s.* | *n.s.* | -1.1747 |
| LOC_Os11g37950 | *n.s.* | *n.s.* | -3.9111 | *n.s.* | *n.s.* | -1.6328 | -2.072 |
| LOC_Os11g41600 | 0.7215 | *n.s.* | *n.s.* | *n.s.* | *n.s.* | -0.9333 | *n.s.* |
| LOC_Os11g41650 | *n.s.* | *n.s.* | *n.s.* | *n.s.* | *n.s.* | -2.9838 | *n.s.* |
| LOC_Os11g42510 | -1.4362 | *n.s.* | *n.s.* | *n.s.* | *n.s.* | *n.s.* | *n.s.* |
| LOC_Os11g43790 | *n.s.* | *n.s.* | *n.s.* | *n.s.* | *n.s.* | -3.713 | -7.6967 |
| LOC_Os11g44800 | *n.s.* | *n.s.* | 2.7909 | *n.s.* | *n.s.* | *n.s.* | *n.s.* |
| LOC_Os11g45740 | *n.s.* | *n.s.* | *n.s.* | *n.s.* | *n.s.* | -1.1538 | *n.s.* |
| LOC_Os11g47600 | *n.s.* | -2.4396 | *n.s.* | *n.s.* | *n.s.* | -1.6328 | *n.s.* |
| LOC_Os11g47930 | *n.s.* | *n.s.* | *n.s.* | *n.s.* | *n.s.* | 1.0034 | 0.8768 |
| LOC_Os11g48020 | *n.s.* | *n.s.* | *n.s.* | *n.s.* | *n.s.* | 0.6764 | *n.s.* |
| LOC_Os12g02080 | 1.3123 | *n.s.* | *n.s.* | *n.s.* | -1.138 | *n.s.* | *n.s.* |
| LOC_Os12g02800 | *n.s.* | *n.s.* | *n.s.* | *n.s.* | *n.s.* | -0.946 | *n.s.* |
| LOC_Os12g03860 | *n.s.* | *n.s.* | *n.s.* | *n.s.* | *n.s.* | 1.6242 | *n.s.* |
| LOC_Os12g03899 | *n.s.* | *n.s.* | *n.s.* | *n.s.* | *n.s.* | -1.1192 | *n.s.* |
| LOC_Os12g04204 | *n.s.* | *n.s.* | *n.s.* | *n.s.* | *n.s.* | 1.0862 | *n.s.* |
| LOC_Os12g05050 | 0.8264 | *n.s.* | *n.s.* | 0.6956 | 0.6418 | *n.s.* | 0.7788 |
| LOC_Os12g05210 | *n.s.* | 3.4062 | *n.s.* | *n.s.* | *n.s.* | *n.s.* | *n.s.* |
| LOC_Os12g07030 | 1.661 | *n.s.* | *n.s.* | *n.s.* | -0.7422 | -0.7039 | -1.6264 |
| LOC_Os12g08700 | *n.s.* | *n.s.* | *n.s.* | -4.8087 | -2.048 | *n.s.* | -8.1978 |
| LOC_Os12g12260 | 1.117 | *n.s.* | -0.6201 | *n.s.* | *n.s.* | -0.832 | *n.s.* |
| LOC_Os12g12370 | *n.s.* | *n.s.* | *n.s.* | *n.s.* | *n.s.* | 0.6399 | *n.s.* |
| LOC_Os12g12880 | *n.s.* | *n.s.* | *n.s.* | *n.s.* | *n.s.* | -2.2827 | -2.2349 |
| LOC_Os12g13640 | *n.s.* | *n.s.* | *n.s.* | *n.s.* | *n.s.* | -1.2692 | -4.6481 |
| LOC_Os12g13940 | *n.s.* | *n.s.* | 1.3747 | 2.668 | *n.s.* | 1.9076 | *n.s.* |
| LOC_Os12g16720 | *n.s.* | *n.s.* | *n.s.* | *n.s.* | *n.s.* | -2.9257 | -2.112 |
| LOC_Os12g21789 | *n.s.* | *n.s.* | *n.s.* | *n.s.* | *n.s.* | -0.5571 | *n.s.* |
| LOC_Os12g24170 | *n.s.* | *n.s.* | *n.s.* | *n.s.* | 0.856 | 0.6186 | *n.s.* |
| LOC_Os12g29330 | 0.9262 | *n.s.* | 0.614 | *n.s.* | *n.s.* | *n.s.* | *n.s.* |
| LOC_Os12g29400 | *n.s.* | *n.s.* | *n.s.* | *n.s.* | *n.s.* | -1.7403 | -1.8592 |
| LOC_Os12g31860 | -6.1495 | *n.s.* | *n.s.* | *n.s.* | *n.s.* | *n.s.* | *n.s.* |
| LOC_Os12g32610 | *n.s.* | *n.s.* | *n.s.* | *n.s.* | *n.s.* | *n.s.* | -2.1679 |
| LOC_Os12g32970 | *n.s.* | *n.s.* | *n.s.* | *n.s.* | *n.s.* | -1.2045 | *n.s.* |
| LOC_Os12g37690 | *n.s.* | *n.s.* | *n.s.* | *n.s.* | *n.s.* | -1.6549 | -2.3764 |
| LOC_Os12g38150 | -2.1607 | *n.s.* | *n.s.* | *n.s.* | *n.s.* | -0.9386 | *n.s.* |
| LOC_Os12g40419 | *n.s.* | *n.s.* | 1.2032 | *n.s.* | *n.s.* | *n.s.* | *n.s.* |
| LOC_Os12g43000 | -1.2738 | *n.s.* | *n.s.* | *n.s.* | *n.s.* | *n.s.* | *n.s.* |
| LOC_Os12g43140 | *n.s.* | *n.s.* | *n.s.* | *n.s.* | *n.s.* | -3.5511 | *n.s.* |
| LOC_Os12g43720 | *n.s.* | *n.s.* | *n.s.* | *n.s.* | -1.2365 | *n.s.* | *n.s.* |
| LOC_Os12g43880 | *n.s.* | *n.s.* | *n.s.* | *n.s.* | *n.s.* | -0.5727 | *n.s.* |
| LOC_Os12g44100 | -1.3315 | *n.s.* | *n.s.* | *n.s.* | -1.3349 | *n.s.* | *n.s.* |
| LOC_Os12g44380 | *n.s.* | *n.s.* | *n.s.* | *n.s.* | -0.6298 | *n.s.* | *n.s.* |

Numeric values are log_2_ fold change high temperature relative to normal temperature; *n.s.* indicates the gene was not significantly differentially expressed. Genes are up-regulated following ABA treatment, identified from Garg R, *et al.* (Plant Signaling & Behavior. 2012;7(8):951-6).
