# Supplementary material for: RNA-Seq analysis reveals insight into enhanced rice Xa7-mediated bacterial blight resistance at high temperature
Source: PLoS One. 2017 Nov 6;12(11):e0187625. doi: 10.1371/journal.pone.0187625 (PMC5673197; doi:10.1371/journal.pone.0187625)
Supplement: S7 Table — (DOCX) [file pone.0187625.s009.docx]

**Table S7: Differential expression analysis of SA-responsive genes.**

|  | **Mock** | **Susceptible Interaction** | | | **Resistant Interaction** | | |
| --- | --- | --- | --- | --- | --- | --- | --- |
| **Locus** | **6 hpi** | **3 hpi** | **12 hpi** | **24 hpi** | **3 hpi** | **12 hpi** | **24 hpi** |
| LOC_Os01g02390 | n.s. | n.s. | n.s. | n.s. | 0.7036 | n.s. | n.s. |
| LOC_Os01g02400 | n.s. | n.s. | n.s. | n.s. | n.s. | 0.5059 | n.s. |
| LOC_Os01g03144 | n.s. | n.s. | n.s. | n.s. | n.s. | 0.6489 | n.s. |
| LOC_Os01g08860 | n.s. | n.s. | 2.7673 | n.s. | n.s. | n.s. | 1.5399 |
| LOC_Os01g10440 | 1.454 | n.s. | n.s. | n.s. | n.s. | n.s. | n.s. |
| LOC_Os01g12820 | n.s. | n.s. | n.s. | n.s. | -1.0939 | -2.4277 | n.s. |
| LOC_Os01g13950 | n.s. | n.s. | n.s. | n.s. | n.s. | -4.9125 | n.s. |
| LOC_Os01g16146 | n.s. | n.s. | n.s. | n.s. | n.s. | n.s. | 0.7745 |
| LOC_Os01g19130 | n.s. | n.s. | n.s. | n.s. | -0.7085 | n.s. | n.s. |
| LOC_Os01g27210 | n.s. | n.s. | -1.9194 | n.s. | n.s. | -1.8669 | 0.722 |
| LOC_Os01g27750 | n.s. | n.s. | n.s. | n.s. | n.s. | -0.787 | n.s. |
| LOC_Os01g28840 | n.s. | n.s. | n.s. | -6.9188 | n.s. | -2.3203 | -6.9483 |
| LOC_Os01g31980 | n.s. | n.s. | n.s. | n.s. | n.s. | -0.7721 | -0.9676 |
| LOC_Os01g32780 | n.s. | n.s. | -0.8908 | n.s. | n.s. | -1.5195 | -1.8631 |
| LOC_Os01g34480 | n.s. | n.s. | n.s. | 0.7486 | n.s. | n.s. | 0.7225 |
| LOC_Os01g39136 | n.s. | n.s. | -1.0779 | n.s. | n.s. | -1.6877 | n.s. |
| LOC_Os01g40094 | n.s. | n.s. | n.s. | n.s. | -1.3457 | -0.6588 | -0.7842 |
| LOC_Os01g40280 | n.s. | n.s. | n.s. | -1.4391 | -1.1313 | -1.2053 | -1.774 |
| LOC_Os01g41240 | n.s. | n.s. | n.s. | n.s. | n.s. | -1.0275 | n.s. |
| LOC_Os01g41430 | 2.0036 | n.s. | -1.4437 | 1.3916 | n.s. | -1.303 | 1.3683 |
| LOC_Os01g42380 | n.s. | n.s. | n.s. | n.s. | n.s. | -1.5014 | -1.2703 |
| LOC_Os01g43650 | n.s. | n.s. | n.s. | n.s. | -0.8931 | n.s. | -1.3573 |
| LOC_Os01g43700 | -1.4154 | -1.8174 | -2.6308 | n.s. | -1.4416 | -1.2214 | n.s. |
| LOC_Os01g44050 | n.s. | n.s. | -1.5988 | -1.2916 | -1.0758 | n.s. | -1.5444 |
| LOC_Os01g47300 | n.s. | n.s. | -0.9281 | n.s. | n.s. | -2.1824 | -2.2852 |
| LOC_Os01g47460 | n.s. | n.s. | -0.7066 | n.s. | n.s. | n.s. | n.s. |
| LOC_Os01g47690 | n.s. | n.s. | -0.8464 | n.s. | 0.895 | -0.9117 | n.s. |
| LOC_Os01g47840 | n.s. | n.s. | n.s. | n.s. | 0.8343 | 0.6675 | n.s. |
| LOC_Os01g49720 | n.s. | n.s. | n.s. | 1.4162 | n.s. | n.s. | n.s. |
| LOC_Os01g50050 | n.s. | n.s. | n.s. | n.s. | -1.1406 | n.s. | n.s. |
| LOC_Os01g51870 | n.s. | n.s. | -0.9539 | n.s. | n.s. | n.s. | -0.6943 |
| LOC_Os01g54890 | n.s. | n.s. | n.s. | 1.1696 | n.s. | n.s. | 1.4106 |
| LOC_Os01g55160 | n.s. | n.s. | -1.7409 | n.s. | n.s. | -1.8758 | n.s. |
| LOC_Os01g55940 | n.s. | n.s. | n.s. | 1.8081 | n.s. | n.s. | 0.9719 |
| LOC_Os01g59100 | n.s. | n.s. | -1.2629 | n.s. | n.s. | -0.739 | n.s. |
| LOC_Os01g62190 | n.s. | n.s. | n.s. | n.s. | n.s. | -1.7322 | n.s. |
| LOC_Os01g62610 | -2.434 | n.s. | n.s. | n.s. | n.s. | n.s. | -1.2369 |
| LOC_Os01g62760 | n.s. | 1.4347 | n.s. | n.s. | -1.0217 | -1.0849 | n.s. |
| LOC_Os01g63980 | n.s. | n.s. | n.s. | n.s. | n.s. | -1.0668 | n.s. |
| LOC_Os01g68570 | n.s. | n.s. | -1.2109 | -2.1697 | n.s. | n.s. | -1.8878 |
| LOC_Os01g72130 | n.s. | n.s. | -1.1279 | n.s. | n.s. | -1.2269 | n.s. |
| LOC_Os01g72170 | n.s. | n.s. | n.s. | n.s. | n.s. | -0.8621 | n.s. |
| LOC_Os01g72530 | 3.8103 | n.s. | n.s. | n.s. | n.s. | -2.2331 | -0.9848 |
| LOC_Os01g72970 | n.s. | n.s. | n.s. | n.s. | -0.88 | -1.7992 | -1.1865 |
| LOC_Os02g03740 | n.s. | n.s. | -0.5852 | n.s. | -0.7459 | n.s. | n.s. |
| LOC_Os02g09810 | n.s. | n.s. | n.s. | n.s. | n.s. | -0.6782 | -2.577 |
| LOC_Os02g10070 | n.s. | n.s. | -0.4951 | n.s. | n.s. | n.s. | n.s. |
| LOC_Os02g12350 | n.s. | n.s. | n.s. | n.s. | n.s. | -1.8315 | n.s. |
| LOC_Os02g13380 | n.s. | n.s. | -0.5585 | n.s. | n.s. | -0.498 | -0.6992 |
| LOC_Os02g17390 | n.s. | n.s. | n.s. | n.s. | n.s. | -0.7 | n.s. |
| LOC_Os02g32140 | n.s. | n.s. | n.s. | n.s. | n.s. | -1.4313 | -1.0769 |
| LOC_Os02g36340 | n.s. | n.s. | n.s. | n.s. | n.s. | 0.6567 | n.s. |
| LOC_Os02g40500 | n.s. | n.s. | n.s. | n.s. | n.s. | -1.153 | n.s. |
| LOC_Os02g43330 | n.s. | 3.5259 | n.s. | -5.4619 | n.s. | -3.8751 | -5.9295 |
| LOC_Os02g44770 | n.s. | n.s. | n.s. | n.s. | n.s. | -1.9366 | n.s. |
| LOC_Os02g44990 | n.s. | n.s. | n.s. | n.s. | n.s. | -1.5715 | -1.6276 |
| LOC_Os02g47650 | 1.0695 | n.s. | n.s. | n.s. | n.s. | n.s. | n.s. |
| LOC_Os02g50690 | n.s. | -1.8885 | n.s. | n.s. | n.s. | -2.11 | -3.1802 |
| LOC_Os02g50730 | n.s. | n.s. | n.s. | n.s. | n.s. | -1.0301 | n.s. |
| LOC_Os02g50805 | n.s. | n.s. | n.s. | n.s. | n.s. | -1.5449 | n.s. |
| LOC_Os02g51350 | 2.0409 | n.s. | n.s. | n.s. | -1.3538 | -1.9578 | -1.7168 |
| LOC_Os02g51890 | n.s. | n.s. | n.s. | n.s. | n.s. | -1.3479 | n.s. |
| LOC_Os02g56310 | n.s. | n.s. | n.s. | n.s. | n.s. | n.s. | 1.0885 |
| LOC_Os02g56700 | n.s. | n.s. | n.s. | n.s. | n.s. | -3.7923 | n.s. |
| LOC_Os03g02874 | n.s. | n.s. | -0.7654 | n.s. | n.s. | -2.0183 | -1.7534 |
| LOC_Os03g04410 | n.s. | n.s. | n.s. | n.s. | n.s. | -0.6185 | n.s. |
| LOC_Os03g04890 | n.s. | n.s. | n.s. | n.s. | -1.3978 | -1.7527 | n.s. |
| LOC_Os03g05910 | 0.9298 | n.s. | -1.6819 | n.s. | 1.3359 | n.s. | n.s. |
| LOC_Os03g06200 | n.s. | n.s. | -0.7759 | n.s. | n.s. | -1.8964 | n.s. |
| LOC_Os03g08900 | n.s. | n.s. | -1.3606 | n.s. | -1.7313 | -1.6307 | n.s. |
| LOC_Os03g12510 | n.s. | n.s. | -3.4921 | n.s. | n.s. | n.s. | n.s. |
| LOC_Os03g13300 | n.s. | n.s. | 1.5298 | n.s. | -1.0161 | n.s. | -2.1681 |
| LOC_Os03g17470 | n.s. | n.s. | 0.7605 | 1.3312 | n.s. | 0.9211 | 1.5319 |
| LOC_Os03g17480 | n.s. | n.s. | n.s. | 2.645 | n.s. | n.s. | 2.8928 |
| LOC_Os03g19250 | n.s. | n.s. | n.s. | n.s. | 1.2036 | n.s. | n.s. |
| LOC_Os03g19370 | 3.298 | -2.2166 | -3.1205 | n.s. | -1.7729 | -3.8943 | n.s. |
| LOC_Os03g20870 | n.s. | n.s. | 0.7446 | n.s. | n.s. | n.s. | n.s. |
| LOC_Os03g21710 | 2.286 | n.s. | n.s. | n.s. | n.s. | n.s. | n.s. |
| LOC_Os03g22200 | n.s. | n.s. | 0.9857 | n.s. | n.s. | n.s. | n.s. |
| LOC_Os03g29190 | n.s. | n.s. | n.s. | n.s. | n.s. | n.s. | 0.964 |
| LOC_Os03g29240 | n.s. | n.s. | -0.6989 | n.s. | n.s. | -1.0549 | n.s. |
| LOC_Os03g31750 | n.s. | n.s. | -1.5139 | n.s. | n.s. | n.s. | n.s. |
| LOC_Os03g37490 | n.s. | n.s. | n.s. | n.s. | n.s. | -1.0521 | n.s. |
| LOC_Os03g43720 | n.s. | n.s. | n.s. | n.s. | n.s. | n.s. | -0.8477 |
| LOC_Os03g50130 | n.s. | -1.6296 | -1.1416 | n.s. | -1.1422 | -0.7722 | 1.023 |
| LOC_Os03g50960 | n.s. | n.s. | n.s. | n.s. | n.s. | -1.7321 | n.s. |
| LOC_Os03g51390 | n.s. | n.s. | n.s. | n.s. | n.s. | -1.2702 | n.s. |
| LOC_Os03g52380 | n.s. | n.s. | n.s. | n.s. | n.s. | -1.4124 | -2.1773 |
| LOC_Os03g55240 | n.s. | n.s. | n.s. | 1.2609 | n.s. | n.s. | 1.3641 |
| LOC_Os03g55290 | n.s. | n.s. | n.s. | n.s. | n.s. | n.s. | -4.0542 |
| LOC_Os03g57200 | n.s. | n.s. | -2.2507 | 2.7186 | n.s. | -2.6361 | 1.9592 |
| LOC_Os03g58790 | 1.3153 | n.s. | -1.3039 | n.s. | n.s. | n.s. | n.s. |
| LOC_Os03g58800 | n.s. | n.s. | n.s. | n.s. | n.s. | -2.6947 | -3.0293 |
| LOC_Os03g59320 | n.s. | n.s. | n.s. | n.s. | -1.1206 | n.s. | n.s. |
| LOC_Os03g60370 | n.s. | n.s. | -0.6877 | n.s. | -0.9658 | -1.0504 | -1.0577 |
| LOC_Os03g60570 | 5.7132 | n.s. | n.s. | n.s. | n.s. | -1.9774 | -2.2691 |
| LOC_Os03g60580 | n.s. | n.s. | n.s. | -2.5948 | n.s. | -1.9932 | -3.3147 |
| LOC_Os03g61360 | n.s. | n.s. | -2.4518 | n.s. | n.s. | -2.2968 | n.s. |
| LOC_Os03g62480 | n.s. | n.s. | n.s. | n.s. | n.s. | n.s. | 2.4058 |
| LOC_Os03g63620 | n.s. | n.s. | n.s. | n.s. | n.s. | n.s. | 4.6164 |
| LOC_Os04g06520 | n.s. | n.s. | n.s. | n.s. | -1.2009 | n.s. | n.s. |
| LOC_Os04g06590 | n.s. | n.s. | n.s. | n.s. | n.s. | -2.6166 | -2.4957 |
| LOC_Os04g13210 | n.s. | n.s. | -1.2211 | n.s. | n.s. | -1.9097 | n.s. |
| LOC_Os04g27060 | n.s. | n.s. | -2.4689 | n.s. | n.s. | n.s. | n.s. |
| LOC_Os04g30420 | 1.1515 | n.s. | n.s. | n.s. | n.s. | n.s. | 0.6987 |
| LOC_Os04g32620 | n.s. | n.s. | n.s. | n.s. | n.s. | -0.9064 | n.s. |
| LOC_Os04g33240 | n.s. | n.s. | n.s. | n.s. | n.s. | n.s. | -1.474 |
| LOC_Os04g33490 | 0.7251 | n.s. | n.s. | n.s. | n.s. | -0.8219 | n.s. |
| LOC_Os04g35130 | 1.1331 | n.s. | n.s. | n.s. | n.s. | -1.542 | -1.0779 |
| LOC_Os04g35540 | n.s. | n.s. | n.s. | n.s. | n.s. | -1.3796 | -1.4487 |
| LOC_Os04g37490 | n.s. | n.s. | -1.118 | n.s. | -1.5722 | -1.6561 | n.s. |
| LOC_Os04g37710 | n.s. | n.s. | n.s. | n.s. | -0.7476 | n.s. | n.s. |
| LOC_Os04g40310 | n.s. | n.s. | -0.6444 | n.s. | n.s. | -1.3143 | -1.0044 |
| LOC_Os04g47140 | n.s. | n.s. | n.s. | 1.3395 | n.s. | n.s. | n.s. |
| LOC_Os04g47720 | n.s. | n.s. | n.s. | n.s. | n.s. | -1.6664 | n.s. |
| LOC_Os04g49210 | n.s. | n.s. | n.s. | n.s. | -1.6127 | -2.431 | n.s. |
| LOC_Os04g51160 | n.s. | n.s. | n.s. | n.s. | n.s. | -1.9618 | n.s. |
| LOC_Os04g51460 | 4.9642 | n.s. | n.s. | n.s. | -2.5661 | -3.3708 | n.s. |
| LOC_Os04g52504 | n.s. | n.s. | n.s. | n.s. | -0.6285 | -1.3696 | -1.082 |
| LOC_Os04g55700 | n.s. | n.s. | n.s. | n.s. | n.s. | -0.6972 | n.s. |
| LOC_Os04g55720 | n.s. | n.s. | n.s. | n.s. | n.s. | -1.8678 | -0.9091 |
| LOC_Os04g57550 | 1.3471 | n.s. | -0.5676 | n.s. | -0.7783 | -1.2762 | n.s. |
| LOC_Os04g57810 | 1.4254 | n.s. | -0.6566 | n.s. | -1.3105 | -1.4766 | -0.8922 |
| LOC_Os05g01444 | n.s. | n.s. | n.s. | n.s. | n.s. | -1.4673 | n.s. |
| LOC_Os05g02770 | n.s. | n.s. | n.s. | n.s. | n.s. | -0.7438 | n.s. |
| LOC_Os05g03820 | n.s. | n.s. | n.s. | n.s. | n.s. | 0.7806 | n.s. |
| LOC_Os05g10650 | n.s. | n.s. | n.s. | n.s. | n.s. | -3.6306 | n.s. |
| LOC_Os05g10730 | n.s. | n.s. | n.s. | n.s. | n.s. | -0.6441 | n.s. |
| LOC_Os05g33960 | n.s. | n.s. | n.s. | n.s. | n.s. | -1.9141 | -3.0117 |
| LOC_Os05g34830 | n.s. | n.s. | -0.6758 | n.s. | n.s. | -1.1902 | n.s. |
| LOC_Os05g38290 | n.s. | n.s. | n.s. | n.s. | -1.9794 | -2.1383 | -1.4435 |
| LOC_Os05g41490 | n.s. | 2.4269 | n.s. | n.s. | n.s. | -3.3008 | n.s. |
| LOC_Os05g48650 | n.s. | n.s. | n.s. | -1.7498 | n.s. | n.s. | n.s. |
| LOC_Os05g51670 | 0.915 | n.s. | n.s. | n.s. | n.s. | -0.8616 | n.s. |
| LOC_Os06g01350 | n.s. | n.s. | n.s. | 1.0973 | n.s. | n.s. | n.s. |
| LOC_Os06g04240 | 2.8361 | n.s. | n.s. | n.s. | n.s. | n.s. | -1.3053 |
| LOC_Os06g05420 | n.s. | n.s. | n.s. | n.s. | -1.0622 | n.s. | -3.2075 |
| LOC_Os06g05470 | n.s. | n.s. | n.s. | n.s. | n.s. | -1.7551 | n.s. |
| LOC_Os06g11240 | n.s. | n.s. | -0.6223 | n.s. | n.s. | -0.9479 | n.s. |
| LOC_Os06g11290 | n.s. | n.s. | -1.7975 | n.s. | -1.2844 | -2.5672 | n.s. |
| LOC_Os06g20820 | n.s. | n.s. | n.s. | n.s. | n.s. | n.s. | -1.8576 |
| LOC_Os06g39330 | n.s. | n.s. | 1.2543 | n.s. | n.s. | 1.3668 | n.s. |
| LOC_Os06g42560 | n.s. | n.s. | n.s. | n.s. | n.s. | -1.0701 | -1.0715 |
| LOC_Os06g47200 | n.s. | n.s. | n.s. | n.s. | n.s. | n.s. | -1.4579 |
| LOC_Os06g48200 | 1.6254 | n.s. | n.s. | n.s. | n.s. | n.s. | n.s. |
| LOC_Os06g48810 | n.s. | 3.0253 | 1.871 | n.s. | n.s. | 1.9127 | n.s. |
| LOC_Os06g50920 | n.s. | -1.6129 | n.s. | n.s. | n.s. | 1.1922 | n.s. |
| LOC_Os07g09420 | n.s. | n.s. | -0.9491 | n.s. | n.s. | -1.9368 | -1.5927 |
| LOC_Os07g12340 | 1.2098 | n.s. | n.s. | 1.94 | 1.0417 | -1.5491 | 1.2197 |
| LOC_Os07g17330 | 1.4377 | n.s. | n.s. | n.s. | n.s. | -1.143 | -0.9542 |
| LOC_Os07g22730 | n.s. | n.s. | -2.5777 | n.s. | n.s. | -4.0904 | n.s. |
| LOC_Os07g23570 | n.s. | n.s. | n.s. | n.s. | n.s. | n.s. | -4.6353 |
| LOC_Os07g37730 | n.s. | n.s. | n.s. | n.s. | n.s. | -2.1545 | -1.3803 |
| LOC_Os07g39520 | n.s. | n.s. | n.s. | n.s. | -0.9572 | -1.3789 | n.s. |
| LOC_Os07g44140 | n.s. | n.s. | -2.193 | n.s. | n.s. | -2.348 | n.s. |
| LOC_Os07g44410 | -1.3717 | n.s. | n.s. | n.s. | n.s. | -0.8404 | -2.5495 |
| LOC_Os07g47590 | n.s. | n.s. | n.s. | n.s. | n.s. | 0.8215 | -0.7527 |
| LOC_Os07g47670 | n.s. | n.s. | -1.4306 | n.s. | n.s. | -2.1464 | n.s. |
| LOC_Os07g49460 | n.s. | n.s. | -1.6593 | n.s. | n.s. | -2.1959 | n.s. |
| LOC_Os08g01760 | n.s. | n.s. | n.s. | n.s. | n.s. | 0.5797 | n.s. |
| LOC_Os08g02030 | n.s. | n.s. | n.s. | n.s. | n.s. | -1.6688 | -2.8241 |
| LOC_Os08g06210 | n.s. | n.s. | n.s. | n.s. | n.s. | n.s. | -2.3722 |
| LOC_Os08g27720 | n.s. | n.s. | n.s. | n.s. | n.s. | -0.7301 | n.s. |
| LOC_Os08g33710 | n.s. | n.s. | n.s. | n.s. | n.s. | -1.4756 | -2.0011 |
| LOC_Os08g36860 | n.s. | 1.3253 | n.s. | n.s. | n.s. | n.s. | 1.3592 |
| LOC_Os08g37432 | n.s. | n.s. | -1.4156 | n.s. | -1.4392 | -1.3131 | n.s. |
| LOC_Os08g37840 | n.s. | n.s. | n.s. | n.s. | n.s. | -1.8079 | -1.7481 |
| LOC_Os08g37874 | n.s. | n.s. | n.s. | n.s. | n.s. | -0.9639 | n.s. |
| LOC_Os08g38600 | n.s. | n.s. | n.s. | n.s. | n.s. | -2.4015 | -1.8414 |
| LOC_Os08g39730 | n.s. | n.s. | n.s. | n.s. | n.s. | -2.4888 | -5.9734 |
| LOC_Os08g40590 | n.s. | n.s. | n.s. | n.s. | n.s. | -1.1768 | n.s. |
| LOC_Os08g40680 | n.s. | n.s. | n.s. | n.s. | n.s. | -2.7473 | n.s. |
| LOC_Os08g40940 | n.s. | n.s. | n.s. | n.s. | n.s. | -2.2758 | n.s. |
| LOC_Os08g44340 | 1.4887 | n.s. | -0.8329 | n.s. | -1.1124 | -1.6237 | n.s. |
| LOC_Os08g45120 | n.s. | n.s. | n.s. | n.s. | n.s. | -2.4016 | n.s. |
| LOC_Os09g03190 | n.s. | n.s. | -1.4405 | n.s. | n.s. | -2.0118 | n.s. |
| LOC_Os09g15320 | n.s. | n.s. | n.s. | -1.6226 | n.s. | n.s. | -1.4925 |
| LOC_Os09g15670 | n.s. | 1.44 | n.s. | n.s. | -1.2342 | -2.2807 | n.s. |
| LOC_Os09g16330 | -0.7667 | -1.4641 | -1.698 | n.s. | n.s. | -0.9376 | n.s. |
| LOC_Os09g20220 | n.s. | n.s. | -3.8667 | n.s. | n.s. | -3.0256 | n.s. |
| LOC_Os09g21710 | 2.0803 | n.s. | n.s. | n.s. | n.s. | n.s. | n.s. |
| LOC_Os09g25070 | n.s. | n.s. | n.s. | n.s. | n.s. | n.s. | 4.0271 |
| LOC_Os09g25090 | n.s. | 1.8983 | n.s. | n.s. | n.s. | n.s. | -0.8972 |
| LOC_Os09g25770 | n.s. | n.s. | n.s. | n.s. | n.s. | n.s. | -1.954 |
| LOC_Os09g27940 | 1.5475 | n.s. | n.s. | n.s. | n.s. | n.s. | n.s. |
| LOC_Os09g28160 | 1.6486 | n.s. | n.s. | 1.1146 | n.s. | -1.6758 | n.s. |
| LOC_Os09g30490 | n.s. | n.s. | n.s. | n.s. | -1.7561 | -2.3151 | -3.4931 |
| LOC_Os09g31130 | n.s. | n.s. | -0.8977 | n.s. | n.s. | -1.0309 | n.s. |
| LOC_Os09g34250 | n.s. | n.s. | -4.0288 | 2.2409 | n.s. | -3.8084 | 2.3272 |
| LOC_Os09g37976 | n.s. | n.s. | -1.342 | -2.3186 | -1.2616 | -2.3481 | -3.1471 |
| LOC_Os09g38130 | -0.9768 | n.s. | n.s. | n.s. | n.s. | n.s. | n.s. |
| LOC_Os09g38320 | n.s. | n.s. | n.s. | n.s. | -0.9638 | n.s. | n.s. |
| LOC_Os09g39620 | n.s. | n.s. | n.s. | n.s. | n.s. | -1.0748 | n.s. |
| LOC_Os09g39910 | n.s. | n.s. | n.s. | n.s. | n.s. | -0.7892 | -1.0187 |
| LOC_Os09g39960 | n.s. | n.s. | n.s. | n.s. | n.s. | -0.6023 | n.s. |
| LOC_Os10g02880 | n.s. | 2.3132 | n.s. | n.s. | n.s. | 2.1136 | n.s. |
| LOC_Os10g17489 | n.s. | n.s. | -1.6514 | n.s. | -1.3592 | -1.9687 | -1.1659 |
| LOC_Os10g20470 | n.s. | n.s. | -0.8199 | n.s. | n.s. | -1.5567 | n.s. |
| LOC_Os10g22520 | n.s. | n.s. | n.s. | n.s. | n.s. | 0.8788 | n.s. |
| LOC_Os10g25210 | n.s. | n.s. | n.s. | n.s. | n.s. | -0.6532 | n.s. |
| LOC_Os10g25400 | n.s. | n.s. | n.s. | n.s. | n.s. | -1.1608 | -1.6234 |
| LOC_Os10g28000 | n.s. | n.s. | -0.9614 | n.s. | n.s. | -1.8164 | n.s. |
| LOC_Os10g30790 | n.s. | n.s. | n.s. | n.s. | n.s. | -0.4857 | -0.7911 |
| LOC_Os10g32680 | n.s. | n.s. | n.s. | n.s. | 0.8879 | n.s. | -1.7467 |
| LOC_Os10g35070 | n.s. | n.s. | n.s. | n.s. | n.s. | n.s. | -1.2868 |
| LOC_Os10g38040 | n.s. | n.s. | n.s. | -1.2738 | n.s. | -0.638 | -2.2726 |
| LOC_Os10g38470 | n.s. | n.s. | n.s. | n.s. | n.s. | -0.6138 | n.s. |
| LOC_Os10g38489 | n.s. | n.s. | n.s. | n.s. | n.s. | -0.866 | n.s. |
| LOC_Os10g38610 | n.s. | n.s. | n.s. | 1.1314 | n.s. | -1.4074 | n.s. |
| LOC_Os10g38740 | n.s. | n.s. | n.s. | n.s. | n.s. | -2.3995 | n.s. |
| LOC_Os10g39870 | n.s. | n.s. | n.s. | n.s. | n.s. | -1.2383 | n.s. |
| LOC_Os10g39920 | n.s. | n.s. | n.s. | n.s. | n.s. | -1.059 | n.s. |
| LOC_Os10g40360 | 0.8496 | n.s. | n.s. | 1.6851 | n.s. | -0.6828 | 1.1519 |
| LOC_Os10g40490 | n.s. | n.s. | -0.7177 | n.s. | n.s. | -1.2344 | n.s. |
| LOC_Os10g41550 | n.s. | n.s. | n.s. | -4.0957 | n.s. | n.s. | -4.3846 |
| LOC_Os10g41930 | n.s. | n.s. | -0.9632 | n.s. | n.s. | -0.9225 | n.s. |
| LOC_Os10g42320 | n.s. | n.s. | n.s. | n.s. | n.s. | -0.6674 | n.s. |
| LOC_Os10g43060 | 1.2173 | n.s. | -0.793 | n.s. | -1.1384 | -1.2193 | n.s. |
| LOC_Os11g02100 | n.s. | n.s. | n.s. | n.s. | n.s. | 1.4387 | n.s. |
| LOC_Os11g04104 | n.s. | n.s. | n.s. | n.s. | n.s. | -1.3564 | -0.8525 |
| LOC_Os11g04860 | n.s. | n.s. | n.s. | n.s. | n.s. | 0.7383 | 1.4346 |
| LOC_Os11g06770 | n.s. | n.s. | -1.0399 | -1.2461 | -0.7986 | -2.7976 | -2.0734 |
| LOC_Os11g07440 | 0.9677 | n.s. | -0.9512 | -1.2928 | n.s. | n.s. | n.s. |
| LOC_Os11g07960 | n.s. | n.s. | n.s. | n.s. | n.s. | n.s. | 2.5592 |
| LOC_Os11g18570 | -1.5921 | n.s. | n.s. | n.s. | n.s. | n.s. | n.s. |
| LOC_Os11g23080 | n.s. | n.s. | n.s. | n.s. | n.s. | -1.2148 | n.s. |
| LOC_Os11g35040 | n.s. | n.s. | n.s. | n.s. | n.s. | n.s. | 1.1743 |
| LOC_Os11g47600 | n.s. | -2.4396 | n.s. | n.s. | n.s. | -1.6328 | n.s. |
| LOC_Os12g03899 | n.s. | n.s. | n.s. | n.s. | n.s. | -1.1192 | n.s. |
| LOC_Os12g05050 | 0.8264 | n.s. | n.s. | 0.6956 | 0.6418 | n.s. | 0.7788 |
| LOC_Os12g05210 | n.s. | 3.4062 | n.s. | n.s. | n.s. | n.s. | n.s. |
| LOC_Os12g07030 | 1.661 | n.s. | n.s. | n.s. | -0.7422 | -0.7039 | -1.6264 |
| LOC_Os12g16080 | n.s. | n.s. | n.s. | n.s. | n.s. | n.s. | 1.771 |
| LOC_Os12g29400 | n.s. | n.s. | n.s. | n.s. | n.s. | -1.7403 | -1.8592 |
| LOC_Os12g37840 | n.s. | n.s. | n.s. | -1.4696 | n.s. | n.s. | n.s. |

Numeric values are log_2_ fold change high temperature relative to normal temperature; *n.s.* indicates the gene was not significantly differentially expressed. Genes are up-regulated following SA treatment, identified from Garg R, *et al.* (Plant Signaling & Behavior. 2012;7(8):951-6).
