# Supplementary material for: RNA-Seq analysis reveals insight into enhanced rice Xa7-mediated bacterial blight resistance at high temperature
Source: PLoS One. 2017 Nov 6;12(11):e0187625. doi: 10.1371/journal.pone.0187625 (PMC5673197; doi:10.1371/journal.pone.0187625)
Supplement: S8 Table — (DOCX) [file pone.0187625.s010.docx]

**Table S8:** Odds ratios of promoter motifs in the promoters of different gene sets.

|  | | **ABRE** | **TATA** | **IBOX** | **GCBP2** | **TELO** |
| --- | --- | --- | --- | --- | --- | --- |
| **3 hpi** | Sus up | 1.47 | 1.64 | *n.e.* | *n.e.* | *n.e.* |
|  | Sus dn | 0.61 | *n.e.* | 2.03 | *n.e.* | *n.e.* |
|  | Res up | *n.e.* | *n.e.* | *n.e.* | *n.e.* | 0.72 |
|  | Res dn | 1.39 | 1.31 | *n.e.* | *n.e.* | 0.68 |
| **12 hpi** | Sus up | *n.e.* | *n.e.* | *n.e.* | *n.e.* | 0.76 |
|  | Sus dn | *n.e.* | 1.23 | *n.e.* | 1.18 | 0.70 |
|  | Res up | *n.e.* | 0.86 | 1.22 | *n.e.* | 0.68 |
|  | Res dn | 1.14 | 1.23 | 0.83 | 1.12 | *n.e.* |
| **24 hpi** | Sus up | *n.e.* | *n.e.* | *n.e.* | *n.e.* | 0.38 |
|  | Sus dn | 1.27 | 1.40 | *n.e.* | 1.25 | 1.87 |
|  | Res up | *n.e.* | 1.18 | *n.e.* | 0.87 | 0.50 |
|  | Res dn | 1.21 | 1.19 | *n.e.* | 1.26 | 1.32 |

An odds ratio >1 indicates that the motif was significantly enriched in the upstream promoters of the genes in the given set (Sus = susceptible, Res = resistant, up = upregulated genes, dn = downregulated genes) when compared to a random set of 10,000 rice genes, while an odds ratio <1 indicates that’s the motif was significantly underrepresented in the promoters of the genes in the given set when compared to the random set of 10,000 rice genes. n.e. = not statistically enriched relative to the random set of genes. Statistical enrichment was determined by Fisher’s exact test (p < 0.05).
